# Diversity and Multi-Target Potential of Pyrazole, Imidazole or Triazole Derivatives in Modern Anticancer Therapy

**DOI:** 10.3390/ijms27104172

**Published:** 2026-05-07

**Authors:** Martyna Mysłek, Anna Kaczmarek, Piotr Świątek, Żaneta Czyżnikowska

**Affiliations:** 1 Department of Basic Chemical Sciences, Faculty of Pharmacy, Wroclaw Medical University, Borowska 211a, 50-556 Wroclaw, Poland; martyna.myslek@student.umw.edu.pl; 2 Department of Medicinal Chemistry, Faculty of Pharmacy, Wroclaw Medical University, Borowska 211a, 50-556 Wroclaw, Poland; anna.kaczmarek@umw.edu.pl

**Keywords:** multi-target directed ligands, anticancer therapy, nitrogen heterocycles, imidazole, pyrazole, triazole

## Abstract

Cancers are intricate and multifactorial diseases. Despite progress in medicine, there are still some obstacles in their treatment due to drug resistance, the toxicity of combination therapy and lack of drug selectivity toward cancer cells. The solution to this may be multi-target directed ligands (MTDLs), which have gained more and more popularity over the years. This review presents a comprehensive overview of novel potential multi-targeted derivatives of nitrogen-containing heterocycles, as imidazole, pyrazole, 1,2,3-triazole and 1,2,4-triazole. The review gathers the selected literature from 2006 to 2026. The analysis focuses on the potency of the inhibitory activity of selected molecules against a variety of molecular targets, as well as on their interactions with protein binding sites. Additionally, the structure-activity relationship (SAR) studies within the collected series are included. The discussion may contribute to the development of new multi-target anticancer agents.

## 1. Introduction

Cancer remains one of the most significant global health challenges of the 21st century. Statistics from the International Agency for Research on Cancer (IARC) for the year 2022 showed that there were around 20 million new cases of cancer and around 9.7 million deaths. According to the data, on average, one in five people in the world may develop cancer during their lifetime [1]. The observed increase in cancerous cases is consistent with population ageing and socio-economic development, and is indicative of changes in lifestyle and environmental exposure. Despite the valuable progress that has been made in the fields of early diagnosis and targeted therapy, drug resistance and metastasis limit the effectiveness of therapeutic approaches. Therefore, the design of new chemical compounds capable of modulating biological targets through non-traditional interaction patterns has become a major focus of current drug discovery efforts. In recent years, the development of multi-targeted therapies has strengthened optimism in the fight against cancer. Multi-target directed ligands (MTDLs) can modulate several signaling pathways simultaneously. Such an approach has proven effective in treating cancers and complex diseases with multifactorial etiology, including neurodegenerative disorders, cardiovascular diseases, and chronic inflammatory conditions. The therapeutic potency of a multi-target agent is enhanced by synergistic effects associated with the simultaneous inhibition of activation of multiple key enzymes, receptors, or transcription factors. Furthermore, to achieve the desired pharmacological effect, such compounds require lower doses, which significantly reduces undesirable side effects [2,3].

Since traditional treatment methods are often insufficient, combination of different drugs or multi-target directed ligands are currently used to improve the effectiveness of therapy [4,5]. These strategies are characterized by improved efficacy and enhanced therapeutic outcomes, resulting from their synergistic anticancer effects and their ability to combat drug resistance development [6]. However, combination therapies also have some drawbacks. They may cause various side effects as a result of drug-drug interactions [6]. Furthermore, finding and developing effective combinations can be challenging and time-/cost-consuming [7]. In contrast, MTDLs are single chemical compounds simultaneously aiming at multiple receptors or enzymes.

To date, the Food and Drug Administration (FDA) has already approved some MTDLs for the treatment of various types of cancer. The first one to be approved was imatinib in 2001, which inhibits Bcr-Abl and c-KIT, for the treatment of malignant metastatic and/or unresectable gastrointestinal stromal tumors (GISTs) [8]. After imatinib, other well-known multi-target directed ligands were, for instance, sorafenib, which is used for advanced renal cell carcinoma (RCC) [9], unresectable hepatocellular carcinoma (HCC) [10] or advanced radio-iodine-refractory differentiated thyroid cancer [11], and sunitinib, which is used for advanced RCC [12] or advanced pancreatic neuroendocrine tumors (pNETs) [13].

Heterocyclic compounds have been recognized as fundamental building blocks in medicinal chemistry due to their wide range of biological activities. The dynamic electronic structure and conformational versatility of heterocycles make them attractive candidates for the development of new therapeutic agents. The presence of heteroatoms such as nitrogen, oxygen, and sulfur enables hydrogen bonding and metal coordination. It also modulates lipophilicity, resulting in precise control over molecular recognition and pharmacokinetic behavior. They have been demonstrated to exhibit a variety of pharmacological properties, including anti-inflammatory, antifungal, antiviral, and antibacterial, as well as anticancer properties [14]. On the other hand, when improperly used or managed, heterocyclic pesticides and pharmaceuticals may cause toxicity in living organisms due to their persistence, good solubility and tendency to bioaccumulate. Studies have shown that prolonged exposure to certain heterocycles and their by-products may trigger health problems such as neurotoxicity, metabolic toxicity or genotoxicity [15,16,17].

Hybrid systems integrating multiple heterocyclic motifs have emerged as a particularly promising approach in designing anticancer agents able to inhibit proliferation, angiogenesis, and metastasis through multiple mechanisms of action. Such compounds can simultaneously modulate several signaling pathways. The structural diversity of heterocyclic scaffolds is crucial for discovering new multi-target agents. In oncology, heterocycles serve as the key structural framework for numerous drugs that have been approved for clinical use [18,19]. Their structural versatility provides opportunities to modulate physicochemical properties, metabolic stability, and target selectivity. Five-membered nitrogen-containing rings, such as triazoles, imidazoles, and pyrazoles, are often the building blocks of kinase inhibitors, whereas quinolines, indoles, and isoquinolines are present in compounds that target topoisomerases and other DNA-interacting enzymes [20]. Notably, nitrogen-containing heterocycles mimic endogenous nucleobases, facilitating their incorporation into DNA [21].

Examples of anticancer drugs that incorporate five-membered nitrogen-containing rings include lorlatinib (for ALK-positive non-small cell lung cancer NSCLC; inhibition of anaplastic lymphoma kinase ALK and c-Ros oncogene ROS1) [22], axitinib (for advanced RCC; inhibition of vascular endothelial growth factor VEGFR-1, -2 and -3) [23,24] and entrectinib (for neurotrophic tyrosine receptor kinase NTRK, ROS1 and ALK fusion-positive solid tumors; inhibition of tropomyosin receptor kinase TRKA, TRKB, TRKC, ROS1, ALK) [25].

Derivatives of five-membered nitrogen heterocycles seem to be an excellent choice as potential MTDLs. They can form diverse interactions with many enzymes and receptors. Additionally, they may act as MTDLs by combining with other pharmacophores either directly (by fusing or merging) or indirectly (via a linker). Some five-membered aromatic systems containing nitrogen atoms are also used as linkers between two active moieties aiming at different molecular targets.

This review covers the selected literature published over the past 20 years. While numerous recent reviews have provided comprehensive analyses of individual heterocyclic scaffolds such as pyrazoles and triazoles, these studies remain largely scaffold-centered and do not address the problem from a multi-target drug-design perspective [26,27,28,29]. Unlike previously published scaffold-focused reviews that analyze individual heterocyclic classes (e.g., pyrazoles or triazoles), the present work adopts a MTDL-oriented perspective, integrating multiple heterocyclic scaffolds within a unified framework of anticancer drug design. This approach enables the identification of cross-scaffold design principles and functional roles of heterocycles in multi-target engagement, which are not accessible in conventional scaffold-specific analyses.

## 2. Five-Membered Heterocyclic Compounds with Two or Three Nitrogen Atoms with Multi-Target Activity

### 2.1. Imidazole-Based Compounds

**Figure 1:**
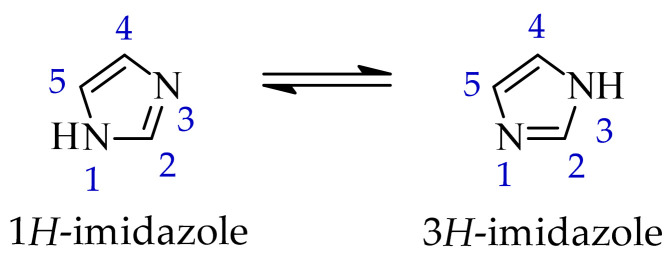
Two isomeric forms of imidazoles [30].

In the class of five-membered nitrogen-containing heterocycles, imidazole is a versatile structural motif, widely present in both natural and synthetic compounds. The characteristic feature of this compound is the presence of two non-equivalent nitrogen atoms, located at positions 1 and 3 of the aromatic ring. The imidazole ring is capable of prototropic tautomerism involving proton transfer between the two nitrogen atoms of the ring. In principle, two aromatic tautomeric forms can be distinguished, namely 1*H*- and 3*H*-imidazole (Figure 1). However, in the unsubstituted system, these forms are energetically comparable. Consequently, under standard conditions, it is generally not possible to unambiguously assign a single tautomeric form [30]. One nitrogen atom has properties analogous to those in pyridine and acts as a proton acceptor, while the other is pyrrole-like and has a proton-donating ability. The structural and electronic flexibility of the imidazole scaffold enables it to engage in π-π stacking and hydrogen bonding, facilitating efficient molecule recognition by biological macromolecules.

**Figure 2:**
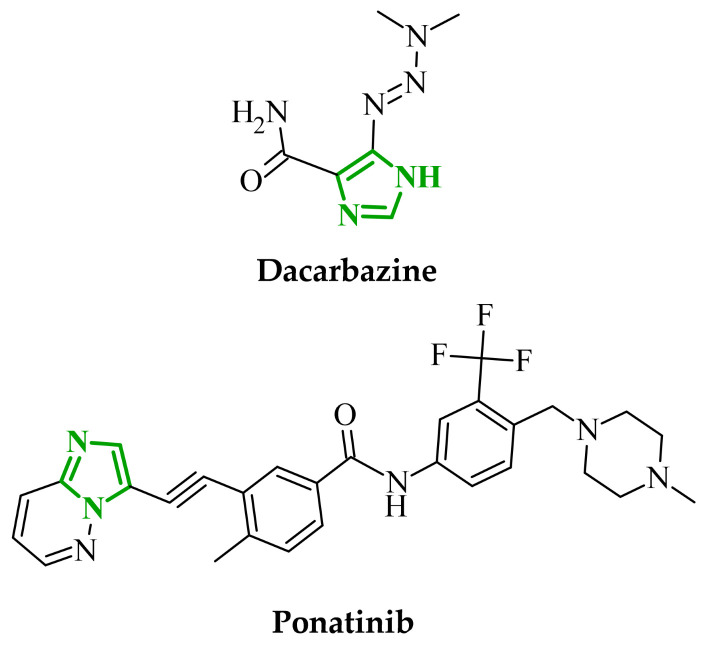
Selected examples of drugs incorporating the imidazole fragment [36,37].

The presence of imidazole in biologically relevant molecules indicates its importance in enzymatic catalysis, metal coordination, and physiological pathways. Due to their broad spectrum of biological activities, imidazole and its derivatives attract considerable attention from researchers in medicinal and pharmaceutical chemistry [31]. Numerous studies have demonstrated that imidazole-based compounds exhibit anticancer [31], antituberculosis [32], antibacterial [33], and anti-inflammatory [34] activities. Imidazole-based drugs are used in the treatment of a wide range of diseases. Metronidazole and ketoconazole are extensively used in anaerobic bacterial and fungal infections due to their ability to interact with microbial DNA and inhibit cytochrome P450-dependent enzymes, respectively [2,35]. Compounds containing imidazole and/or fused imidazole in their structure are also permitted for cancer therapy. A notable example is dacarbazine (Figure 2), which has proven effective in the treatment of metastatic malignant melanoma or Hodgkin’s disease as a secondary-line therapy [36]. Another example of this is ponatinib (Figure 2), which contains an imidazole fused with pyridazine. The medication under discussion has been approved by the FDA for the treatment of chronic myeloid leukemia and acute lymphoblastic leukemia [37]. As a five-membered heterocycle containing two nitrogen atoms, the imidazole ring has the capacity to act as both a hydrogen-bond donor and an acceptor. This property facilitates interactions with biomolecules and influences the solubility and distribution of compounds within the body [31]. The metabolism of drugs containing heterocyclic nitrogen systems occurs primarily in the liver, with the participation of cytochrome P450 enzymes. A case in point is dacarbazine, which functions as a prodrug and undergoes metabolic transformations, resulting in the formation of the active metabolite monomethyltriazene, which is responsible for its biological activity [38]. In turn, ponatinib undergoes primarily oxidative metabolism, catalyzed by enzymes of the CYP450 family, including reactions such as *N*-dealkylation. This process leads to further metabolism and elimination of the compound from the body. Adverse effects related to cardiovascular toxicity have been observed during therapy, and it is therefore possible that such effects may also be experienced by the patient [39].

Recent studies demonstrated that derivatives based on imidazole scaffold exhibit dual or multidirectional inhibitory activity that can simultaneously modulate key signaling pathways. The multitarget potential provides a promising strategy to overcome the limitations of single-target therapy and improve therapeutic potency. One of the key molecular pathways influenced by imidazole-based anticancer compounds involves the regulation of angiogenesis through the vascular endothelial growth factor (VEGF) signaling cascade. Vascular endothelial growth factors and their tyrosine kinase receptors are important regulators of angiogenesis. Since tumor vascularization plays an essential role in cancer progression, inhibiting the VEGF/VEGFR signaling pathway becomes a promising therapeutic approach [40].

**Figure 3:**
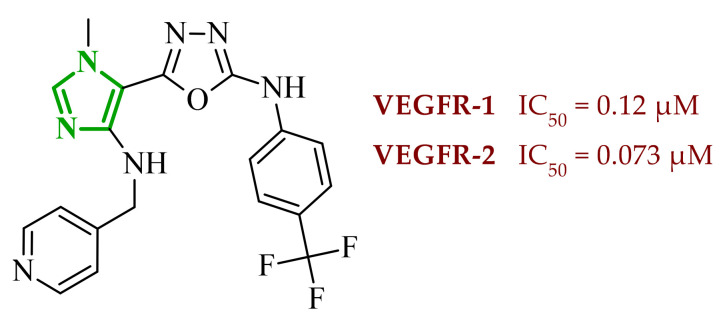
The structure of the most active heteroaryl imidazole derivative (**1**)-a potential VEGFR-1 and VEGFR-2 inhibitor [41].

Kiselyov et al. reported a series of heteroaryl imidazole-based derivatives designed as potent inhibitors of vascular endothelial growth factor receptors. Their study demonstrated that required pharmacophoric interactions for kinase inhibitory activity are dependent on substitution patterns on the imidazole scaffold [41]. Among all synthesized compounds, several exhibited strong inhibition of both VEGFR-1 and VEGFR-2, confirming the dual target potential of the scaffold. According to results obtained for the most potent analogues, the IC_50_ values ranged from 0.043–0.130 µM for VEGFR-2 and 0.120–0.590 µM for VEGFR-1. The most potent dual inhibitor toward VEGFR-1 and VEGFR-2 was identified as compound **1** (17-compound number from the source publication), featuring a *p*-trifluoromethyl substituted aniline ring and 4-pyridyl moiety (Figure 3). The structure-activity relationship analysis revealed that electron-withdrawing substituents (Cl, CF_3_, OCF_3_) in the *para* or 3,4-positions significantly enhance inhibitory potency toward both enzymes. Importantly, compounds with less favorable substitution patterns showed reduced inhibitory activity. The *para*-substituted aniline derivatives such as *p*-Cl, *p*-*tert*-Bu, and *p*-iso-Pr displayed only moderate VEGFR-2 inhibition, with IC_50_ values in the range 0.25–0.44 µM. On the other hand, *meta*-substituted analogues resulted in further loss of activity. The introduction of bulky *ortho*-substituents resulted in a dramatic decrease or complete loss of enzymatic inhibition, with IC_50_ values exceeding 10 µM. Similarly, incorporation of larger anilinic moieties such as phenyl or phenoxy groups leads to a pronounced reduction in activity, yielding compounds with weak or negligible inhibition (IC_50_ > 1–3 µM), mainly due to the steric clashes and limited accommodation into the hydrophobic ATP-binding pocket. What is important, selected imidazole derivatives demonstrated consistent activity in both enzymatic and cell-based assays with the most potent compounds inhibiting VEGRF-2 phosphorylation at submicromolar concentrations. The compounds also exhibited good aqueous solubility (>mM) supporting their potential for further development.

**Figure 4:**
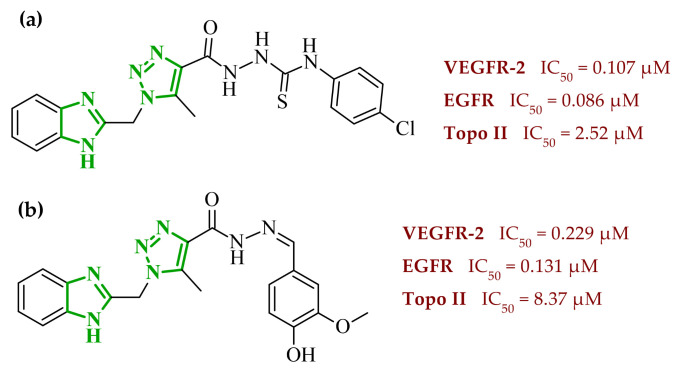
The structures of the most active benzimidazole-triazole hybrids: (**a**) **2**; (**b**) **3**-potential VEGFR-2, EGFR and Topo II inhibitors [42].

Interestingly, imidazole-related heterocyclic systems, such as benzimidazole, also demonstrated affinity toward VEGFR-2, while exhibiting additional activity on other molecular targets. For example, Othman et al. reported a new series of benzimidazole-triazole hybrids acting as multitarget inhibitors of VEGFR-2, epidermal growth factor receptor (EGFR), and topoisomerase II (Topo II) [42]. Among the synthesized compounds, those bearing a 4-chloro substituent within the aryl fragment of the carbothio(oxa)amide derivatives (referred to as **2** (5a)) and those containing the 4-hydroxy-3-methoxy arylidene fragment (denoted as **3** (6g)) demonstrated the highest cytotoxic activity across the tested cancer cell lines (Figure 4). Compound **2** showed approximately 45% of the inhibitory activity of sorafenib against VEGFR-2. In contrast, compound **3** showed weaker inhibition of VEGFR-2, accounting for only 21% of the activity of sorafenib.

Data obtained from a molecular docking study showed that both compounds bind in the typical binding mode of VEGFR-2 inhibitors. The benzimidazole fragment in each case penetrates the same hydrophobic pocket, forming contacts with residues such as Leu1033 and Ala864. Additional hydrophobic contacts are also observed with the aromatic residues Phe916 and Phe1045, which further contribute to binding stabilization. Interestingly, both compounds were also established to inhibit the epidermal growth factor receptor (EGFR), another important molecular target involved in tumor progression. EGFR is a transmembrane tyrosine kinase receptor that plays a crucial role in regulating cell proliferation, differentiation, and survival [43]. Its overexpression has been associated with the development of various malignancies. In the study, compound **2** demonstrated strong inhibitory activity toward EGFR corresponding to approximately 60% of the potency of gefitinib, while **3** showed moderate activity, around 40% of reference drug potency. The binding mode of compound **2** revealed key hinge interactions. Benzimidazole moiety in this case binds to the cavity created with Leu718, Met793, Asp800, Leu844. Compound **3**, on the other hand, extends beyond the optimal binding pocket, resulting in weaker hydrophobic and aromatic contacts, which can explain its lower inhibitory activity. Structure-activity relationship analysis further demonstrated that the five-atom linker was preferable over the four-atom linker in the first compound series, while a chlorine substituent at the *para*-position of the phenyl ring was important for optimal activity. According to data, unsubstituted phenyl rings or methyl-substituted analogues exhibited a pronounced loss of potency. In the second series of compounds, the presence of electron-donating substituents exerted a favorable effect on activity. The electron-withdrawing groups, such as 4-chloro, 4-bromo, or 3-nitro, resulted in diminished inhibitory profiles. In addition to kinase inhibition, the anticancer potential of these compounds was further supported by their ability to inhibit topoisomerase II, a key enzyme involved in DNA replication and repair. The inhibition of Topo II prevents re-ligation of DNA, leading to double-strand breaks [44]. As presented, compound **2** demonstrated strong Topo II inhibitory activity, exceeding that of doxorubicin. Compound **3** exhibited weaker inhibition corresponding to 43% of doxorubicin activity. Docking results indicated that compound **2** adopts a binding orientation characteristic of ATP-competitive inhibitors, fitting within the ATP-binding pocket. The benzimidazole moiety occupied that cavity, establishing hydrophobic contacts with Asn91, Ile125 and Phe142. In the case of compound **3**, benzimidazole core still occupies the region corresponding to the triphosphate binding site, creating hydrogen bonds with Arg162, Asn163 and Gly164, as well as hydrophobic contacts with Gly166 and Lys 378 amino acids residues. Further biological investigations revealed that their anticancer activity is associated with DNA intercalation, induction of apoptosis, and cell cycle arrest at distinct phases (S phase for **2** and G2/M for **3**), supporting a multi-pathway mode of action. The most active compounds demonstrated consistent cytotoxic activity across several cancer cell lines, accompanied by significantly lower toxicity towards normal WI-38 cells and improved selectivity indices compared to doxorubicin. Additionally, physicochemical evaluation of the most active compounds indicated compliance with Lipinski’s rule of five, with no significant violations observed. Compared to doxorubicin, the analyzed derivatives exhibited more favorable calculated parameters, including lower polar surface area and balanced lipophilicity, which may support improved membrane permeability.

**Figure 5:**
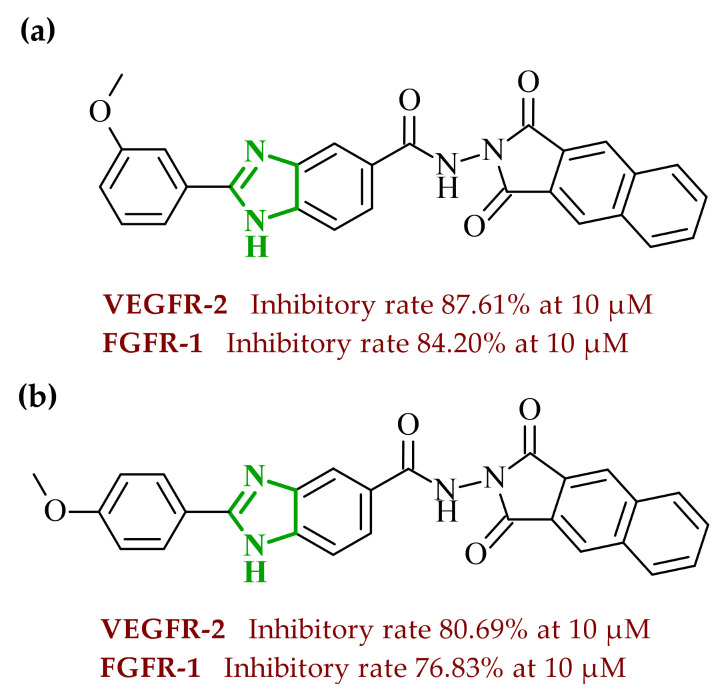
The structures of the most active benzimidazole-dioxyisoindoline hybrids: (**a**) **4**; (**b**) **5**-potential VEGFR-2 and FGFR-1 inhibitors [45].

The observations of Othman and coworkers highlight the versatility of the benzimidazole scaffold. Similarly, Abdel-Mohsen and Nageeb proposed a series of the benzimidazole hybrids evaluated as inhibitors of VEGFR-2 and fibroblast growth factor receptor (FGFR-1) [45]. First, they examined the benzimidazole-dioxoindoline scaffold. According to their study, the inhibitory activity toward VEGFR-2 was strongly governed by both the nature of the substituent attached to the benzimidazole core and to the dioxoindoline moiety. The initial screening assays revealed at a concentration of 10 µM pronounced structure-activity relationships across the series. Within the series, the 4-methoxyphenyl derivative was the most active compound displaying 71.11% inhibition of VEGFR-2, whereas replacement of the *para*-methoxy group with a 3-methoxy, 2,5-dimethoxy, or 3,4,5-trimethoxy substituent resulted in a marked decrease in activity (37.94–48.90% inhibition). In contrast, substitution with a 4-chlorophenyl group led to dramatic loss of activity, yielding only 5.23% of inhibition, indicating that electron-donating substituents at the *para* positions are strongly favored over electron-withdrawing groups. The introduction of a carboxylic group at the 5-position of the dioxoisoindoline moiety exerted a context-dependent effect on VEGFR-2 inhibition. For example, the introduction of the carboxylic group enhanced the activity of an initially weak analogue. The carboxy derivative showed 53.54% inhibition of VEGFR-2 compared to only 5.23% for the unsubstituted compound. On the other hand, if the same structural modification was made in the group of analogues showing moderate to high potency, it resulted in a reduction in the inhibitory effects (with an average inhibition of ~32%). A substantial improvement in VEGFR-2 inhibition was observed upon replacement of the 1,3-dioxoisoindolin-2-yl moiety with the bulkier 1,3-dioxo-1,3-dihydro-2*H*-benzo[*f*]isoindol-2-yl moiety. Such modification led to a general increase in activity, with compounds **4** (8l) and **5** (8m) (Figure 5) displaying the highest inhibition levels. Notably, the 3,4,5-trimethoxy derivative showed very weak activity indicating that extensive substitution on the aryl ring is unfavorable for VEGFR-2 inhibition.

In addition to enzymatic inhibition, the most active derivative (**5**) demonstrated relevant cellular activity across a broad panel of cancer cell lines (NCI-60), with particularly pronounced effects against leukemia, melanoma, and breast cancer models, reaching growth inhibition values up to approximately 90%. Further evaluation confirmed moderate cytotoxic potency toward the MCL-7 cell line, indicating that the multitarget kinase inhibition is translated into cellular activity. Compound **5** induces cell cycle arrest at the G2/M phase and promotes apoptosis, accompanied by a measurable anti-migratory effect in wound healing assays. Molecular docking of compound **5** toward VEGFR-2 showed that the NH-group of the benzimidazole ring forms a stabilizing hydrogen bond with Glu885, while the C=N fragment interacts with Asp1046, positioning the scaffold in the proximity of the aspartate-phenylalanine-glycine (DFG) region. The benzimidazole core also engages in a network of hydrophobic contacts with Leu889, Val899, Cys1045, and Phe1047. This contributes to the stabilization of the ligand within the hydrophobic pocket adjacent to the ATP binding site. Moreover, the aryl substituent of the benzimidazole moiety is localized deep within the hydrophobic cavity created by Ile888, Leu889, and Ile892. The designed benzimidazole-dioxyisoindoline hybrids also exhibited a measurable affinity for FGFR-1, a tyrosine kinase receptor involved in regulating cell proliferation and differentiation, by initiating signaling pathways after binding to appropriate growth factors [46]. It was found that at a concentration of 10 µM, compounds **4** and **5** inhibited FGFR-1 only slightly less than sorafenib. Similarly, to their VEGFR-2 activity, the 3-methoxy derivative (**4**) exhibited marginally higher potency than its 4-methoxy analogue, indicating that subtle positional changes of electron-donating groups can modulate dual kinase inhibition. The analysis of docking results showed that the benzimidazole ring of **4** is located near the DFG region, forming a hydrogen bond with the Asp641 amino acid residue. Additionally, the NH group forms a hydrogen bond with Glu531 located in the ATP-binding cleft. In this position, the benzimidazole core also engages in hydrophobic contacts with Ile545, Val561, Ala640, and Phe642. Finally, the 4-methoxyphenyl moiety at the second position of the benzimidazole ring is located in the hydrophobic pocket, forming stabilizing contacts with Cys619 and His621 of FGFR-1. Overall, these findings demonstrate that optimal VEGFR-2/FGFR-1 inhibition within this scaffold requires a fine balance between electronic effects and steric constraints. While *para* and *meta* methoxy substitution on the aryl ring proved beneficial, chlorine substitution, excessive methoxylation and isoindoline scaffold modifications resulted in diminished activity. From a developability perspective, in silico ADME analysis indicated that most of the compounds comply with Lipinski’s rule of five and exhibit favorable gastrointestinal absorption. Additionally, the lack of predicted blood-brain barrier penetration suggests a reduced risk of central nervous system-related side effects. The compounds also demonstrated acceptable bioavailability scores and synthetic accessibility, supporting their potential as drug-like candidates. Selected derivatives showed predicted interactions with P-glycoprotein, which may influence their pharmacokinetic behavior.

**Figure 6:**
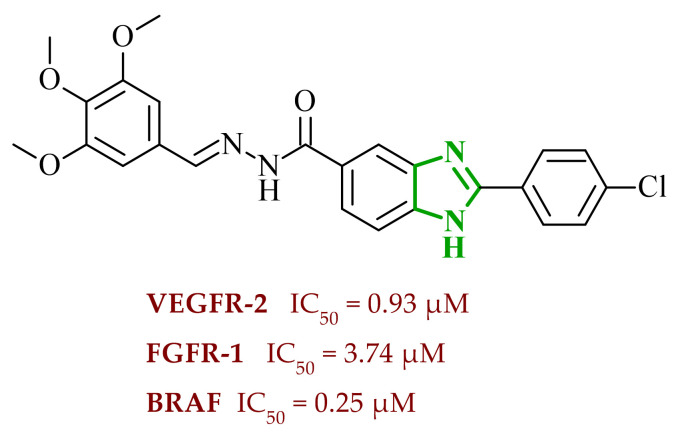
The structure of the most active 2,5-substituted benzimidazole derivative (**6**)-a potential VEGFR-2, FGFR-1 and BRAF inhibitor [47].

In an earlier study, Abdel-Mohsen et al. reported a series of 2,5-disubstituted benzimidazole derivatives designed as multitarget kinase inhibitors of VEGFR-2, FGFR-1, and B-rapidly accelerated fibrosarcoma (BRAF) [47]. They developed a series of benzimidazole derivatives introducing systematic variations in the aromatic substituents to achieve optimal interactions with the selected molecular targets and further explore structure-activity relationships. The SAR analysis revealed that the aromatic substitution at the 2-position of the benzimidazole core was essential for achieving balanced multitarget activity. In particular, the presence of a phenyl moiety favored strong hydrophobic interactions within the allosteric pocket of the kinases, whereas a less optimal or overly substituted aromatic system resulted in diminished inhibitory potency. Molecular docking studies supported these findings, demonstrating that the phenyl substituent stabilizes compound-protein complexes through hydrophobic interactions, while benzylidene-hydrazine fragment forms hydrogen bonds with the hinge region. Consistent interaction patterns were observed across all three kinases, involving key residues such as Cys919 in VEGFR-2, Ala564 in FGFR-1, and Cys532 in BRAF, accompanied by favorable binding energies. The obtained binding energy values were also favorable, ranging from −14.9 to −12.7 kcal/mol for VEGFR-2, from −14.2 to −11.6 kcal/mol for FGFR-1, and from −13.7 to −11.5 kcal/mol for BRAF. Enzymatic screening identified compound **6** (8u) (Figure 6) as the most promising derivative of the series, exhibiting approximately 80% inhibition of VEGFR-2 at a concentration of 10 μM. Subsequent analyses confirmed its multitarget profile and activity against VEGFR-2, FGFR-1, and BRAF. Studies conducted on cancer cell lines demonstrated that this compound induced cell cycle arrest at the G2/M phase and triggered apoptosis in MCF-7 cells. Results suggested that the observed antiproliferative effects are associated with apoptosis induction and disruption of cell cycle progression. In silico ADME evaluation of the synthesized compounds revealed compliance with Lipinski’s rule of five, with appropriate molecular weight, hydrogen bond donor/acceptor counts, and balanced lipophilicity. The predicted high gastrointestinal absorption suggests favorable permeability, while the lack of blood-brain barrier penetration may indicate a reduced likelihood of central nervous system-related side effects. The calculated bioavailability scores (approximately 0.55) support the drug-like character of the designed derivatives.

**Figure 7:**
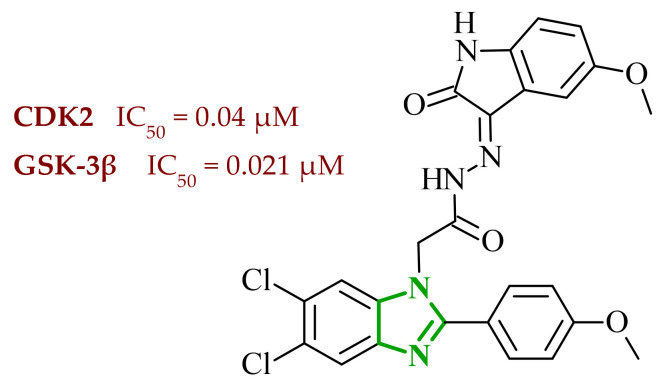
The structure of the most active benzimidazole-oxindole hybrid (**7**)-a potential CDK2 and GSK-3β inhibitor [48].

In their subsequent work, Abdel-Mohsen and co-workers focused on benzimidazole derivatives targeting cyclin-dependent kinase 2 (CDK2) and glycogen synthase 3β (GSK-3β) [48]. CDK2 is a serine/threonine protein kinase that plays a central role in regulating the cell cycle, particularly during the G1/S transition and progression through the S phase, by interacting with cyclins E and A. The dysregulation or overactivation of CDK2 has been associated with uncontrolled cell proliferation and tumor development, making it a valuable target for anticancer drug development [49]. Similarly, GSK-3β is another important intracellular serine/threonine kinase that regulates multiple cellular processes, including apoptosis, metabolism, and cell cycle progression, whose aberrant activity has also been implicated in cancer-related signaling pathways. Although two isoforms of GSK-3 (α and β) are present in humans, the β isoform is more closely associated with cancer progression and neurodegenerative disorders [50]. Based on literature data and existing structure-activity relationship knowledge, the authors proposed a hybrid design strategy combining oxindole and benzimidazole scaffolds to obtain multitarget anticancer agents. These two pharmacophores were linked via an acetohydrazide spacer, which was expected to enhance binding affinity through its ability to act as both a hydrogen bond donor and acceptor. Evaluation of the growth inhibitory activity against a panel of National Cancer Institute NCI cancer cell lines revealed that all tested derivatives exhibited antiproliferative effects, although with varying potencies. Importantly, the obtained data enabled the establishment of a clear structure-activity relationship. In several cases, halogen substitution on the oxindole ring resulted in improved activity compared with methoxy substitution; however, this effect was not consistent across the entire series. Compounds bearing a methoxyphenyl group of benzimidazole generally showed higher activity than their phenyl analogues. This trend highlights the beneficial role of increased hydrophobicity and optimal steric bulk in enhancing biological activity. Enzymatic assays confirmed that the compounds strongly and selectively inhibited CDK2 and GSK-3β kinases, with IC_50_ values in the low micromolar to nanomolar range. Additional experiments performed on PANC-1 and MCF-7 cell lines demonstrated that selected derivatives induce G2/M phase cell cycle arrest and induction of apoptosis. Among the entire series, compound **7** (8v) (Figure 7) exhibited the most promising anticancer and enzymatic properties. Importantly, compound **7** demonstrated a high degree of selectivity, showing a more than 10-fold preference towards CDK2 and GSK-3β over other kinases, including CDK1, CDK5, GSK-3α, VEGFR-2, and BRAF, highlighting its potential for reduced off-target effects. Furthermore, the antiproliferative activity of the synthesized hybrids was validated in the NCI-60 cancer cell panel, where several derivatives exhibited strong growth inhibition at a single dose (10 µM), and selected compounds showed GI_50_ values as low as 0.53 µM in five dose assays, confirming their potent cellular activity. Additionally, selected derivatives exhibited potent cytotoxic activity against specific cancer cell lines, including PANC-1 and MG-63, with IC_50_ values in the low-micromolar range, further supporting their therapeutic potential.

To further elucidate the mechanism of action of the synthesized benzimidazole–oxindole hybrids, molecular docking studies were performed against both kinases. In the case of the CDK2 active site, the compounds were found to occupy the ATP-binding pocket, where complex stabilization was achieved through hydrogen bond formation with key amino acid residues such as Leu83 and Glu81 which play crucial roles in ligand–enzyme interactions. Additional hydrophobic interactions with residues such as Val81, Phe82, Ala31 and Ala144 were present. Also π-π stacking between aromatic rings of the ligands and aromatic residues of the protein further contributed to the stabilization of the ligand–enzyme complex. Similarly, in the GSK-3β binding pocket, the studied compounds formed hydrogen bonds with essential residues, including Asp133 and Val135. The presence of both benzimidazole and oxindole fragments enabled simultaneous donor–acceptor interactions and stabilization through hydrophobic contacts, supporting their potential to act as dual inhibitors of both kinases. In silico ADME profiling indicated that the proposed compounds exhibit favorable drug-like characteristics, including compliance with Lipinski’s rule of five and appropriate physicochemical parameters. In particular, the calculated logP values suggest balanced lipophilicity, which is essential for efficient membrane permeability. Additionally, the predicted high gastrointestinal absorption indicates favorable transport across biological membranes, supporting their potential oral bioavailability. These features, together with suitable molecular size and polarity, highlight the potential of the designed compounds for further pharmacokinetic optimization.

**Figure 8:**
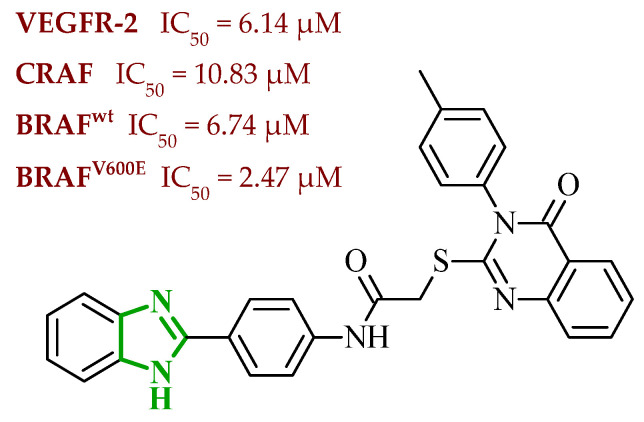
The structure of the most active 2-arylbenzimidazole derivative (**8**)-a potential VEGFR-2 and RAF kinases inhibitor [51].

Another significant contribution to the development of multitarget small-molecule anticancer compounds was provided by Ali et al., who designed a new generation of the 2-arylbenzimidazole-based kinase inhibitors [51]. The synthesized compounds were evaluated against a panel of kinases, including rapidly accelerated fibrosarcoma (RAF) family members [BRAF (wt), BRAF (V600E), and CRAF], VEGFR-2, platelet-derived growth factor receptor β (PDGFR-β), FMS-like tyrosine kinase 3 (FLT-3), and c-KIT, revealing a pronounced preference for RAF kinases over VEGFR-2. BRAF is a serine/threonine kinase that serves as a key activator of MAPK/ERK signaling pathway through MEK (mitogen-activated protein) phosphorylation process. Additionally, it plays a central role in regulating cell proliferation, differentiation, and survival [52]. CRAF protein, despite exhibiting reduced basal kinase activity, plays a pivotal role in mitogen-activated protein kinase (MAPK) pathway signaling through RAS-dependent activation and dimerization with other RAF isoforms [53]. It is closely associated with pathway compensation mechanisms and therapeutic resistance. In this series, the benzimidazole-quinazolinone hybrids emerged as the most potent kinase inhibitors, while the benzimidazole-pyrimidine conjugates displayed generally weak or negligible activity against both wild-type BRAF, as well as VEGFR-2. It is worth noting that in the benzimidazole-pyrimidine series, only the 4-methylpyrimidine derivative exhibited inhibition of BRAF (V600E) with an IC_50_ value of 19.19 µM. In contrast, several compounds from benzimidazole-quinazolinone series exhibited pronounced activity toward RAF kinases (wild-type BRAF (IC_50_ = 0.02–1.22 µM), BRAF (V600E) (IC_50_ = 0.002–0.37 µM), and CRAF (IC_50_ = 0.002–0.14 µM)), in many cases displaying greater potency than sorafenib. The analysis of SAR revealed that replacement of small alkyl substituents with bulkier aromatic groups at the *N*-1 position of the quinazoline moiety significantly enhanced RAF inhibition. On the other hand, *para*-methyl or *para*-methoxy substitution often resulted in reduced potency. It should be mentioned that *para*-chlorophenylquinazoline derivative demonstrated preferential activity toward BRAF (V600E), showing higher selectivity over BRAF (wt) and CRAF, which rationalizes its strong cytotoxicity against melanoma cells. Considering VEGFR-2 inhibition, most compounds exhibited weak activity. The one exception was compound **8** (14k), containing *p*-Me-phenyl substituent, which exhibited the highest VEGFR-2 inhibitory activity within the series (Figure 8). This potency is comparable to the potency of sorafenib. Other analogues, including ethyl, phenyl, and *p*-chlorophenyl substituted derivatives, showed only marginal VEGFR-2 inhibition (IC_50_ > 50–100 µM), reflecting their poor accommodation within the VEGFR binding pocket. In addition to enzymatic inhibition, selected compounds from this series demonstrated cytotoxic activity across multiple cancer cell lines with GI_50_ values in the low micromolar range, indicating effective cellular penetration and intracellular target engagement. The most active derivatives exhibited a clear ability to induce cell cycle arrest at the G2/M phase and promote apoptosis in melanoma cells, confirming that their biological activity extends beyond the target toward functional cellular effects. It is also noteworthy that the majority of compounds displayed a pronounced preference towards RAF kinases over VEGFR-2, suggesting a degree of selectivity that may be advantageous in reducing potential off-target effects associated with broad-spectrum kinase inhibition.

The multitarget activity profile of the benzimidazole-quinazolinone hybrids was further rationalized by molecular docking studies performed for all kinases. Results revealed that the active compounds adopt a binding mode characteristic of type II kinase inhibitors, similar to that observed for sorafenib. In the binding center of all molecular targets, the benzimidazole core was positioned at the interface between the gatekeeper region and the hydrophobic allosteric back pocket, forming key hydrogen bonds with the Glu of αC-helix and the Asp residue of the DFG motif. Additional stabilization was provided by extensive hydrophobic interactions between the benzimidazole aromatic system and residues forming the allosteric pocket of the kinases. Additionally, the quinazolinone fragment was consistently accommodated within the hinge region, forming hydrogen bonds with Cys amino acid residues. Docking scores obtained for the most active RAF inhibitors were comparable to those of sorafenib, particularly in BRAF and CRAF. Slightly weaker binding energies were predicted for VEGFR-2, which agrees with experimentally observed preferential inhibition of RAF kinases.

**Figure 9:**
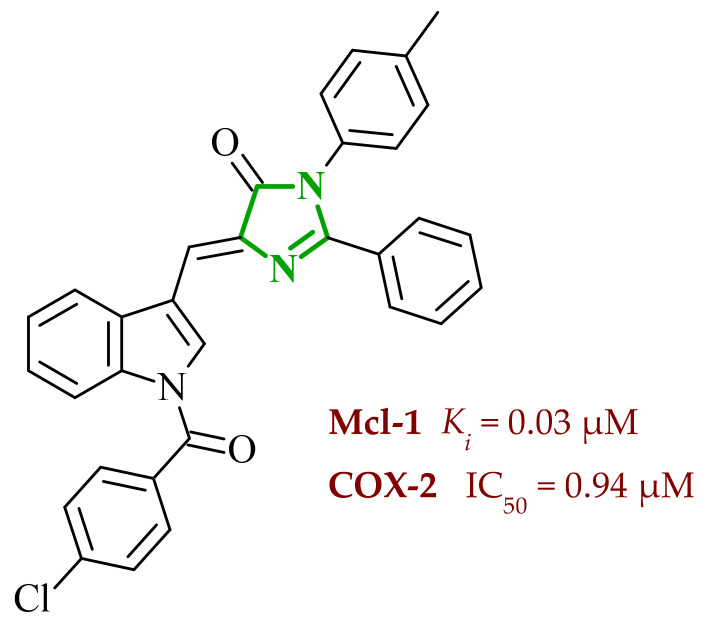
The structure of the most active indole-imidazole derivative (**9**)-a potential Mcl-1 and COX-2 inhibitor [57].

Following the concept of molecular hybridization, Lamie and co-workers designed a series of indole-imidazole hybrids as multitarget agents directed against myeloid cell leukemia-1 protein (Mcl-1) and cyclooxygenase-2 (COX-2) [54]. In recent years, a growing body of research has identified a correlation between inflammation and cancer progression. There are reports of increased COX-2 expression in cancer cells. In addition, a significant proportion of pro-inflammatory mediators are expressed in multiple cancer cells, thereby contributing to the development of the disease. Moreover, it has been demonstrated that overexpression of cyclooxygenase can result in the inhibition of apoptosis and an increase in angiogenesis [55]. Moreover, it has been demonstrated that the use of nonsteroidal anti-inflammatory drugs can reduce the risk of cancer and lower mortality [56]. Mcl-1 belongs to the Bcl-2 protein family and is responsible for the regulation of apoptosis. Its overexpression has been frequently reported in various cancer types, making it an attractive molecular target [57]. Simultaneous targeting of Mcl-1 and COX-2 may lead to a synergistic anticancer and anti-inflammatory effect. Enzymatic evaluation confirmed that all designed compounds exhibited measurable affinity toward Mcl-1, although with pronounced differences in potency. Substitution at the indole *N*-1 position played a crucial role in the modulation of derivatives’ activity. Compounds bearing sulfonyl or benzoyl substituents at this position generally displayed enhanced binding affinity compared to less substituted analogues, indicating that these groups provide an optimal balance between steric bulk and lipophilicity. In particular, compound **9** (6c) (see Figure 9) emerged as the most potent Mcl-1 inhibitor, exhibiting significantly higher potency than the reference compound gossypol (*K_i_* = 0.34 µM). In contrast, derivatives characterized by less favorable substitution patterns showed substantially weaker activity (*K_i_* > 1 μM), highlighting the importance of optimal hydrophobic and steric complementarity for effective engagement of the Mcl-1 binding cavity. Importantly, partial substitution was observed to improve potency compared to weak analogues but was insufficient to achieve the high affinity achieved by optimally substituted derivatives. Molecular docking, along with molecular dynamics simulation results, revealed that the most potent compounds adopted similar binding orientations in the Mcl-1 binding pocket, with complex stabilization dominated by hydrophobic contacts and π-π interactions involving aromatic residues such as Phe228 and Phe270. Additional profiling against other members of the Bcl-2 family revealed that both potency and selectivity toward Mcl-1 within this series are strongly governed by the substitution pattern of the indole-imidazole scaffold. Compound **9** bearing a bulky *p*-chlorobenzoyl group exhibited potent Mcl-1 inhibition while maintaining weaker activity toward Bcl-2 and Bcl-XL (*K_i_* > 12 μM). In contrast, another compound containing a smaller methylsulfonyl substituent, combined with increased alkyl substitution on the aromatic rings, showed enhanced affinity toward Bcl-2 and Bcl-XL (*K_i_* = 5.30 and 7.20 μM), reflecting reduced selectivity. Further cell-based studies confirmed apoptosis induction as a key mode of action.

In parallel, the ability of synthesized compounds to inhibit COX-2 was systematically evaluated. Among the investigated compounds, derivative **9** with *p*-chlorobenzoyl substituent emerged as the most potent COX-2 inhibitor, while remaining markedly less active toward COX-1, resulting in selectivity comparable to celecoxib. In contrast, compound bearing a methylsulfonyl substituent at the indole nitrogen, showed reduced COX-2 potency and lower selectivity over COX-1, suggesting that structural features beneficial for Mcl-1 inhibition are not optimal for effective engagement of COX-2 binding cavity. According to computational results, the most active and selective compound **9** formed stable hydrogen bond interactions with key residues of the COX-2 active site, including Tyr355, while its halogenated aromatic moiety occupied a hydrophobic cavity defined by Val523, Leu352, and Val349, comparable to celecoxib.

In a separate set of experiments, antiproliferative activity was evaluated against three human cancer cell lines representing breast, prostate, and lung cancer. All newly designed molecules demonstrated significant growth-inhibitory effects, with cellular IC_50_ values often lower than those of the reference compound. Considering all obtained results, compound **9** exhibited particularly strong activity against prostate and lung cancer cells (IC_50_ = 5.31 μM and 3.03 μM, respectively). In silico pharmacokinetic profiling revealed that the most active compounds are characterized by high predicted human intestinal absorption (up to 98%), moderate Caco-2 permeability, and strong plasma protein binding, suggesting favorable systemic exposure. Furthermore, the compounds exhibited low to moderate blood-barrier penetration, which may reduce the likelihood of central nervous system-related side effects, while maintaining sufficient bioavailability for systemic anticancer activity. Importantly, metabolism prediction indicated potential inhibition of CYP3A4 and CYP2C9 isoforms, as well as P-glycoprotein interaction, which should be considered during further optimization due to possible drug-drug interactions risk. Toxicity prediction studies suggested a favorable safety profile with no predicted carcinogenicity and only moderate risk of cardiotoxicity associated with hERG inhibition.

**Figure 10:**
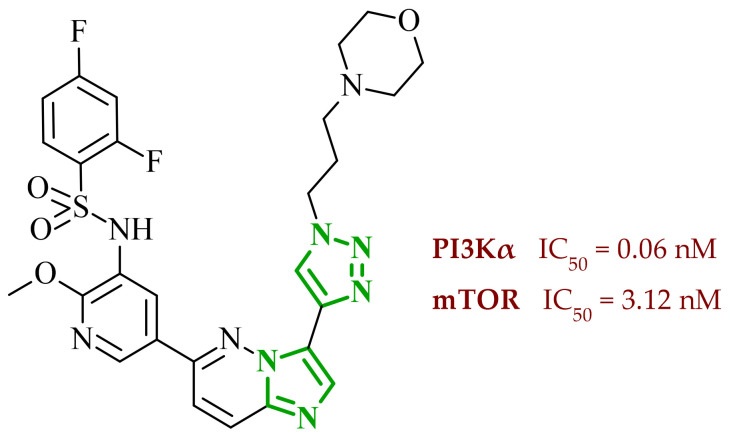
The structure of the most active imidazo[1,2-*b*]pyridazine derivative (**10**)-a potential PI3K and mTOR inhibitor [58].

Another group of researchers led by Li Ch. focused on a study aimed at the development of dual inhibitors based on imidazopyridazine scaffold targeting phosphoinositide 3-kinase (PI3K) and mammalian target of rapamycin (mTOR) [58]. The PI3K/Akt/mTOR signaling pathway involves numerous cellular processes related to cell growth, proliferation, and metabolism. For this reason, its deregulation is frequently observed in various cancers. PI3K enzymes belong to the family of lipid kinases, whereas mTOR is a serine/threonine kinase [59,60]. Feedback loops between these two kinases may limit the effectiveness of therapies targeting only one component of the pathway; therefore, simultaneous inhibition of both elements has attracted increasing interest as it may lead to improved therapeutic outcomes. Based on previous studies, the researchers selected a lead compound that exhibited unfavorable ADME parameters and decided to modify its structure. Starting from the imidazopyrazine core, SAR analysis revealed that both the heterocyclic moiety and the length and nature of linkers played a decisive role in PI3Kα inhibition. The initial modifications focused on variation in the linker introduced at the *C*-6 position, including amide, amide-triazole, and triazole motifs. Compounds bearing simple amide linkers showed moderate activity. Introduction of a triazole linker modestly improved potency. Further studies demonstrated that the imidazo[1,2-*b*]pyridazine system was more favorable for enzymatic activity. Replacement of the imidazo[1,2-*a*]pyrazine core with an imidazo[1,2-*b*]pyrazidine scaffold resulted in a significant improvement in PI3Ka inhibitory activity. In contrast, the introduction of bulky amide-triazole linkers led to substantial loss of activity. Additional SAR optimization demonstrated that direct attachment of the triazole moiety to the imidazo[1,2-*b*]pyridazine core was critical for maximizing potency. Subsequent optimization of the benzenesulfonamide moiety led to the synthesis of additional compounds. The variation in the aromatic substituent in this region was found to have a significant influence on PI3Kα inhibitory activity. In particular, halogenated phenyl derivatives exhibited enhanced potency, with compound **10** (42) (Figure 10) bearing a difluorophenyl group displaying the highest activity. The results of this study indicate that hydrophobic substituents in this region may be conducive to interactions with the affinity binding pocket of PI3K.

These modifications resulted in a balanced activity profile across the enzymatic and cellular assays, with PI3Kα inhibition (IC_50_ = 0.06 nM) and antiproliferative activity in HT-29 cells (IC_50_ = 0.09 μM). Molecular docking analysis revealed that compound **10** formed multiple hydrogen bonds within the active site of PI3Kα, including interactions with Lys802, Ser774, and Val851, as well as π-π interactions with Trp780. In contrast, the replacement of the morpholine ring with tetrahydropyrrole, piperidine, or *N*-methylpiperazine caused a reduction in activity. The substitution of halogen led to a slight decrease, while the removal of the methoxy group resulted in a dramatic loss of activity. Beyond PI3Kα, compound **10** demonstrated broad inhibition across PI3K isoforms and potent mTOR inhibition, matching or exceeding the activity of the reference inhibitor copanlisib. Molecular docking results indicated that in the case of mTOR, key interactions included hydrogen bonds with Thr2245 and Val2240, and π-π interactions with Trp2239, which contributed to stabilization of the ligand–enzyme complex and confirmed its strong inhibitory activity. Western blot analysis confirmed dose, and time-dependent suppression of Akt (protein kinase B) and p70S6K phosphorylation, consistent with effective inhibition of the PI3Kα/mTOR signaling pathway. Kinase selectivity profiling demonstrated that compound **10** exhibits a highly selective inhibitory profile, with over 99% inhibition observed primarily for PI3Kα isoforms, while most other kinases showed minimal inhibition (<35%), indicating a low risk of target activity. Pharmacokinetic evaluation in rats revealed that compound **10** is characterized by rapid plasma clearance and relatively short half-life, although in maintained acceptable oral bioavailability (33%), indicating partial suitability for systemic administration but also highlighting the need for further optimization. Importantly, in vivo studies demonstrated that compound **10** significantly inhibited tumor growth in HTC116 and HT-29 xenograft models, achieving tumor growth inhibition (TGI) values of up to 73.33%, comparable to copanlisib, while notably not inducing body weight loss in treated animals. Toxicological evaluation further confirmed a favorable safety profile, as compound **10** did not cause significant liver or kidney toxicity, in contrast to the previously reported lead compound, which exhibited hepatotoxic effects.

**Figure 11:**
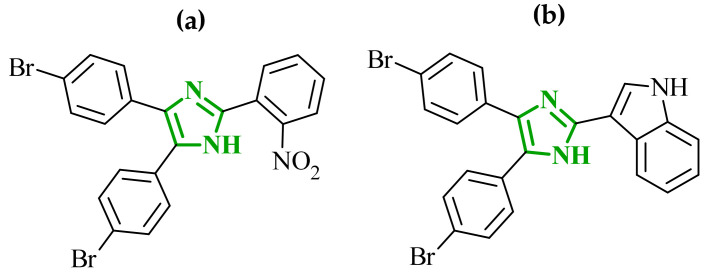
The structures of the most active 2,4,5-trisubstituted imidazole derivatives: (**a**) **11**; (**b**) **12**-potential EGFR and HER2 inhibitors [61].

In the search for additional multitarget inhibitors with potential anticancer potential, Guda et al. designed and synthesized a series of 2,4,5-trisubstituted imidazole derivatives targeting EGFR and human epidermal growth factor receptor 2 (HER2) kinases [61]. HER2 belongs to the ErbB family tyrosine kinase receptors. Despite lacking a known endogenous ligand, it plays a crucial role in cancer progression through indirect activation of many signaling pathways, including the PI3K/Akt/mTOR and MAPK/ERK pathways. Overexpression and/or amplification of the *HER2* gene has been observed in many types of cancer (including breast, stomach, and ovarian cancer). Excessive activation of HER2 leads to uncontrolled transmission of proliferative and anti-apoptotic signals [62]. It has been postulated that trisubstituted imidazole scaffolds exhibit a broad spectrum of biological activities, ranging from anticancer and antioxidant to antibacterial properties. Therefore, the authors focused on 2,4,5-trisubstituted imidazole scaffold, which is known for its broad spectrum of biological activities. The structural modifications involved the introduction of various aromatic and heteroaromatic substituents at the 2-, 4-, and 5-positions, bromophenyl, thiophene, indole, chromone, and nitro-substituted fragments. The antiproliferative activity of proposed compounds was evaluated using the MTT assay against IMR-32, A549 and HeLa cancer cell lines. Among the tested derivatives, compounds **11** (B11) and **12** (B16) (Figure 11) were the most active, with IC_50_ values ranging from 9.52 to 13.57 μM across the examined cell lines. Importantly, cytotoxicity evaluation against non-cancerous HEK293 cells revealed significantly higher IC_50_ values, indicating a favorable selectivity profile towards cancer cell lines. The comparison with the reference drug cisplatin highlights that although the tested compounds are less potent, they may offer improved safety and selectivity characteristics.

Although no direct enzymatic assays were performed, the combination of antiproliferative activity and favorable docking results suggests a possible mechanism through EGFR and HER2 inhibition. Molecular docking results were compared with data obtained for well-known reference inhibitors for EGFR, namely, gefitinib and lapatinib, and for HER2, such as afatinib and canertinib. Compound **12** exhibited favorable binding energies of −10.49 kcal/mol for EGFR and −10.12 kcal/mol for HER2. The analysis revealed the possibility of hydrogen bond formation between NH group of **12** and Thr766 in EGFR. Additional stabilization is governed by extensive hydrophobic interactions involving residues such as Val702, Ala719, Leu820, and Met742. In HER2 binding site stabilization is mainly driven by hydrophobic contacts with Leu785, Leu796 and Val734 amino acid residues. A similar binding profile was observed for compound **11**.

**Figure 12:**
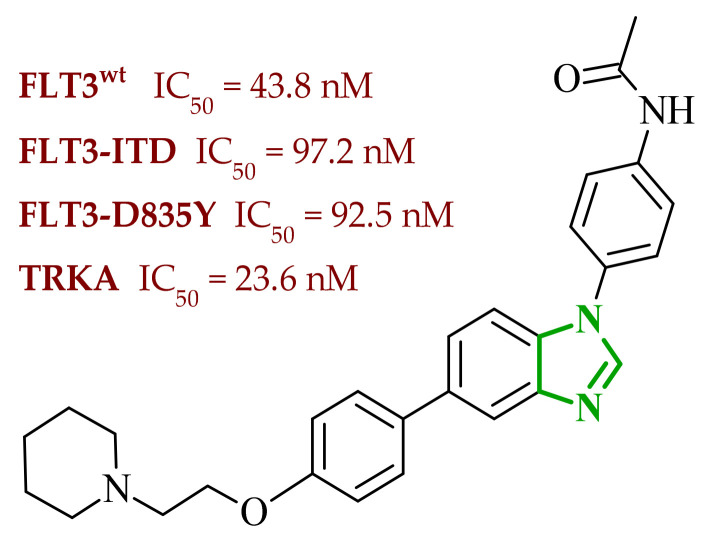
The structures of the most active benzimidazole derivative (**13**)-potential FLT3 and TRKA inhibitors [63].

A similar approach was proposed by Dokla and co-workers, who developed a novel kinase inhibitor based on a benzimidazole scaffold aimed at the treatment of acute myeloid leukemia (AML) [63]. The compound design was based on a scaffold-hopping strategy and structural simplification of the FLT3 inhibitor quizartinib. In the proposed concept, the imidazo[2,1-*b*]thiazole fragment present in quizartinib was replaced with a benzimidazole core, while the 5-(*tert*-butyl)isoxazole moiety was removed. These modifications were intended to yield a compound capable of inhibiting both wild-type FLT3 and the FLT3-ITD and FLT3-D835Y mutations, while simultaneously reducing activity toward the c-KIT kinase responsible for toxic side effects. Enzymatic assays demonstrated that the designed compound **13** (4ACP) (Figure 12) exhibits high activity against FLT3 kinase and its mutations associated with AML development. In vitro studies indicating that the compound is capable of inhibiting both the native enzyme and its clinically relevant mutants. Importantly, the compound also displayed significant activity toward TRKA kinase, allowing it to be classified as a potential dual FLT3/TRKA inhibitor. Studies performed on AML cell lines confirmed the selective antiproliferative activity of compound **13**. The strongest growth inhibition was observed in cell lines harboring the FLT3-ITD mutation. In contrast, cell lines lacking this mutation showed significantly lower sensitivity to the compound, indicating high selectivity toward FLT3-dependent cells. Developed compounds demonstrated a favorable preliminary safety profile. In vitro cytotoxicity assays confirmed that compound **13** did not exert significant toxic effects towards normal hepatocytes and cardiomyocytes, even at concentrations exceeding those required for antiproliferative activity in AML cells. Moreover, kinase selectivity profiling reveals off-target activity, particularly towards c-KIT, which is commonly associated with hematological toxicity and myelosuppression observed for clinically used FLT3 inhibitors.

Molecular docking analysis revealed that the benzimidazole ring of **13** occupies the hinge region of FLT3 kinase, forming a key hydrogen bond with the residue Cys694. Additionally, the acetamide fragment penetrates the allosteric channel, where it interacts with Asp829, while the piperidino-ethoxy substituent is oriented toward the solvent-exposed region and may form additional interactions with Cys695. A similar binding mode was observed in docking studies with TRKA kinase, where the benzimidazole core formed a hydrogen bond with Met592, stabilizing the ligand within the ATP-binding pocket. Structure–activity relationship analysis indicates that the benzimidazole core plays a crucial role in interactions with the hinge region of the kinase, enabling the formation of stable hydrogen bonds responsible for inhibitor activity. At the same time, the presence of appropriately positioned aromatic substituents and the piperidino-ethoxy fragment promotes stabilization of the ligand–enzyme complex through additional hydrophobic interactions and contacts with the solvent-exposed region of the enzyme. This combination of structural elements enabled the development of a compound with high activity against FLT3 and TKA while limiting activity toward kinases associated with adverse effects.

### 2.2. Pyrazole-Based Compounds

**Figure 13:**
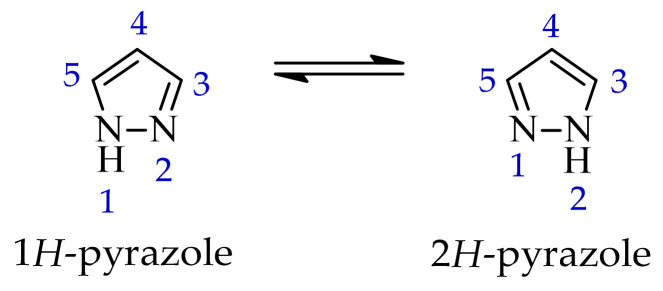
Two isomeric forms of pyrazoles [64].

**Figure 14:**
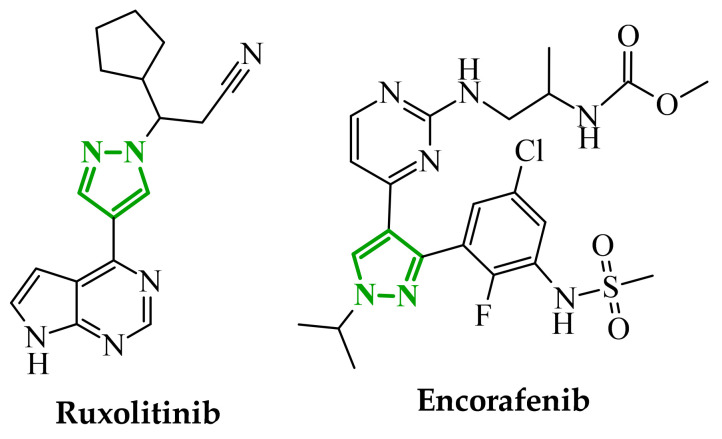
Selected examples of drugs incorporating the pyrazole fragment [69,70].

Another group of heterocyclic derivatives capable of acting on more than one molecular target comprises structures based on pyrazole derivatives. This structural element is significant in the context of numerous structures that function as drugs. Pyrazole exhibits degenerate prototropic tautomerism arising from proton transfer between adjacent nitrogen atoms of the aromatic ring. While 1*H*- and 2*H*-pyrazole aromatic tautomers may be formally defined, they are energetically comparable and undergo fast conversion in solution, and therefore cannot typically be distinguished under standard conditions (Figure 13) [64]. Pyrazole analogues have been shown to play an important role in the pharmacotherapy of diseases such as bacterial and viral infections and have anticancer and anti-inflammatory properties [65,66,67,68]. These derivatives represent a compelling subject for research, owing to their synthetic flexibility and pharmacological utility. An example of a pharmaceutical agent employed in the treatment of cancer that contains pyrazole in its structure is ruxolitinib (Figure 14). The substance under discussion is an oral JAK kinase inhibitor. The medication has been approved for the treatment of intermediate- and high-risk myelofibrosis and high-risk polycythaemia vera [69]. Another example is encorafenib. Moreover, it is an oral anticancer drug that is employed in the treatment of unresectable or metastatic melanoma (Figure 14) [70]. The pharmacokinetic properties of compounds containing pyrazole rings, such as encorafenib and binimetinib, are related to their metabolism in the liver. It has been demonstrated through metabolic stability studies that these compounds undergo enzymatic metabolism primarily via cytochrome P450 isoenzymes. This has a significant impact on their bioavailability and potential drug interactions. In addition, the results of the toxicological analysis suggest the potential for hepatotoxicity or nephrotoxicity [71]. Ruxolitinib is also metabolized primarily in the liver, mainly by the CYP3A4 isoenzyme and, to a lesser extent, by CYP2C9. Adverse effects associated with ruxolitinib include anaemia, thrombocytopenia, and an increased risk of infection, which are linked to its effects on the hematopoietic system [72].

**Figure 15:**
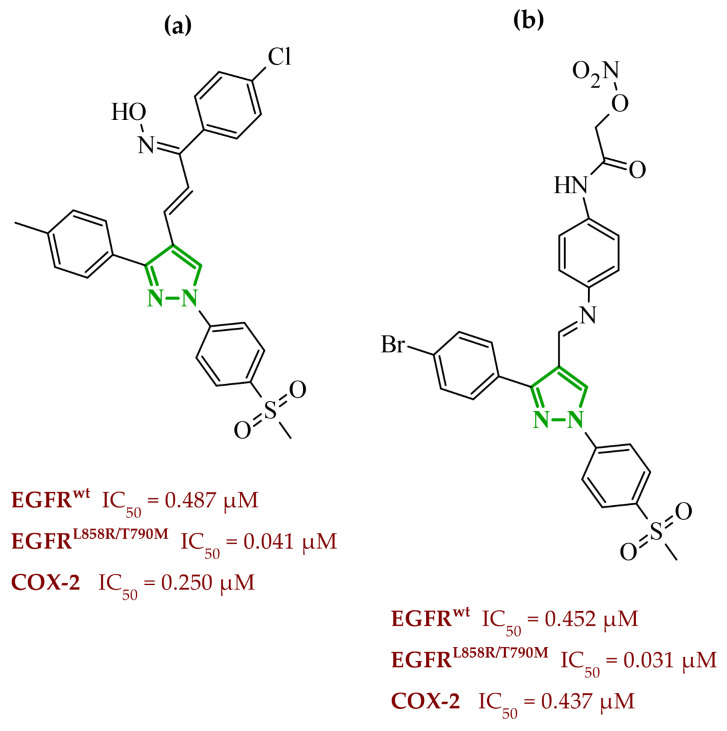
The structures of the most active pyrazole derivatives: (**a**) **14**; (**b**) **15**-potential EGFR and COX-2 inhibitors [73].

Consequently, in 2025, Fadaly and colleagues focused their research on the analysis of the properties of pyrazole derivatives as multitargeted inhibitors with potential anti-cancer activity [73]. The assumption was made that this group of compounds was active against both EGFR and COX-2. It was determined that diarylpyrazole scaffold is essential for the preferential inhibition of cyclooxygenase 2, as evidenced by the structure of celecoxib, as well-known selective COX-2 inhibitor. In this context, the pyrazole derivatives proposed by Fadaly, the most active of which **14** (14g) and **15** (17c) are shown in Figure 15, showed clear selectivity toward mutant EGFR over the wild-type receptor, while simultaneously exhibiting preferential COX-2 inhibition.

The half-maximal inhibitory concentration of the compounds against mutant epidermal growth factor receptor ranged from 0.031 to 0.080 µM, and against cyclooxygenase from 0.250 to 0.500 µM, indicating dual-target profile across the most active derivatives. The IC_50_ values against both molecular targets were comparable to the reference drugs osimertinib (0.037 µM) and celecoxib (0.416 µM). Detailed SAR analysis revealed that the nature of substitution on both the pyrazole *C*-3 phenyl ring and the terminal oxime/nitrate moiety critically governed potency and selectivity. Within the oxime series, the presence of the imino-chalcone fragment was essential for covalent interaction with Cys797 in mutant EGFR, acting as acceptor warhead. Importantly, halogenated derivatives demonstrated improved inhibition of mutant EGFR while maintaining weaker activity toward EGFR (wt), resulting in favorable selectivity indices. The substitution of the phenyl ring at position 3 of the pyrazole moiety significantly influenced COX-2 selectivity. Unsubstituted analogues exhibited generally lower COX-2 selectivity indices, whereas introduction of electron-donating or electron-withdrawing substituents enhanced COX-2 selectivity. It is observed, however, that excessive steric bulk bearing two *p*-bromo substituents led to reduced cytotoxic activity in resistant H1975 cells (IC_50_ = 51.33 µM), despite good inhibitory activity. This suggests that steric constraints may negatively affect cellular permeability or intracellular target engagement. In the nitrate series, the oxime functionality was replaced with a nitrate bearing imino-amido system, and the linker connecting the pharmacophoric fragments was shortened. These structural modifications preserved the ability of the compounds to form a covalent bond with Cys797 in mutant EGFR. Among the series, **15** emerged as the most balanced derivative, combining strong EGFR inhibition, good COX-2 selectivity, and potent cytotoxicity against H1975 cells. The presence of electron-withdrawing substituents on the pyrazole ring in **15** appeared to enhance both kinase selectivity and cellular potency. Additionally, compound **15**, induced cell-cycle arrest (G2/M in A549; G0/G1 in resistant H1975 cells) and significantly increased early and late apoptotic populations. Covalent docking studies supported experimental data. Compounds **14** and **15** form a covalent bond with Cys797, while the additional hydrogen-bonding interactions with Lys716 and Asp855, and hydrophobic contacts with Phe723, and Leu718 stabilize **15**/VEGFR complex. In silico ADMET profiling further revealed that a subset of oxime derivatives complied with Lipinski’s rule of five, displaying acceptable molecular weight, lipophilicity, and hydrogen bonding parameters. However, several derivatives, particularly nitrate-containing compounds, exceed optimal molecular weight and polar surface area value, which may negatively impact oral bioavailability. Additionally, the predicted low gastrointestinal absorption for most compounds and the lack of blood-brain barrier permeability suggest limited systemic exposure and reduced risk of central nervous system-related side effects. Finally, cytochrome P450 interaction analyses indicated that the compounds are likely to interact with CYP isoforms, which may influence metabolic stability and should be considered in further optimization studies.

**Figure 16:**
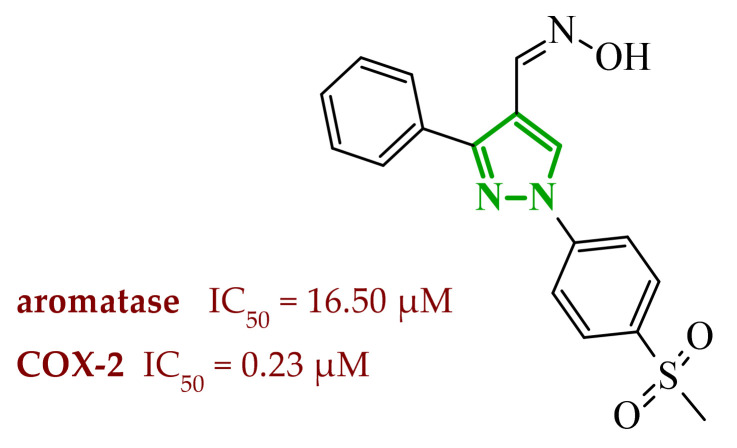
The structure of the most active pyrazole derivative combined with oxime (**16**)-a potential aromatase and COX-2 inhibitor [55].

In another study, Fadaly et al. synthesized two series of diarylpyrazole derivatives combining a selective COX-2 active pharmacophore with oxime or nitrate functionalities intended to act as nitric oxide donors [55]. These compounds were designed to simultaneously inhibit COX-2 and aromatase, aiming to interfere with both inflammatory signaling and estrogen biosynthesis pathways implicated in breast cancer progression [74]. Enzymatic evaluation revealed that several derivatives exhibited pronounced COX-2 inhibitory activity with high selectivity over COX-1. Compound **16** (11a) (Figure 16) emerged as the most potent selective inhibitor which are superior to celecoxib. In addition, compound **16** was tested in terms of their inhibitory activity against aromatase (CYP19A). Compound **16** showed activity similar to letrozole (IC_50_ = 15.60 µM), indicating its potential dual COX-2/aromatase inhibition profile. Molecular docking to the COX-2 binding site confirms the orientation of active compounds comparable to celecoxib. Cytotoxic activity was analyzed in the NCI 60 cancer cell line panel. Among all tested compounds, derivative **16** demonstrated the most promising antiproliferative profile, particularly against breast, melanoma, and ovarian cancer cell lines, with IC_50_ values of 3.12, 4.28, and 4.13 µM, comparable to doxorubicin. Selected compounds were evaluated for their selectivity toward normal cells using F180 fibroblasts, revealing that compound **16** exhibited a 4.82-fold selectivity towards cancer cells (MCF-7) over non-malignant cells. Compound **16** additionally induces cell cycle arrest at the G2/M phase, accompanied by a significant increase in apoptotic cell populations, as confirmed by both DNA flow cytometry and annexin-V assays. These findings provide evidence that the observed antiproliferative activity is associated with a defined mechanism of action rather than nonspecific cytotoxicity.

**Figure 17:**
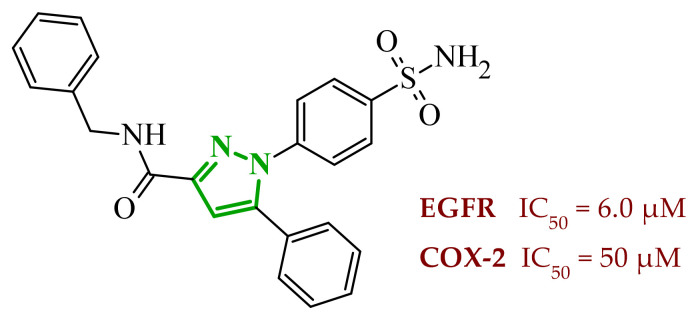
The structures of the most active diarylopyrazole derivatives (**17**)-potential EGFR and COX-2 inhibitors [56].

Soltan et al. designed and synthesized a series of 1,5-diarylpyrazole carboxyamide derivatives as potential dual EGFR/COX-2 inhibitors based on the celecoxib [56]. The classical diarylpyrazole scaffold bearing a *p*-sulfonamide group, known to be crucial for COX-2 selectivity, was preserved. The carboxylic acid moiety from the starting molecule was replaced with amide and hydroxamic acid residues to adjust the polarity, flexibility, and binding properties of the molecules. The antiproliferative screening against five cancer cell lines (HeLa, DLD-1, K562, SUIT2, and HepG2) revealed a clear structure-activity relationship. Among the carboxamide derivatives, benzylamine analogues displayed stronger activity than 4-benzylpiperidine and 2-methoxyaniline derivatives, which were weak or selective only toward SUIT2 cancer cell lines. The most potent benzylamine analogue was compound **17** (10a) (Figure 17). The most promising compound, **17**, exhibited also low cytotoxicity towards the normal human cell lines, resulting in favorable selectivity indices (SI = 5.9–11.5 depending on the cancer cell line). This indicates a clear therapeutic window and reduced off-target toxicity at biologically active concentrations. The evaluation of COX inhibition confirmed moderate COX-2 activity for several derivatives (IC_50_ = 10–50 µM). The EGFR enzymatic assay demonstrated that compound **17** inhibits the activity of EGFR, approaching that of the reference sorafenib (IC_50_ = 3.5 µM) and also showing moderate COX-2 inhibition. Molecular docking studies supported the experimental findings. In the EGFR active site, compound **17** can form hydrogen bonding interactions with Met769 and Asp831 via the carboxamide oxygen and sulfonamide NH group. The hydrophobic interactions in this case are stabilized via the diarylpyrazole core.

**Figure 18:**
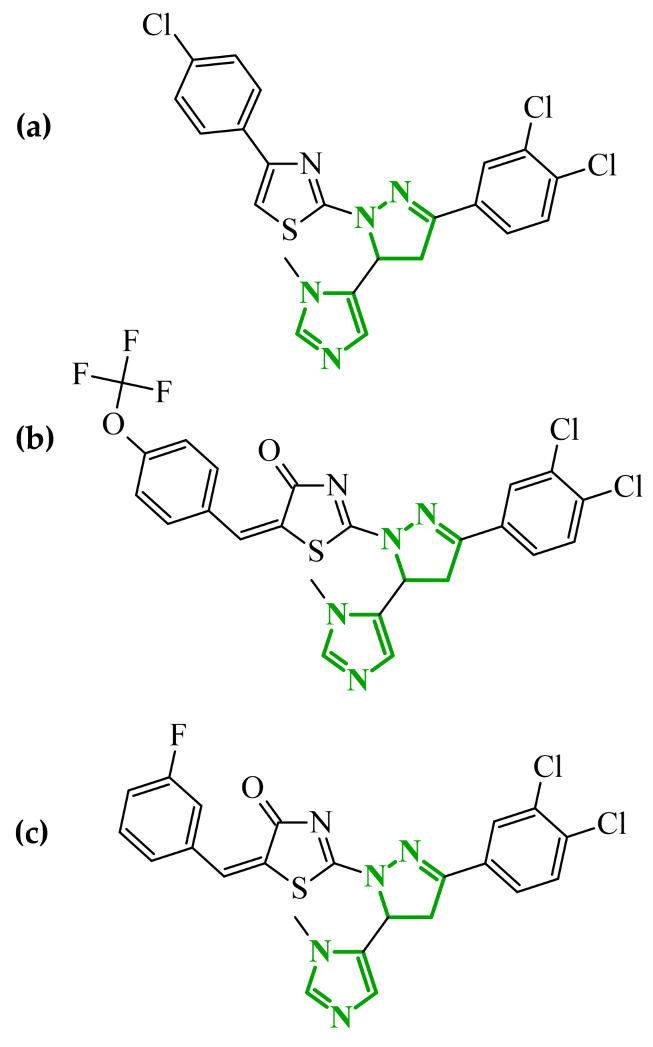
The structures of the most active pyrazole derivatives fused with an imidazole and thiazole ring: (**a**) **18**; (**b**) **19**; (**c**) **20**-potential ERK and RIPK3 inhibitors [75].

Boshta et al. designed and synthesized a series of 1,3,5-trisubstituted-1*H*-pyrazole derivatives as potential anticancer agents targeting extracellular signal-regulated (ERK) and receptor-interacting serine/threonine-protein 3 (RIPK3) kinases [75]. ERK is a kinase involved in the MAPK signaling pathway. Deregulation of this signaling cascade has been observed in various cancerous diseases. It plays a crucial role in cell survival and tumor promotion. The action of this factor depends on various factors, including cytokines, ultraviolet radiation, hypoxia, and pharmacological compounds. Inhibiting the ERK pathway has been shown to induce apoptosis, thereby regulating cancer cell growth and enhancing the efficacy of therapies [76]. Consequently, RIP3 kinase can regulate both apoptosis and necroptosis. There was a hypothesis that these kinases might disrupt cell membrane integrity or indirectly activate caspase 8 by phosphorylating specific proteins, which could lead to cell death [77]. The design strategy was based on a pyrazole core acting as a rigid bridge, enabling the introduction of diverse aromatic and heteroaromatic fragments at positions 1, 3, and 5. In particular, the incorporation of imidazole and thiazole rings was intended to enhance hydrogen-bonding capacity and improve interactions within kinase binding pockets. Cytotoxic activity was evaluated against MCF-7 and PC-3 cell lines. Among the synthesized compounds, derivatives **18** (6), **19** (10c), and **20** (10d) demonstrated the most pronounced effects (Figure 18).

Notably, compound **18**, bearing *p*-chlorophenyl substituent and a thiazole ring, exhibited the strongest activity against MCF-7 cells (IC_50_ = 3.9 µM), significantly exceeding doxorubicin (IC_50_ = 45.02 µM). The high potency of compound **18** may be influenced by the presence of the bulky and lipophilic moiety, which likely enhances hydrophobic interactions and improves cellular internalization. In contrast, compound, in which the thiazole ring was replaced by a 1,3-thiazete system, showed markedly reduced activity (IC_50_ = 35.5 µM in MCF-7), indicating that the heterocyclic thiazole ring is crucial for maintaining optimal planarity and interaction geometry. Further SAR analysis within the series revealed that substitution of the terminal benzylidene fragment strongly influenced cytotoxicity. For example, compound **19**, bearing a *p*-trifluoromethoxy group, and compound **20**, containing an *m*-fluorophenyl substituent, demonstrated moderate to good activity against PC-3 cells (IC_50_ = 28.6 µM and 57.6 µM, respectively), while derivatives lacking appropriate electronic and steric features were significantly less active. Cytotoxicity was additionally assessed against a normal epithelial cell line (RPE1), where the most active compound, particularly **20**, demonstrated a more favorable safety profile compared to other analogues, indicating reduced toxicity towards non-cancerous cells at biologically relevant concentrations. Moreover, flow cytometry studies revealed that selected derivatives induced cell cycle arrest in a phase-dependent manner (G0/G1 or S phase), further supporting a targeted biological effect rather than general toxicity. Further biological studies supported the proposed multitarget activity. In PC-3 cells, compounds **18** and **20** significantly reduced phosphorylated ERK levels, while simultaneously increasing pRIPK3 expression. Molecular docking studies confirmed that the most active derivatives adopted binding orientations similar to native ligands in both ERK2 and RIPK3 binding pockets. The pyrazole-imidazole core formed key hydrogen bonds with Met108 and Lys114 in ERK, while the dichlorophenyl moiety was engaged in hydrophobic interactions and π-sulfur contacts. In the binding center of RIPK3, compounds **19** and **20** formed stabilizing interactions with Met97 and Lys50. Interestingly, the *p*-trifluoromethoxy group in **19** established additional hydrogen and halogen bonds with Lys50, Asp160, and Glu60, which may explain its distinct binding orientation compared to **20**.

**Figure 19:**
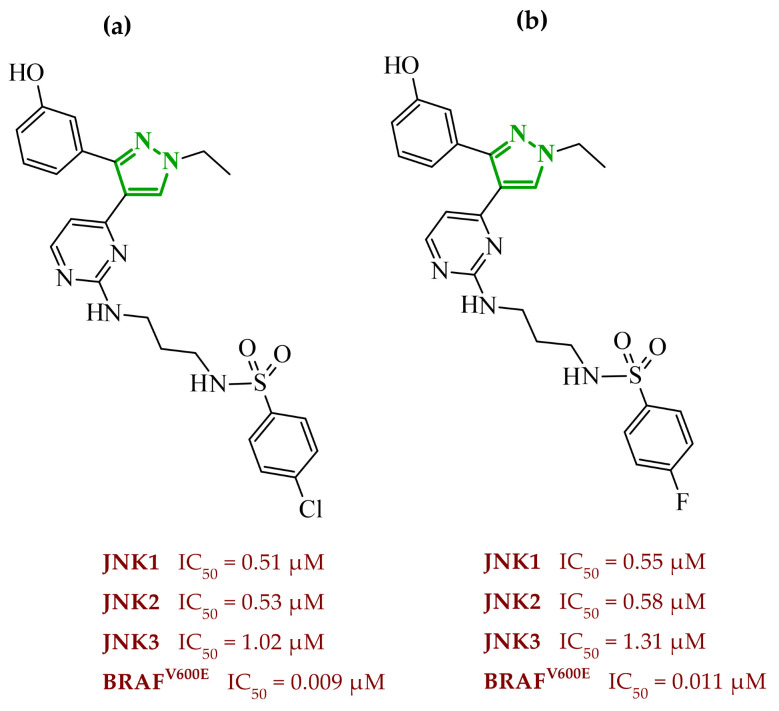
The structures of the most active pyrimidine-pyrazole derivatives: (**a**) **21**; (**b**) **22**-potential JNK and BRAF inhibitors [78].

In a recent study, Abdel-Maksoud et al. designed and synthesized two series of pyrimidinylpyrazole hybrids as dual anticancer and anti-inflammatory agents targeting BRAF^V600E^ and JNK (*C*-Jun *N*-terminal kinases) isoforms [78]. Excessive activation of kinases, including JNK kinases, has been demonstrated to lead to uncontrolled division and growth of cancer cells [79]. The activation of this kinase has been observed to occur under conditions of stress and/or in response to elevated cytokine concentrations. The design strategy was based on molecular hybridization of two pharmacophores, such as a pyrimidine-sulfonamide fragment derived from BRAF^V600E^ known inhibitors, and 3-phenylpyrazole scaffold associated with JNK inhibition. SAR evaluation revealed that hydroxyl-substituted derivatives were consistently more potent than their methoxy counterparts across all tested kinases. Demethylation of the 3-methoxy group significantly improved activity, particularly toward BRAF^V600E^. The cytotoxicity profile on leukemia and melanoma cell lines was consistent with the enzymatic profile. Hydroxyl derivatives **21** (14c) and **22** (14d) demonstrated submicromolar IC_50_ values, exceeding the activity of sorafenib in multiple models (Figure 19). Once again, halogen substitution is correlated with higher potency, whereas bulky or electron-donating substituents diminish activity. Western blot analysis confirmed that the most active compounds suppressed phosphorylation of MEK1/2 and ERK1/2, validating effective inhibition of the MAPK pathway downstream of BRAF^V600E^. Additionally, compound **22** induced G0/G1 cell cycle arrest and significantly reduced melanoma cell migration.

**Figure 20:**
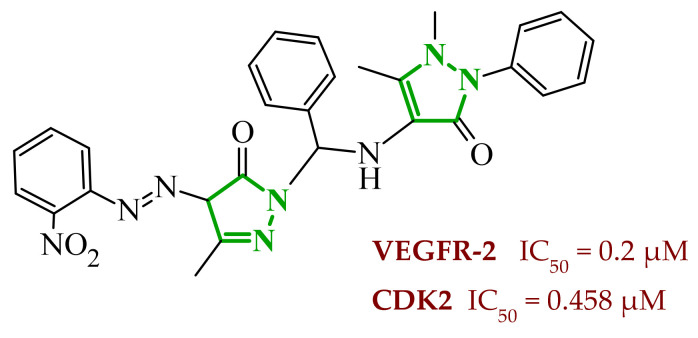
The structure of the most active pyrazolone derivative (**23**)-a potential VEGFR-2 and CDK2 inhibitor [80].

In another study, Salem and coworkers reported a new series of pyrazolone derivatives designed as dual VEGFR-2 and CDK2 inhibitors with anticancer activity [80]. They performed molecular hybridization of a 3-methyl-4-arylazo-pyrazol-5-ol core with several secondary amines introduced via Mannich-type modification. This approach enabled modulation of both electronic properties of the aryl fragment and steric as well as physicochemical characteristics of the terminal amine moiety, which proved crucial for dual kinase inhibition. They performed enzymatic assays, which revealed clear structure-activity relationships within the series. Among all derivatives, compound **23** (6b) demonstrated the most pronounced dual inhibitory activity against VEGFR-2 and CDK2 (Figure 20). Compound **23** exhibited the strongest VEGFR-2 inhibition and possessed substantial CDK2 activity. The analysis indicated that the nature of the arylazo substituent at position 4 of the pyrazolone ring significantly influenced kinase inhibition. Derivatives bearing electron-withdrawing substituents on the aromatic ring exhibited improved VEGFR-2 potency compared to unsubstituted analogs. In contrast, compounds with fewer electronically activated aryl groups showed diminished activity, highlighting the importance of optimal electronic distribution for effective target engagement. The cytotoxic potential of the synthesized compounds was evaluated in HepG2 liver cancer cells. The most active enzymatic inhibitors also demonstrated the strongest antiproliferative effects, confirming consistency between biochemical and cellular data. In particular, compound **23** exhibited pronounced growth inhibition and induced apoptosis accompanied by S phase cell cycle arrest, supporting its dual mechanism of action at both VEGFR-2 and CDK2 levels.

According to molecular docking, the most potent derivatives formed key hydrogen bonds with Asp1046 within the VEGFR-2 binding site, a residue critical for kinase inhibition. In the case of CDK2, the interactions with Lys89 were present. Compound **23** displayed the most favorable binding profile, characterized by strong binding interactions and optimal orientation within both kinase active sites. In addition to biological evaluation, physicochemical and pharmacokinetic properties indicated that the most active compound complies with Lipinski’s rule of five, with acceptable molecular weight, hydrogen bond donor/acceptor counts, and lipophilicity, suggesting favorable drug-like properties.

**Figure 21:**
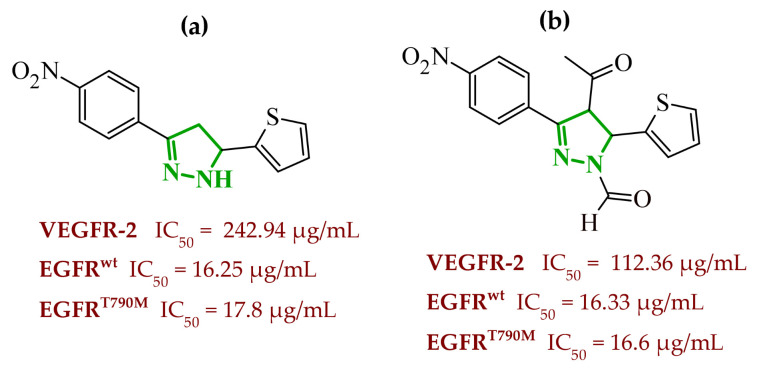
The structures of the most active pyrazole-thiophene hybrids: (**a**) **24**; (**b**) **25**-potential EGFR and VEGFR-2 inhibitors [81].

Sallam and co-workers designed a novel series of pyrazole-thiophene hybrids as multitarget inhibitors of wild-type EGFR, mutant EGFR (T790M), and VEGFR-2, aiming to simultaneously suppress tumor proliferation and angiogenesis [81]. In this case, rational design was based on the combination of two pharmacophologically relevant structural motifs. The design strategy involved combining an electron-rich pyrazole core, which is commonly found in kinase-targeting agents, with a lipophilic thiophene group to enhance receptor binding and improve cellular penetration. The structure-activity relationship analysis revealed several important trends. The unsubstituted *N*-1 position on the pyrazole ring, as in compound **24** (2), was associated with the highest cytotoxic and EGFR inhibitory activity. However, further consideration of the structure and modulation of substituents on the pyrazole ring revealed that the introduction of a formyl group at the *N*-1 position and an additional acetyl group (compound **25** (14)) did not result in a decrease in activity. It can be concluded from these observations that the double substitution of the ring does not so much result in the elimination of activity as the modulation thereof (Figure 21).

Likewise, the aromatic acid–derived substituents preserved reasonable cytotoxicity and enhanced VEGFR-2 inhibition. In contrast, elongation of the *N*-1 substituent with more polar, bulkier groups (e.g., 4-oxobutanoic acid) reduced cytotoxic potency, likely due to the increased steric demand and altered physicochemical balance. Similarly, the expansion of the pyrazole ring to bulkier heterocyclic systems such as benzodiazepines, led to a noticeable reduction in cytotoxic activity, indicating that increased ring size is unfavorable for maintaining biological effect. The substitution at the *C*-4 position of the pyrazole ring also modulated activity. Comparison of compound **25** with an acetyl group in *C*-4 position and its non-acetylated derivative suggested that small electron-withdrawing fragments at *C*-4 may enhance kinase interactions. Cytotoxic activity of designed compounds was evaluated against breast cancer (MCF-7) and hepatocellular carcinoma (HepG2) cell lines. Among the synthesized derivatives, compound **24** emerged as the most potent analogue, displaying IC_50_ values of 6.57 µM for MCF-7 and 8.86 µM for HepG2, comparable to doxorubicin (4.17 µM and 4.50 µM, respectively), erlotinib (8.20 µM and 7.73 µM, respectively), and sorafenib (7.26 µM and 9.18 µM, respectively). Compound **25** demonstrated moderate but balanced activity toward both cell lines (12.94 µM and 19.59 µM, respectively). Enzymatic assays confirmed the multitarget profile. Compound **24** exhibited the strongest inhibition of wild-type EGFR, close to compound **25**. Compounds **24** and **25** were also potent inhibitors of T790M mutant EGFR, but were less active against VEGFR-2. Cell-based investigations strengthen the biological profile of the most active derivatives. Data revealed that treatment of MCF-7 cells with compound **25** induced pronounced G0/G1 cell cycle arrest (about 74% vs. 55% in the control group) significantly increased apoptosis (about 26%). Molecular docking analysis proved that compound **24** can form hydrogen bonding interactions with Met793 and π-contacts with Leu718 within the wild-type EGFR binding pocket. Compound **25** establishes hydrogen bonds with Lys745 and Met790 in mutant EGFR, like the reference inhibitor.

**Figure 22:**
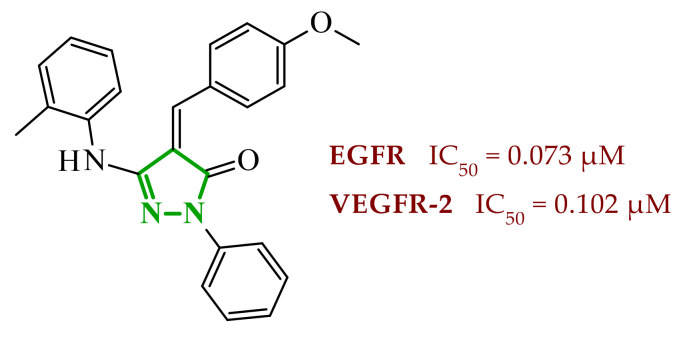
The structure of the most active tri-substituted pyrazolone derivative (**26**)-a potential VEGFR-2 and EGFR inhibitor [82].

In a recent study, Alhamaky and co-workers designed and synthesized a series of di- and trisubstituted pyrazolone derivatives as well as pyrazolopyridine analogues as potential dual inhibitors of EGFR and VEGFR-2 [82]. The series was designed based on the assumption that the planar pyrazole scaffold could effectively occupy the ATP-binding cavity of EGFR, while *N*-phenylpyrazole can simultaneously engage the hinge region of VEGFR-2. Additionally, the carbonyl group at position 3 of the pyrazole ring serves as a hydrogen bond acceptor. On the other hand, the presence of hydrogen bond donors at position 5 (e.g., -NH) is crucial for the EGFR inhibition. Enzymatic assays confirmed that compounds bearing unsubstituted or moderately substituted phenyl rings displayed superior inhibitory activity compared with bulkier or strongly electron-withdrawing analogues. Bulky substituents result in weaker kinase inhibition. Among the investigated derivatives, compound **26** (3f) emerged as the most potent dual inhibitor against EGFR and VEGFR-2 (Figure 22).

The selectivity index indicated a favorable safety margin toward healthy cells (SI 20.84). This favorable selectivity profile is further supported by comparative analysis with the reference drug erlotinib, for which a significantly lower selectivity index was reported (SI = 3.42). In contrast, compounds lacking the hydrogen bond donor at position 5 showed weaker activity. Additionally, cell-based studies showed that the most active derivatives inhibited the proliferation of cancer cell lines and induced cell cycle arrest at the G1/S transition, accompanied by apoptosis induction. This effect was particularly evident for compound **26**, where a pronounced increase in both early and late apoptotic populations was observed, together with modulation of key apoptotic markers, including increased Bax and caspase-3 levels and decreased Bcl-2 expression. They also observed that in the EGFR binding active site, compound **26** forms key hydrogen bonds with Gly772 and Met769. Within the VEGFR-2, the pyrazole core enabled formation of a crucial hydrogen bond with Asp1046, a residue located in the activation loop, while hydrophobic phenyl substituents occupy adjacent lipophilic pockets.

**Figure 23:**
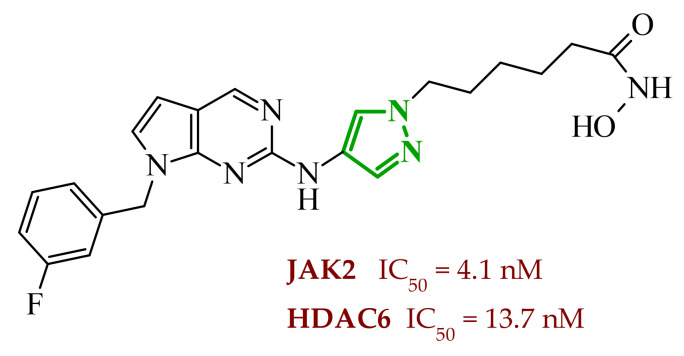
The structure of the most active pyrrolo[2,3-*d*]pyrimidine (**27**)-a potential JAK and HDAC inhibitor [83].

Liang et al. developed pyrrolo[2,3-*d*]pyrimidine derivatives containing pyrazole ring as dual JAK/HDAC inhibitors. The compounds also contained an aminopyrimidine moiety that binds to JAK and a hydroxamic acid moiety that binds zinc and blocks HDAC [83]. HDACs (histone deacetylases) regulate the acetylation of various substrates and thus control the expression level of many genes, including oncogenes and genes involved in apoptosis [84]. To date, several HDAC inhibitors have been approved by the FDA, including belinostat [85]. Despite their clinical utility, these agents still present limitations such as adverse effects, pharmacokinetic constraints, and limited efficacy in some cancers, highlighting the need for the development of improved HDAC inhibitors. Using multifunctional inhibitors could solve this problem. Structure-activity relationship analysis in this project focused on systematic modification of the linker length and the aromatic region. An aliphatic spacer consisting of five to six methylene fragments was found to be optimal, maintaining high potency toward both enzymes. This linker length influences correct positioning of the hydroxamic moiety within the HDAC catalytic channel while simultaneously preserving interactions with the ATP-binding pocket of JAK. Introduction of substituents at the *para* position of the phenyl fragment typically led to a reduction in JAK inhibitory potency, although HDAC6 inhibition was preserved. In contrast, halogen substitution at the *ortho* or *meta* positions on the phenyl or benzyl fragments was well accommodated and often associated with potent dual inhibition in the low-nanomolar range. Conversely, the introduction of a methoxy group at the *meta* position reduced JAK inhibitory activity, even though HDAC6 inhibition frequently remained substantial. Likewise, the modification of the scaffold toward more flexible benzyl or phenethyl variants tended to maintain HDAC6 potency but resulted in diminished JAK inhibition. Within the series, compound **27** (15g) (3-fluorophenyl derivative) emerged as the most balanced dual agent, inhibiting JAK2 and HDAC6 (Figure 23).

In triple-negative breast cancer cells (MDA-MB-231), compound **27** and three others from this series increase levels of acetyl-α-tubulin and acetyl-H3K9/14, together with attenuation of JAK-STAT pathway signaling. An isomer of compound **27**, 2-fluorophenyl derivative, induced apoptosis in MDA-MB-321 cells in a dose-dependent manner (approximately 62% at 5 µM and 75% at 10 µM). Furthermore, in a mouse xenograft model, the same compound demonstrated measurable antitumor efficacy, achieving tumor growth inhibition of approximately 37% at 30 mg/kg/day and 49% at 100 mg/kg/day. Molecular docking study revealed that the aminopyrimidine motif reproduced key hinge region hydrogen bonds in JAK2, while a fragment of hydroxamic acid provided the canonical Zn^2+^ chelating interactions in HDAC6 and important hydrogen bonding contacts with Tyr745, His573, and His574.

**Figure 24:**
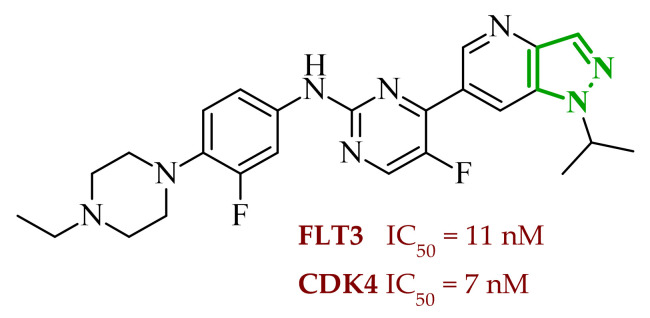
The structure of the most active (**28**)-a potential FLT3 and CDK4 inhibitor [86].

A similar strategy was reported by Li and co-workers, who designed a series of 6-(pyrimidin-4-yl)-1*H*-pyrazolo[4,3-*b*]pyridine derivatives as potential dual inhibitors of FLT3 and CDK4 kinases, both of which play important roles in the pathogenesis of acute myeloid leukemia [86]. The design strategy was based on a previously reported JAK2/FLT3 inhibitor, which also exhibited activity toward CDK4. Based on earlier SAR analyses, the authors modified the molecular scaffold by replacing the pyrazole fragment with other heterocycles and by altering substituents on the aromatic ring, which led to the synthesis of a series of new compounds with potential activity against both kinases. Preliminary enzymatic activity studies revealed significant structure–activity relationships within the analyzed compound series. In the first stage, the effect of replacing the benzene ring with nitrogen-containing heterocycles was evaluated. However, the obtained compounds exhibited markedly reduced activity against JAK2, CDK4, and FLT3 kinases compared with the reference compound. Furthermore, the introduction of substituents at position 3 of the aromatic ring led to decreased kinase selectivity, indicating the crucial role of the aromatic core structure in enzyme binding. Subsequent modifications involved the introduction of substituents at the *C*-5 position of the pyrazole ring. These results confirmed the validity of the adopted strategy for designing inhibitors targeting a dual molecular target. Further structural modifications involving the incorporation of more complex bicyclic systems led to compounds with increased activity toward CDK4. The most promising results were obtained for compound **28** (23k) (Figure 24), that showed the highest enzymatic activity in kinase assays against FLT3 and CDK4. Moreover, cell-based studies demonstrated strong antiproliferative activity against leukemia MV4-11 (FLT3-ITD) cells with an IC_50_ value of approximately 70 nM, whereas FLT3-independent cell lines showed considerably lower sensitivity to the compound. Molecular docking studies demonstrated that compound **28** forms a stable complex with both CDK4 and FLT3 kinases. In the case of CDK4, two nitrogen atoms of the 2-aminopyrimidine fragment form hydrogen bonds with the residue Val96 in the hinge region of the enzyme. Additionally, the bicyclic system of the molecule is stabilized by π-π interactions with neighboring amino acid residues such as Val20, Lys35, and Ala157. In the complex with FLT3 kinase, formation of a salt bridge between the residue Glu661 and the piperazine fragment of the ligand was observed, along with aromatic interactions between the phenyl ring and residues Phe691 and Phe830, which stabilize the ligand within the ATP-binding pocket.

Structure–activity relationship (SAR) analysis demonstrated that the biological activity of the studied compounds strongly depended on the nature of substituents within the bicyclic system and the aromatic ring. Introduction of strongly electron-withdrawing substituents such as CF_3_ led to a significant decrease in activity against FLT3 and CDK4 kinases. A similar effect was observed for substituents causing substantial steric hindrance, which altered the molecular conformation and limited the ability to interact with amino acid residues in the enzyme active site. In contrast, the presence of moderately hydrophobic substituents favored stabilization of the ligand–enzyme complex and resulted in increased inhibitory activity. The obtained results indicate that appropriately designed pyrazolo[4,3-b]pyridine derivatives may act as effective dual FLT3/CDK4 inhibitors. In particular, compound **28** demonstrated strong anticancer activity in both enzymatic and cellular studies, and in an MV4-11 xenograft model, it inhibited tumor growth by 67% at a dose of 200 mg/kg, confirming its potential as a candidate for further anticancer drug development. At the cellular level, compound **28** was shown to induce cell cycle arrest in the G1 phase, consistent with CDK4 inhibition, and simultaneously promote apoptosis in a concentration-dependent manner, particularly in FLT3-driven cells.

### 2.3. Triazole-Based Compounds

Triazoles are a prominent group of five-membered heterocycles that contain three nitrogen atoms. The presence of three nitrogen atoms instead of two in a five-membered heterocyclic system (as in imidazole or pyrazole scaffolds) decreases the basicity and increases the acidity of the aromatic ring [87]. The molecules can be divided into two classes based on the position of nitrogen atoms in the aromatic ring–1,2,3-triazoles and 1,2,4-triazoles. Triazole motifs are present in various drugs due to their ability to act as bioisosteres of chemical compounds such as amides, esters, carboxylic acids, and other heterocyclic systems. The triazole moiety can interact with enzymes or receptors via non-covalent interactions (e.g., hydrogen bond formation, dipole-dipole or ion-dipole interactions, cation-π interactions, π-π stacking, hydrophobic interactions, and van der Waals forces) [88,89]. Additionally, the polar nature of this scaffold can increase solubility, while its resistance to metabolic degradation may enhance bioavailability. Both traits improve the pharmacological profile of potential drugs [87,90].

#### 2.3.1. 1,2,3-Triazole Derivatives

**Figure 25:**
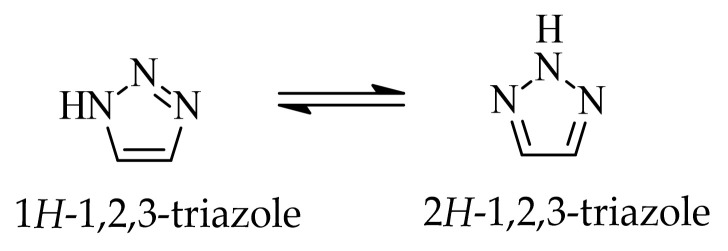
Two isomeric forms of aromatic 1,2,3-triazoles [91].

**Figure 26:**
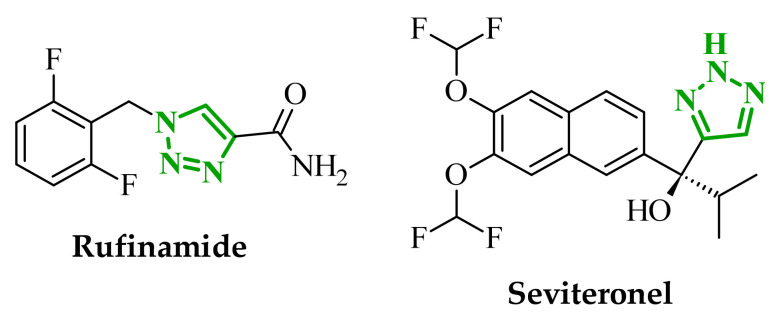
Selected examples of drugs incorporating the 1,2,3-triazole fragment (two isomeric forms included) [103,104].

1,2,3-Triazoles may occur in two aromatic isomeric forms, namely 1*H*-1,2,3-triazole and 2*H*-1,2,3-triazole (Figure 25) [91]. However, 1*H*-1,2,3-triazole derivatives are attracting more interest in the search for potential drugs. In addition to the fact that 1*H*-1,2,3-triazole scaffolds may act as pharmacophores themselves, they often also serve as linkers between two different pharmacophores [92]. This isomeric structure can be obtained through the click reaction based on the copper-catalyzed azide-alkyne cycloaddition [93,94]. 1,2,3-Triazoles possess a wide range of pharmacological properties, including, for instance, anticancer [95,96,97], antibacterial [98], antifungal [99], antiviral [100], antitubercular [101], and anti-obesity [102] activities. Drugs with the 1,2,3-triazole scaffold are already present on the market. One example is rufinamide, an antiepileptic drug for adjunctive treatment of seizures associated with Lennox–Gastaut syndrome [103]. Seviteronel, containing the 1,2,3-triazole moiety, is currently being evaluated in clinical trials for estrogen receptor-positive or triple negative breast cancer (Figure 26) [104]. Rufinamide demonstrates excellent oral bioavailability, with its primary metabolic pathway involving the hydrolysis of the carboxyamide group to an inactive metabolite, a carboxylic acid. This process is not dependent on cytochrome P450 enzymes, and the resulting metabolites are predominantly excreted in the urine [105]. In contrast, seviteronel is categorized as a CYP17A1 inhibitor. Its heterocyclic nitrogen ring facilitates coordination with the iron atom at the enzyme’s heme centre. This interaction stabilizes the inhibitor–enzyme complex and increases the compound’s affinity for the active site, thereby influencing its biological activity [106]. Compounds containing a triazole ring may therefore exhibit varied pharmacokinetic properties; however, their metabolism and elimination depend largely on the presence of specific functional groups and interactions with metabolic enzymes.

**Figure 27:**
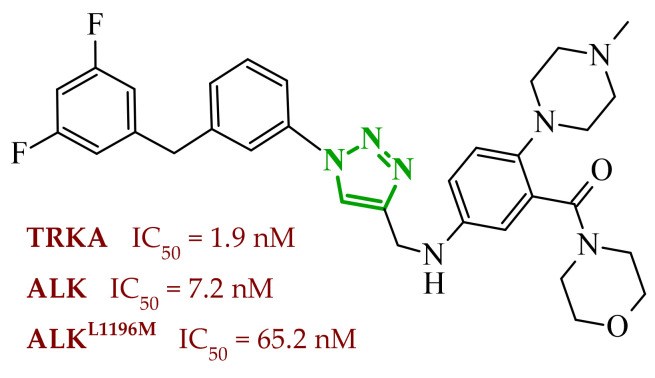
The structure of the most active phenyl triazole derivative (**29**)-a potential ALK and TRKA inhibitor [107].

Among the potential dual-inhibitors, Cao et al. synthesized a series of phenyl triazoles as modifications of entrectinib to act as regulators of oncogenic drivers-ALK and TRKA [107]. Replacing the aminoindazole moiety with a phenyltriazole makes the compounds more flexible. Compound **29** (13a) turned out to be the optimal hit (Figure 27). Derivative **29** exhibited inhibitory activity against TRKA, ALK and ALK^L1196M^, which were comparable to or much better than those of entrectinib. Compound **29** showed potent cytotoxic activity (IC_50_ = 0.079–0.138 μM) against TRK-addicted and ALK-addicted cell lines. Additionally, compound **29** was found to inhibit the migration and colony formation of TRKA-positive KM12 cells in a dose-dependent manner, and induce apoptosis of KM12 cells (comparable to entrectinib at the same dose). Compound **29** was also tested on human normal cell lines (HELF and QSG-7701) and was found to be safe. According to in silico studies, the phenyl triazole scaffold was anchored to the kinase hinge region through a hydrogen bond between the nitrogen atom of the triazole and Met592 (TRKA) or Met1199 (ALK). The 3,5-difluorophenyl substituent formed halogen interactions with amino acid residues within the DFG motif, while at the opposite end, the morpholine moiety established a new hydrogen bond with the sugar pocket. The *N*-methylpiperazine residue located in the solvent region proved to be more favorable than piperidine or hydroxy/methylpiperidine substituents. At the same time, the morpholine in the sugar pocket gave better results than piperidine, pyrrolidine or *N*,*N*-dimethylamine substituents.

**Figure 28:**
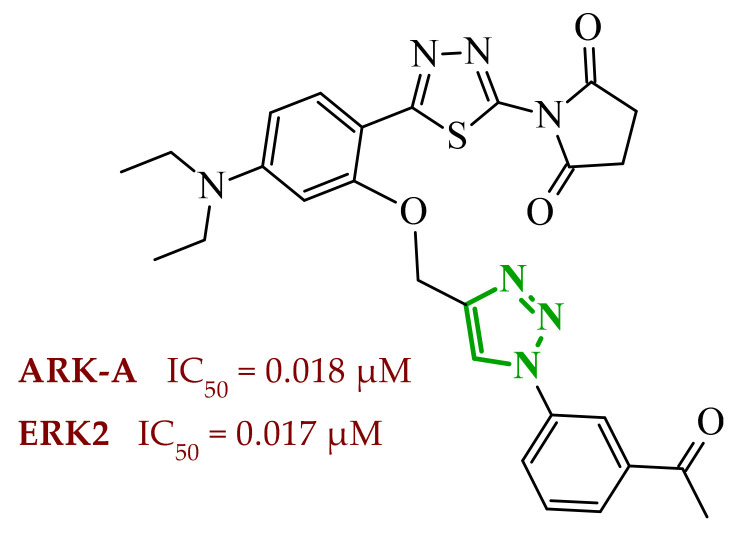
The structure of the most active 1,2,3-triazole-thiadiazole hybrid (**30**)-a potential ARK-A and ERK2 inhibitor [108].

Bontha et al. synthesized and evaluated novel 1,2,3-triazole-thiadiazole hybrids with different substituents in the phenyl group attached to the triazole moiety [108]. The lead structure was **30** (9j) (Figure 28). Compound **30** showed dual inhibition potential against ARK-A (also AurA; Aurora kinase A) and ERK2 proteins. Compound **30** displayed satisfactory anticancer activity against KYSE-450 cells with IC_50_ of 23.74 µM and against MIA PaCa-2 cells with IC_50_ of 20.62 µM. It has also been shown that derivative **30** had a prominent impact on the cellular morphology of KYSE-450 and MIA PaCa-2 cells. According to molecular docking simulations, compound **30** displayed docking scores of −10.4 kcal/mol toward ARK-A and −9.3 kcal/mol toward ERK2. The greatest number of interactions in binding to ARK-A and ERK2 was observed for the substituted phenyl group attached to the triazole moiety, as well as for the 1,3,4-thiadiazol-2-yl-pyrrolidine-2,5-dione part. SAR studies showed that the observed anticancer activity can be attributed to the presence of an acetyl group at the *meta* position of the phenyl attached to the triazole moiety. Derivatives bearing substituents such as halogen atoms, nitro, methoxy, or methyl groups at the *ortho* or *para* positions did not act as dual inhibitors.

**Figure 29:**
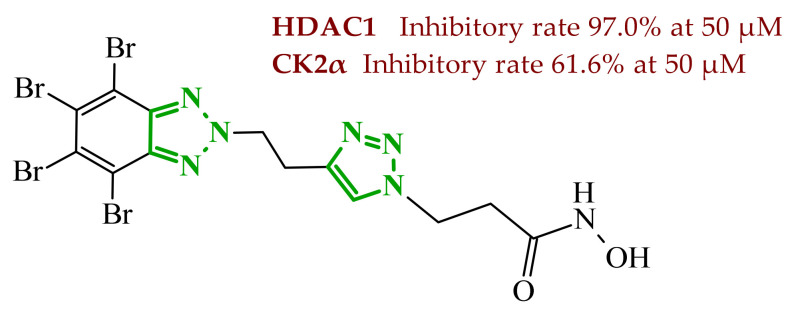
The structure of the most active TBB-hydroxamate hybrids linked by a triazole scaffold (**31**)-potential HDAC and CK2 inhibitors [109].

Purwin et al. obtained a series of hybrid compounds as inhibitors of HDAC and kinase CK2 [109]. Reducing HDAC activity can reverse epigenetic alteration connected with cancer, whereas CK2 inhibition aims at disruption of tumor maintenance and progression [109,110]. The synthesis of new series based on the structures of pharmacophores targeting the binding sites of these proteins—a hydroxamate that chelates the zinc ion in the active site of HDAC and a 4,5,6,7-tetrabromobenzotriazole (TBB) moiety that interacts with the ATP binding site in CK2 (and also acts as the cap group for HDAC). These two fragments were connected by a 1,2,3-triazole motif with CH_2_ groups of various lengths on both sides. The most optimal results were obtained for compound **31** (5e) (Figure 29). Compound **31** also demonstrated high activity against Jurkat cells (LC_50_ = 2.87 μM) and MCF-7 cells (LC_50_ = 4.26 μM) with selectivity of action for tumor cells. The selected compound was shown to induce apoptosis in leukemia cells in a concentration-dependent manner. In silico studies confirmed that the hydroxamate motif of this compound coordinated with the catalytic zinc ion of HDAC via two oxygen atoms, creating several hydrogen-bond interactions. The presence of methylene groups between the hydroxamate and the triazole can establish van der Waals interactions, which make the TBB cap stick out of the binding tunnel. Docking of the potential dual inhibitor into CK2 revealed that the TBB moiety occupies the hydrophobic ATP-binding pocket, allowing it to establish a halogen bond and strong van der Waals contacts. It was also found that the hydroxamate residue was suitable for forming a hydrogen bond within the phosphate-binding region of CK2. The number of methylene groups between the triazole linker and TBB, as well as between the triazole and the hydroxymate, was crucial for the activity of the compounds. Two methylene groups on both sides of the triazole provided the optimal length and flexibility for efficient binding to HDAC and CK2. One methylene group between the triazole motif and TBB was insufficient. Similarly, three and four methylene groups or an aromatic ring between the triazole and the hydroxymate were also inadequate. The *N*-1 substituted isomer of compound **31** also exhibited promising results as the dual inhibitor (90.0% of inhibition HDAC1 and 79.0% of inhibition CK2α at 50 μM) but showed a lack of selectivity against cancer cells.

**Figure 30:**
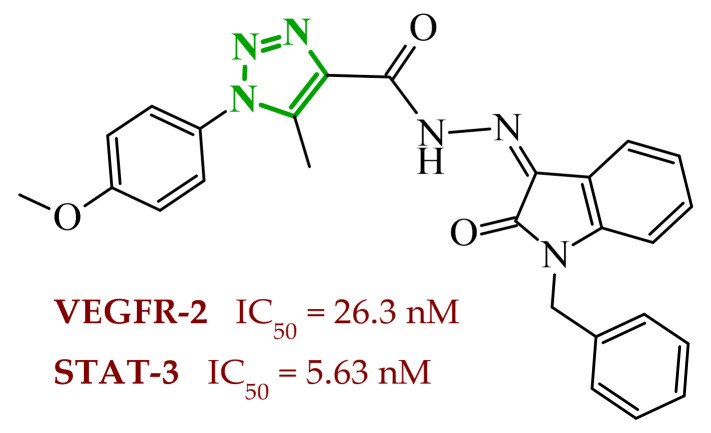
The structure of the most active isatin-grafted phenyl-1,2,3-triazole hybrid (**32**)-a potential VEGFR-2 and STAT-3 inhibitor [111].

Elsebaie et al. synthesized a series of isatin-grafted phenyl-1,2,3-triazole hybrids [111]. The core of the compounds consisted of three aromatic rings: an isatin moiety connected via a linker to a methyl-triazole scaffold followed by a hydrophobic phenyl ring. Among all modifications in the isatin region, compound **32** (9f) proved to be the most active (Figure 30). It occurred as a *Z*-isomer with an intramolecular hydrogen bond between the NH proton and the carbonyl group of the indolinone ring. The hybrid exhibited high inhibitory potency against VEGFR-2 (sunitinib IC_50_ = 30.70 nM) and signal transducer and activator of transcription (STAT-3). The MTT assay revealed that compound **32** exhibited IC_50_ values of 0.13 μM against PANC1 cell lines and 0.10 μM against PC3 cell lines, which were much lower than those of sunitinib or doxorubicin. Additionally, compound **32** exhibited higher selectivity for cancer cells than for normal cells (WPMY-1 lines). Derivative **32** also showed upregulation of apoptotic markers (caspase-8, caspase-9 and Bax) and downregulation of anti-apoptotic protein Bcl-2. In silico studies showed that compound **32** was located in the binding site of VEGFR-2 through the formation of hydrogen bonds involving: the methoxy group, the nitrogen atom from the triazole, the hydrazide nitrogen, and the carbonyl oxygen from the isatin scaffold. Hydrogen bonds were also observed between the hydrazide nitrogen or carbonyl oxygen from the isatin and amino acid residues in STAT-3. Furthermore, the isatin and the phenyl near the triazole motif stabilized the orientation of the compounds through hydrophobic interactions within both enzymes. For compound **32**, ADMET properties were also evaluated. The result showed that attributes such as logP and logD were beyond the limits, while other parameters demonstrated favorable results. Within this series, different modifications of the isatin fragment were studied. The most effective modification was benzyl alkylation at the *N*-position. Other attempts involved coupling *N*-position of the isatin with methyl or propyl groups. Among the substituents of the isatin, a fluorine atom was more favorable than other halogens or methyl and nitro groups. However, in *N*-benzylated variant, the most potent compound had an unsubstituted ring. The 4-methoxyphenyl fragment connected to the triazole was crucial for certain non-covalent interactions and was therefore a better choice than the unsubstituted phenyl ring.

**Figure 31:**
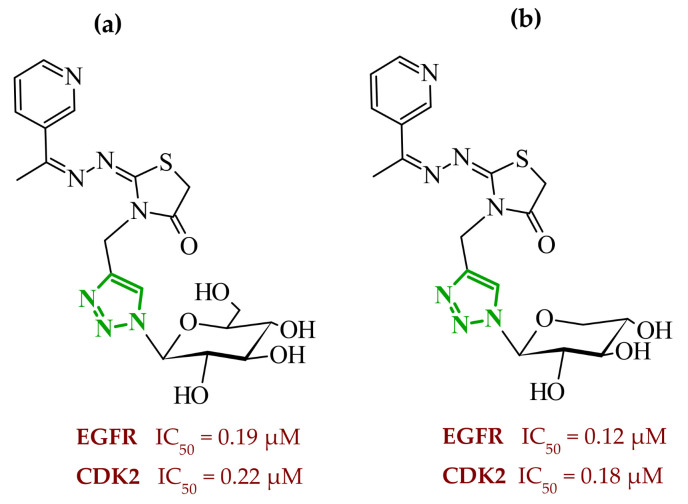
The structures of the most active pyridine-thiazolidinone hybrids bearing sugar moieties: (**a**) **33**; (**b**) **34**-potential EGFR and CDK2 inhibitors [90].

The novel pyridine-thiazolidinone hybrids bearing various sugar moieties or their acyclic analogues were designed by Kassem et al. [90]. The main objective was to incorporate sugar moiety into the primary structure to enhance anticancer activity. Among the novel compounds, glycosides **33** (17) and **34** (18) containing a 1,2,3-triazole motif proved to be potential dual-inhibitors of EGFR and CDK2 (Figure 31). Compounds **33** and **34** exhibited inhibitory activity comparable to that of erlotinib (IC_50_ = 0.15 µM) against EGFR. The lead glycosides additionally displayed better inhibitory activity against CDK2/cyclin A2 kinases than roscovitine (IC_50_ = 0.42 µM). The cytotoxic activities were examined on HepG-2 and MCF-7 cancer cell lines and proved to be excellent with IC_50_ values of 2.43 µM and 0.63 µM for compound **33**, as well as 2.09 µM and 0.51 µM for compound **34** (compared with the IC_50_ values of doxorubicin, 2.85 µM and 1.03 µM, respectively). The most potent compound, glycoside **34**, was found to induce apoptosis and increase the levels of caspase-3 and Bax in MCF-7 cells. Furthermore, all the synthesized hybrids were proven to be safe against the normal WI-38 cell line. The hydroxyl groups from the sugar moieties and sulfur atoms from the thiazolidinone in compounds **33** and **34** participated in hydrogen bonding within the active site of EGFR and CDK2. The hydroxylated glycosides proved to be more efficient than the acetylated ones. Among the tested compounds, structures containing a β-d-galactopyranose fragment were less potent than those containing β-d-glucopyranose or β-d-xylopyranose (the most active). The presence of the 1,2,3-triazole ring was crucial for improved activity and proper binding site orientation in both enzymes.

**Figure 32:**
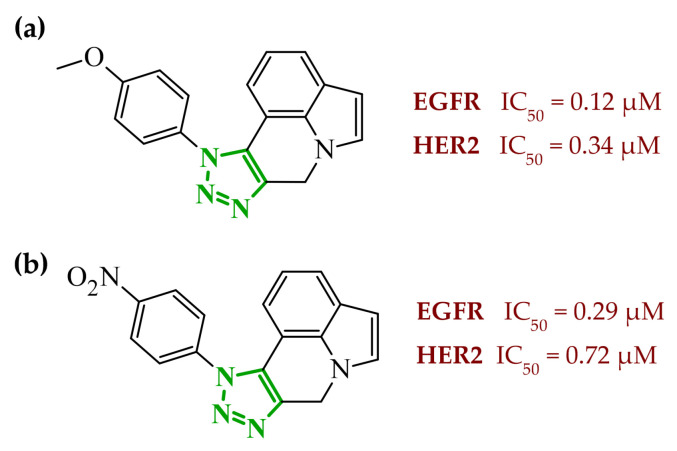
The structures of the most active quinolone-fused 1,2,3-triazole derivatives: (**a**) **35**; (**b**) **36**-potential EGFR and HER2 inhibitors [112].

Lavunuri et al. synthesized quinolone-fused 1,2,3-triazole derivatives as potential anti-breast cancer agents targeting EGFR and HER2 [112]. They used the quinolone moiety and the 1,2,3-triazole ring because these fragments are considered promising in anticancer therapy. Additionally, they introduced an aromatic ring with various substituents to control lipophilicity. Compounds **35** (5a) and **36** (5j) exhibited the best properties against EGFR and HER2 (Figure 32). Both compounds showed better inhibitory activity against EGFR than erlotinib (IC_50_ = 0.42 µM). Furthermore, compound **35** proved to be more effective in inhibiting HER2 than lapatinib (IC_50_ = 0.58 µM). In vitro screening against MCF-7, MDA-MB-468 and MDA-MB-231 cancer cells showed that these two derivatives exhibited higher activity than 5-fluorouracil (**35**: IC_50_ = 2.8 µM, 3.7 µM and 6.4 µM; **36**: 3.5 µM, 4.1 µM and 7.5 µM; 5-FU: 12.5 µM, 7.7 µM and 10.9 µM). Based on molecular docking studies, compound **35** formed hydrogen bonds with residues Lys721 and Met769 of EGFR and with residues Leu785 and Asp863 of HER2, in both cases via the methoxy substituent in the phenyl ring and the triazole scaffold. Compound **36** also formed hydrogen bonds with Lys721 and Met769 of EGFR and, additionally, created a salt bridge with Asp831 residue through the nitro group attached to the phenyl ring. Whereas hydrogen bonding between compound **36** and HER2 involved Leu865 and Asp863 residues. The substituents in the phenyl ring play a crucial role in the activity of the compounds within the series. The weakest anticancer activity was shown by compound with an unsubstituted phenyl group. The electron-donating methoxy group or the electron-withdrawing nitro group at the *para* position yielded the best results, outperforming halogens as well as the methyl or the nitrile group at the *para* or *meta* positions. Compounds bearing 3,5-di-OMe and 3,5-di-NO_2_ substituents did not exhibit enhanced activity. Pharmacokinetic studies showed that compound **35** and **36** met Lipinski’s rule of five, Ghose’s rule, Veber’s rule, Egan’s rule, and Muegge’s rule.

**Figure 33:**
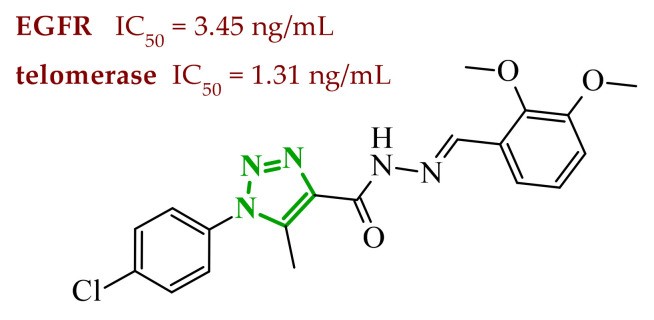
The structure of the most active triazole tethered Schiff base (**37**)-a potential EGFR and telomerase inhibitor [113].

New triazole-tethered Schiff base anticancer agents were developed by Zeidan et al. [113]. The compounds were designed based on pharmacophores of EGFR and telomerase inhibitors with concrete modifications. EGFR and telomerase inhibitors are based on two hydrophobic regions connected by a NH spacer and a flat aromatic ring (which interacts with the adenine region of EGFR) or by a four-atom linker (a telomerase variant). Zeidan’s compounds consisted of various benzylidene motifs and a substituted phenyl-1,2,3-triazole scaffold connected via a methylene hydrazide motif. The most promising regulator was compound **37** (5g) (Figure 33), which showed significant inhibition of EGFR and telomerase. Furthermore, compound **37** exhibited the lowest IC_50_ values in the entire series for HNO97, HCT116, A375, and HEPG2 cancer cell lines (13.31, 13.31, 12.62, and 31.19 μM, compared with values 9.01, 10.46, 14.52, and 10.96 μM, respectively, for doxorubicin). The safety of compound **37** was evaluated based on its low GI% in normal cell lines HSF and OEC. Compound **37** also displayed potent apoptotic activity by upregulating proteins such as caspases-3, -8, -9, and downregulating anti-apoptotic proteins (CDK2/4/6). Moreover, compound **37** induced cell cycle arrest at the G0–G1 phase. Compound **37** complied with Lipinski’s rule of five. Pharmacokinetic studies also indicated favorable oral bioavailability, including good gastrointestinal absorption and no interaction with P-glycoprotein. The molecular docking studies revealed that the triazole moiety in compound **37** formed two π-hydrogen bonds with Cys773 and Leu694 in the EGFR binding pocket. In the case of telomerase, compound **37** exhibited one hydrogen bond and two π-hydrogen bonds with Arg486 through the triazole ring, as well as an additional π-hydrogen bond with Leu554 through the benzylidene motif. According to SAR studies, various halogen or nitro substitutions at position 4 of the phenyl ring attached to the triazole were examined. Different numbers and positions of methoxy groups in the benzylidene fragment were also studied. The lead hit is characterized by a chlorine atom attached to the phenyl ring and 2,3-dimethoxy substituents in the benzylidene fragment. A derivative containing a fluorine atom in the phenyl ring and 3,4-dimethoxy substituents in the benzylidene part also exhibited a high GI% value. Compounds with a nitro group instead of a halogen atom showed weaker activity.

**Figure 34:**
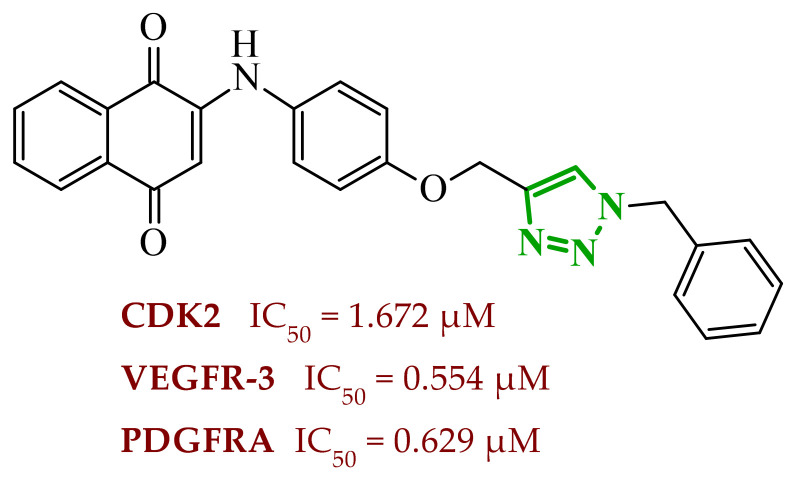
The structure of the most active 1,4-naphthoquinone-1,2,3-triazole hybrid (**38**)-a potential CDK2, VEGFR-3 and PDGFRA inhibitor [114].

There are also 1,2,3-triazole-based potential anticancer agents that act simultaneously on more than two molecular targets and therefore have potential as multi-kinase inhibitors. Mardaneh et al. synthesized 1,4-naphthoquinone-1,2,3-triazole hybrids aimed at CDK2, VEGFR-3 and PDGFRA (platelet-derived growth factor receptor alpha) [114]. The group introduced naphthoquinone moiety instead of a heteroaromatic ring (quite common in receptor tyrosine kinase inhibitors), i.a. to improve the antiproliferative effect. The naphthoquinone fragment was placed on one side of the molecule and the aryl-1,2,3-triazole (linked by a phenoxy group) on the other side. Compound **38** (4a) worked the best as a potential inhibitor of three molecular targets, despite not having the highest in vitro activity against cancer cells among the entire series (Figure 34). Compound **38** exhibited 73% inhibition of CDK2/cyclin A, 87% inhibition of VEGFR-3 and 71% inhibition of PDGFRA at a concentration of 10 μM. Compound **38** showed antiproliferative effects against EBC-1 (IC_50_ = 5.1 µM), AsPC-1 (IC_50_ = 23.5 µM) and HT-29 (IC_50_ = 21.9 µM) cancer cells. The values for doxorubicin were 0.5 µM, 1.73 µM and 0.6 µM, respectively. Furthermore, compound **38** stood out, sparing NIH3T3 non-cancer cell line with the best result (IC_50_ = 85.5 µM), being much better than that of doxorubicin (IC_50_ = 13.0 µM). The derivative also resulted in S phase arrest and caused apoptosis in PDAC cells (fragmentation and nuclear shrinkage). The lead compound complied with Lipinski’s rule of five and exhibited oral bioavailability. According to molecular docking studies, compound **38** formed strong non-covalent interactions with the binding pockets of the selected molecular targets. The carbonyl group of naphthoquinone created hydrogen bonds with amino acids residues within the pockets of these three enzymes. Various π interactions with aromatic rings in compound **38** (phenoxy ring, triazole ring, benzyl ring and/or 1,4-naphthoquinone ring) were observed. Van der Waals interactions also contributed to the binding mode of compound **38** to the receptors. According to SAR analysis, compound with an unsubstituted aromatic ring was found to be the most suitable as potential multikinase inhibitor. Quite good results were also obtained for the compound with a 4-methyl group. At position 4, fluorine, bromine, chlorine atoms, as well as isopropyl and *tert*-butyl groups, were also tested, but they did not exhibit sufficient multi-kinase activity. However, derivatives containing 4-Br, 4-Cl, 3,4-di-Cl, 4-isopropyl substituents yielded better antiproliferative effects in in vitro studies on cancer cells (they simultaneously showed weaker sparing of normal cells). A compound with a more extended heterocycle-indolinone (instead of the benzyl group), was also tested, but it did not demonstrate antiproliferative activity.

**Figure 35:**
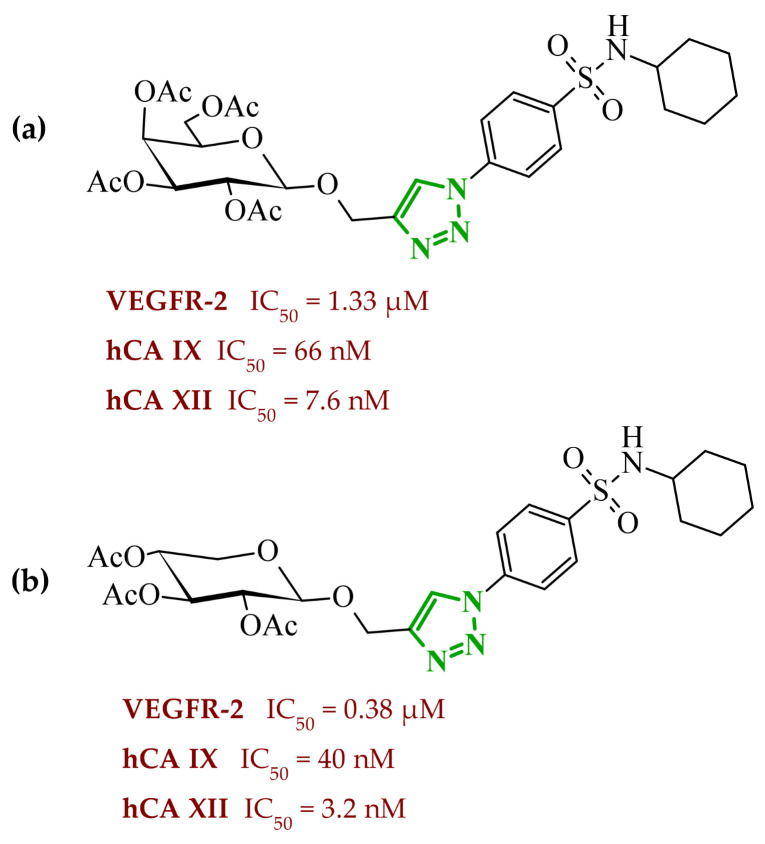
The structures of the most active sulfonamide-based glycosides: (**a**) **39**; (**b**) **40**-potential VEGFR-2 and the carbonic anhydrase isoforms hCA IX and hCA XII inhibitors [115].

Abbas et al. synthesized new sulfonamide-based glycosides, which are composed of three potential anticancer fragments: benzenesulfonamide, 1,2,3-triazole, and a sugar moiety [115]. The series was tested for its inhibitory activity against VEGFR-2 and the human carbonic anhydrase isoforms hCA IX and hCA XII. hCA IX and hCA XII are membrane-bound metalloenzymes catalyzing conversion of carbon dioxide into bicarbonate and proton, which are highly expressed in cancer cells. These isoforms regulate extra- and intracellular pH (hypoxic conditions) and contribute to tumorigenesis and metastasis in solid tumors [116]. Compounds **39** (7) and **40** (9) showed promising results against these three molecular targets when compared to sorafenib and SLC 0111 (IC_50_ = 0.43 μM, 53 nM and 4.8 nM, respectively) (Figure 35). The selected compounds were active against HepG-2 and MCF-7 cancer cells with IC_50_ values of 10.45 μM and 8.39 μM (HepG-2), as also 20.31 μM and 21.15 μM (MCF-7) with doxorubicin serving as a standard drug (IC_50_ = 13.76 μM against HepG-2 and 17.44 μM against MCF-7). Compounds **39** and **40** were also tested against the normal cell line RPE-1 and exhibit low cytotoxicity toward normal cells. Further investigations showed that compound **40** was able to arrest MCF-7 cells in the G2/M phase of the cell cycle. Moreover, compound **40** increased Bax levels and downregulated Bcl-2 protein, as well as increased p53 protein levels in MCF-7 cells (compared to control cells). In silico studies revealed that the lack of substituents at position 6 in the glycoside part of compound **40** improved its orientation and increased interactions within the binding sites of the chosen targets. In both cases, the sulfonamide oxygen formed hydrogen bonds with residue Cys919 of VEGFR. The acetyl oxygen of the glycoside in compound **39** created a hydrogen bond within the binding site of VEGFR (with residue Leu840), while compound **40** adopted a different position, allowing a nitrogen atom from the triazole moiety to form a hydrogen bond with Asn923. The oxygen atoms in the sulfonamide part also proved crucial for interacting with isoforms hCA IX and hCA XII. Compounds **39** and **40** formed ionic bonds with a zinc ion inside the active site of these two isoforms and additionally generated hydrogen bonds (mainly with Thr200). The proper orientation of compound **40** also enabled interactions between the acetyl oxygens in the sugar part and Gln67 of hCA IX. The residue Lys67 of hCA XII interacted, depending on the orientation, with the triazole moiety of compound **39** or the acetyl oxygens of compound **40**. SAR studies revealed that peracetylated xylopyranose performed better than peracetylated galactopyranose. Additionally, compounds with free hydroxyl groups in the sugar moieties showed weaker anticancer activity. Another modification within the series involved changing the cyclohexyl group attached to the benzenesulfonamide part to a butyl substituent, which did not result in promising outcomes.

**Figure 36:**
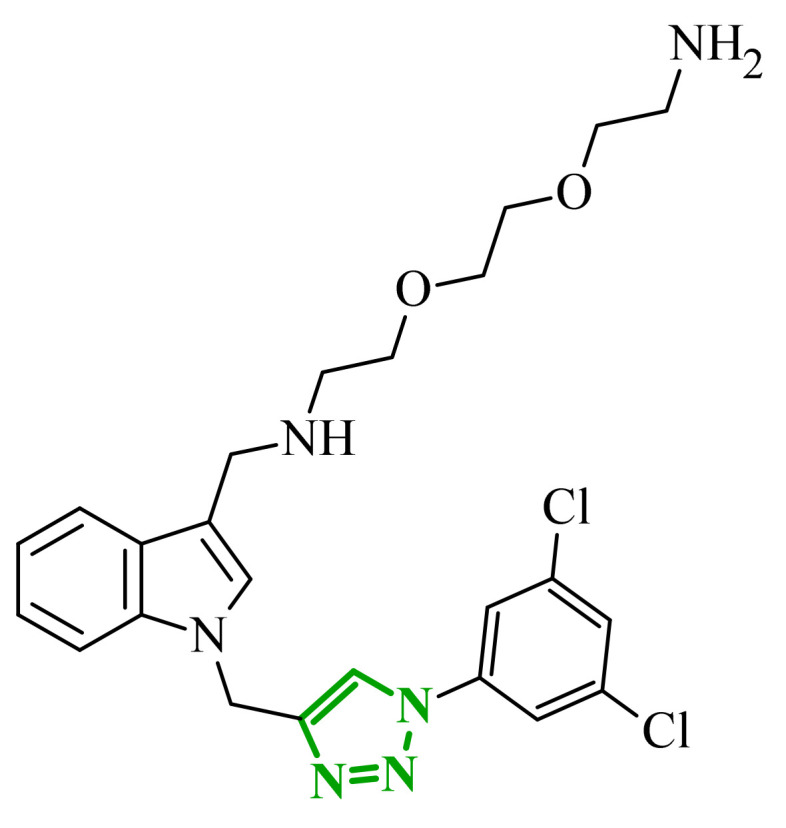
The structures of the most active triazolyl–indole derivative (**41**)-potential *c-KIT* and *KRAS* genes inhibitor [117].

A different approach was presented by Bhattacharyya et al., who described the design and synthesis of a series of triazolyl–indole derivatives developed as ligands capable of interacting with G-quadruplex (G4) structures located in the promoter regions of oncogenes associated with the development of leukemia [117]. G-quadruplexes are non-canonical DNA structures formed in guanine-rich sequences that occur, among others, in telomeres and in the promoter regions of numerous genes regulating cellular proliferation. Stabilization of these structures by small molecules can lead to inhibition of oncogene transcription; therefore, G4 structures represent a promising therapeutic target in anticancer therapy. In the discussed study, the authors focused on G-quadruplexes present in the promoter regions of the *c-KIT* and *KRAS* genes, which play an important role in the pathogenesis of leukemia. The *c-KIT* gene encodes a receptor tyrosine kinase responsible for regulating cell proliferation and survival, whereas *KRAS* is one of the key regulators of signaling pathways controlling cellular growth. Overexpression or mutations of these genes lead to uncontrolled proliferation of cancer cells; therefore, their simultaneous modulation constitutes an attractive therapeutic strategy. Analysis of the results obtained from the FRET-melting assay revealed clear structure–activity relationships within the studied series of compounds. Among all synthesized derivatives, compound **41** (TI12) (demonstrated in Figure 36) displayed particularly high activity, whereas most of the remaining analogues exerted only a minor influence on the stability of the analyzed structures. The lead structure exhibited pronounced selectivity toward the G-quadruplexes located in the promoter regions of *c-KIT1* and *KRAS*, indicating preferential interaction with these DNA regions. The obtained results suggest that the presence of appropriately positioned aromatic and hydrophobic groups promotes π-π interactions between the ligand and the guanine tetrad planes forming the G-quadruplex. In the case of compound **41**, the combination of an indole ring with a suitably substituted triazole moiety enables favorable fitting of the molecule to the G-quadruplex surface and facilitates stabilizing hydrophobic and hydrogen-bond interactions. In contrast, compounds containing shorter or more polar side chains showed a marked decrease in stabilizing activity, indicating the important role of hydrophobic properties in the recognition of DNA structures. Additionally, it was observed that increasing the steric bulk of substituents in the triazole moiety favored selective interactions with G-quadruplexes in the promoter regions of the *c-KIT* and *KRAS* genes, whereas smaller and more polar substituents resulted in reduced affinity for G4 structures. The interaction of the ligands with DNA was further confirmed by fluorescence intercalator displacement (FID) experiments. In these assays, compound **41** demonstrated a significant ability to interact with G-quadruplexes, showing particularly high affinity for G4 structures associated with the *c-KIT* and *KRAS* genes while displaying no substantial interaction with canonical double-stranded DNA. These findings indicate the selectivity of triazolyl–indole derivatives toward non-canonical DNA structures. Molecular docking simulations revealed that compound **41** interacts with guanine bases forming the G-quadruplex structure in the promoter regions of *KRAS* and *c-KIT1*, establishing numerous hydrogen bonds and hydrophobic interactions that stabilize the ligand–DNA complex. In the *KRAS* structure, the ligand interacted with bases DG3, DG4, DG9, DG13, and DA22, whereas in the *c-KIT1* structure interactions were observed with DG2, DG6, DG10, DG13, and DA1, forming several stabilizing hydrogen bonds and π-π interactions. The calculated binding energy values indicated favorable accommodation of the ligand on the G-quadruplex surface and stability of the resulting complex.

The biological activity of the investigated compounds was subsequently evaluated in a panel of human cancer cell lines. Among all analyzed derivatives, **41** exhibited selective cytotoxicity toward leukemia K562 cells, reaching an IC_50_ = 6.79 μM, whereas its activity toward other cancer cell lines was considerably weaker. In contrast, *N*,*N*-dimethylpropane-1,3-diamine derivative showed less selective activity and displayed cytotoxic effects also against other tested cell lines. These results suggest that the structural modifications introduced in molecule **41** promote both biological selectivity and preferential interaction with selected G-quadruplex structures. Importantly, the authors also demonstrated that simultaneous inhibition of both oncogenes leads to a synthetic lethality effect in leukemic cells. Gene-silencing experiments revealed that inhibition of only one of the genes did not result in a significant decrease in cell viability, whereas concurrent inhibition of both *c-KIT* and *KRAS* pathways produced a markedly stronger cytotoxic effect. These findings indicate that ligands stabilizing G-quadruplexes may modulate several key oncogenic pathways simultaneously, thereby increasing their therapeutic potential in the treatment of leukemia.

#### 2.3.2. 1,2,4-Triazole Derivatives

**Figure 37:**
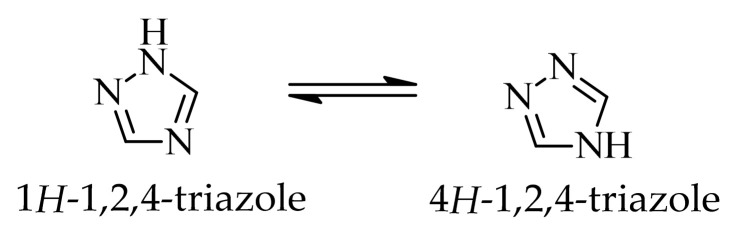
Two isomeric forms of aromatic 1,2,4-triazoles [118].

**Figure 38:**
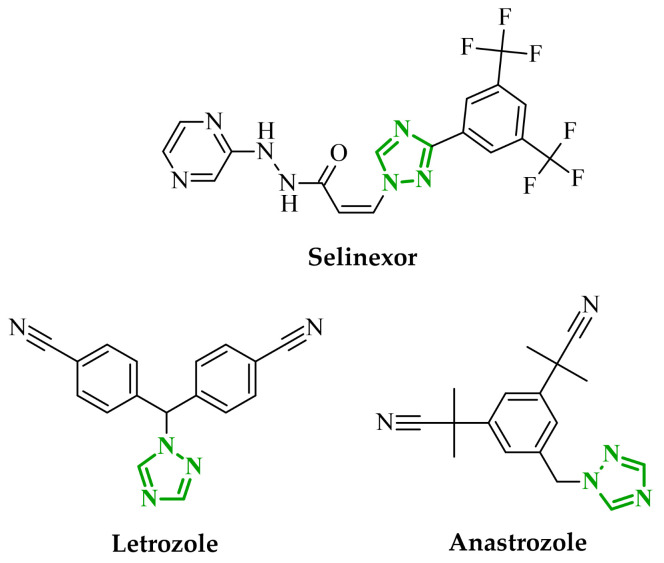
Selected examples of drugs incorporating the 1,2,4-triazole fragment (two isomeric forms included) [128,129].

1,2,4-Triazoles, similar to 1,2,3-triazoles, can be divided into two isomeric aromatic forms, 1*H*-1,2,4-triazoles and 4*H*-1,2,4-triazoles, with the 1*H*-1,2,4-triazole scaffold being the more stable form (Figure 37) [118]. The 1,2,4-triazole motif is often used in medicinal chemistry due to its variety of beneficial properties, e.g., anti-inflammatory [119], anticancer [27,120,121], antibacterial [122], antiviral (fostemsavir and ribavirin are commercially available) [123,124], antifungal (itraconazole, fluconazole, voriconazole are commercially available) [125], and anticonvulsant [126] activities. In particular, 1*H*-1,2,4-triazoles are an outstanding scaffold in the search for novel anticancer drugs targeting the ATP-binding site of proteins. Owing to the 1,2-di-nitrogen substitution pattern, which is often assisted by additional heteroatoms at positions 3 and/or 5, the molecule can bind to the kinase hinge region through H-bond interactions [127]. In addition to kinase inhibitors, there are a few other approved anticancer drugs based on the 1,2,4-triazole moiety. Selinexor is XPO1 (exportin-1) inhibitor that in combination with dexamethasone is used in adult patients with relapsed or refractory multiple myeloma (RRMM) (Figure 38). It is also being investigated in other clinical trials for various types of cancer [128]. Other examples include letrozole and anastrozole, which are aromatase inhibitors used as adjuvant endocrine therapy in the treatment of postmenopausal women with hormone-sensitive breast cancer (Figure 38) [129]. It has been demonstrated that 1,2,4-triazole derivatives exhibit favorable pharmacokinetic properties. For instance, selinexor is absorbed relatively rapidly following oral administration, reaching peak plasma concentrations within approximately 2–4 h. A further characteristic that merits attention is the substantial volume of distribution, indicative of its capacity to permeate numerous bodily tissues. The metabolism of selinexor occurs primarily via the CYP3A4 enzyme, and elimination takes place mainly via the hepatobiliary route with excretion of metabolites in the feces; the plasma half-life is approximately 6–8 h [130]. Anastrozole also exhibits good bioavailability following oral administration and moderate lipophilicity. It has been hypothesized that while it does not act as a substrate for P-glycoprotein, it may be metabolized by cytochrome P450 enzymes [131].

**Figure 39:**
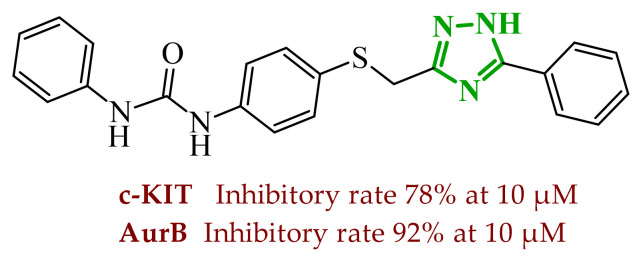
The structure of the most active 1,2,4-triazole derivative (**42**)-a potential c-KIT and AurB inhibitor [127].

Quattrini et al. sought dual c-KIT and the Aurora family (Aur, also ARK) inhibitors for the treatment of melanoma [127]. The tyrosine kinase receptor c-KIT is an important factor in initiation and progression of melanoma, while the serine/threonine kinase family Aur plays a significant role in cell division. A receptor-based virtual screening campaign led to the discovery of a 1,2,4-triazole derivative bearing a 4-chlorophenyl substituent on one side and a (4-chlorophenylthio)methyl substituent on the other. The structure was optimized by replacing the chlorine atom, bound to the (phenylthiol)methyl moiety with variously substituted phenyl-urea residues and by introducing modifications to the ring at the opposite end of the molecule. Compound **42** (6a) exhibited the best anticancer activity (Figure 39). It inhibited c-KIT by approximately 78% and AurB by approximately 92% (AurA by about 41%) at a concentration of 10 μM. Antiproliferative efficacy was evaluated against A2058 and WM266-4 cell lines, yielding IC_50_ values of 9.65 μM and 11.41 μM, respectively. Compound **42** bound to the active side of c-KIT through the nitrogen atoms of the triazole ring and hydrogen atoms from the extended urea group, both of which participated in hydrogen bonding. Compounds from the series caused steric clashes with the AurA binding pocket, resulting in lower inhibition efficacy. In contrast, compound **42** fit well into the AurB binding pocket, forming hydrogen bonds via the urea fragment and hydrophobic interactions mediated by the aromatic rings on both sides of the urea moiety. SAR analysis indicated that, for dual inhibition, compounds with an unsubstituted aromatic ring adjacent to the urea fragment showed significantly higher efficacy than their substituted counterparts. Among these compounds, the absence of a substituent near the triazole scaffold was the most favorable, followed by the presence of a chlorine atom, and finally a bromine atom. Compound **42** also exhibited a strong synergistic effect when administered in combination with vemurafenib.

**Figure 40:**
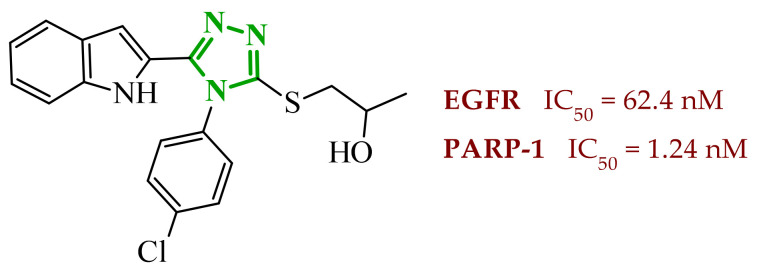
The structure of the most active indolyl-1,2,4-triazole hybrid (**43**)-a potential EGFR and PARP-1 inhibitor [132].

New bioactive indolyl-1,2,4-triazole hybrids acting as EGFR inhibitors were synthesized by Youssef et al. [132]. Additionally to EGFR inhibition, they also aimed at poly ADP-ribose polymerase (PARP) inhibition. The protein is responsible for repairing DNA damage, therefore, reducing its activity may be effective in cancer treatment [132,133]. The series was based on *S*- or *N*-alkylation of indolyl-triazolethiones with acetyl-protected α-halosugars or halogenated alcohols. The most effective derivative was compound **43** (13b) (Figure 40). Compound **43** exhibited EGFR and PARP-1 inhibition. Cytotoxic activity was evaluated against MCF-7 and HepG2 cell lines (IC_50_ = 1.07 μM and 0.32 μM), with erlotinib used as the standard drug (IC_50_ = 2.51 μM and 2.91 μM). Compound **43** was non-toxic to healthy cells. Moreover, compound **43** was shown to induce apoptosis in MCF-7 and HepG2 cells and to cause cell cycle arrest at the G2/M and S phases. Finally, anticancer activity was confirmed in an in vivo SEC-cancer model, where compound **43** improved hematological and biochemical parameters and inhibited tumor proliferation by 66.7% (comparable to erlotinib). Compound **43** showed no deviations from Lipinski’s rule of five and was found to be appropriate as oral drug candidate. Molecular docking approach revealed that key interactions included hydrogen bond formation (with Met793 of EGFR and Gly863 of PARP-1), as well as arene-cation interactions (with Lys745 of EGFR and His862 of PARP-1). The binding energies were −24.97 kcal/mol and −24.32 kcal/mol for docking into PARP-1 and EGFR, respectively. The introduction of glycoside moiety did not increase cytotoxicity. Compounds bearing 2-hydroxyethyl or 2-hydroxypropyl substituents showed improved activity, with the 2-hydroxypropyl group being particularly favorable. Additionally, the presence of a chlorine atom at the position 4 of the phenyl ring attached to the triazole moiety was essential.

**Figure 41:**
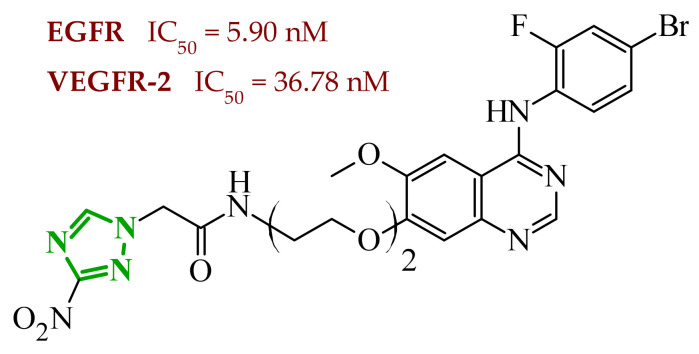
The structure of the most active 4-anilinoquinazoline derivative (**44**)-a potential hypoxia-selective EGFR and VEGFR-2 inhibitor [134].

Many tyrosine kinase inhibitors exhibit side effects because they target molecules that are present in normal healthy cells. One of the solutions is to modify molecules to increase their specificity toward cancer cells by selectively targeting hypoxic regions of tumors. This concept was applied by Wei et al. The team synthesized new 4-anilinoquinazoline derivatives whose main goal was to act as hypoxia-selective EGFR and VEGFR-2 inhibitors [134]. They modified their previous series of nitroimidazole-substituted 4-anilinoquinazoline derivatives (based on vandetanib molecule) by replacing the nitroimidazole moiety (a hypoxia-targeting group but toxic) with a 3-nitro-1,2,4-triazole fragment, which served as a safer and effective hypoxia-targeting group. In addition, various aniline substituents and length of the linker were tested. Among the series, compound **44** (10a) proved to be the most effective dual inhibitor (Figure 41). The selected compound exhibited stronger inhibitory activity toward EGFR than vandetanib (IC_50_ = 19.76 nM). The VEGFR-2 inhibitory activity was comparable to that of vandetanib. Compound **44** also showed better antiproliferative activity than vandetanib in A549 and H446 cells under hypoxic conditions and significantly downregulated *VEGF* gene expression. In vivo experiments demonstrated that compound **44** significantly inhibited tumor growth in A549 xenografts and reduced vandetanib-associated toxicity (weight loss). Molecular docking studies with EGFR revealed the presence of a characteristic interaction with Met793, created by the nitrogen atom of the quinazoline moiety. The aniline moiety of compound **44** was inserted coplanarly into a hydrophobic pocket, while the triazole scaffold was positioned outside the protein. In the VEGFR-2 inhibition, the aniline moiety of compound **44** was also located within a hydrophobic pocket. The position was stabilized by hydrogen bonds involving nitrogen atoms from the quinazoline part. Additionally, two hydrogen bonds were formed at the gate of the hydrophobic pocket via the carbonyl oxygen. The triazole scaffold extended further into the solvent region, adopting a U-shape conformation and created π-π interactions. SAR studies indicated that compounds with 4-bromo-2-fluoro- and 3,4-dichloro-2-fluoro-substituted aniline exhibited the strongest VEGFR-2 inhibitory activity (bulky substituents stabilized the complex due to optimal hydrophobicity and electronegativity). In contrast, EGFR inhibitory activity was influenced by the length of the linker between the triazole and quinazoline rings. The optimal linker length was n = 2 (shortening the linker to n = 1 reduced EGFR inhibitory activity). Shorter (n = 1) and longer (n = 3) linkers also could destabilize the compound **44**-VEGFR-2 complex.

**Figure 42:**
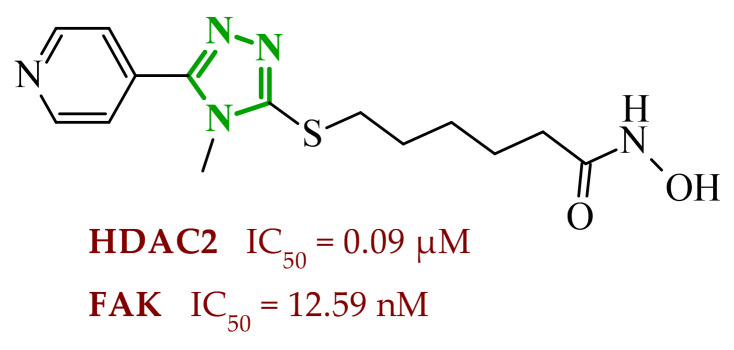
The structure of the most active 5-pyridinyl-1,2,4-triazole derivative (**45**)-a potential HDAC2 and FAK inhibitor [135].

Mustafa et al. investigated molecules that could serve as HDAC and also FAK (focal adhesion kinase) inhibitors, as FAK regulation may disrupt tumor growth and metastasis [135,136]. Analysis of the pharmacophores of these proteins led to the design of 5-pyridinyl-1,2,4-triazole derivatives that target the ATP-binding site of FAK. These derivatives are connected via a specific linker to carboxylic acid, hydroxamic acid, or 2-aminobenzamide moiety, which target the zinc binding group (ZBG) in HDAC. The most promising compound was **45** (6a), which contained a five-atoms aliphatic linker and a methyl substituted triazole moiety (Figure 42). Compound **45** inhibited HDAC2 with an IC_50_ comparable to vorinostat and FAK with an IC_50_ approximately two-fold higher compared to TAE226. Moreover, compound **45** did not enhance Akt activity, a known side effect of some HDAC inhibitors (e.g., valproic acid) [135]. The ability of compound **45** to inhibit renal cancer growth (A-498 and Caki-1 cells with IC_50_ values of 0.95 μM and 1.23 μM) was comparable to that of TAE226 (0.81 μM and 1.05 μM) and superior to vorinostat (25.08 μM and 32.46 μM). Compound **45** demonstrated the potential of arresting the cell cycle at the G2/M phase and inducing apoptosis in A-498 and Caki-1 cells. Further investigations showed that compound **45** inhibited STAT3 phosphorylation and activated caspase-3, -8 and -9. According to in silico studies, the hydroxamic acid part of compound **45** formed a coordinate bond with a zinc atom in the active site of HDAC2 and additionally created three hydrogen bonds. Hydrogen bonds were also formed by the triazole ring. Other interactions included π-H forces involving the triazole and the pyridine rings, as well as the aliphatic linker. In binding to FAK, the pyridinyl fragment was crucial-its nitrogen atom created a hydrogen bond at the kinase hinge region. Moreover, the pyridine ring participated in four π-H interactions. In addition, the sulfur atom and the carbonyl oxygen also formed some hydrogen bonds. Analysis of the compounds’ structures revealed that methyl substituted triazole was preferable to phenyl, allyl, or ethyl substitutions. At the opposite end of the molecule, the hydroxamic acid was superior to the 2-aminobenzamide or the carboxylic acid parts. In this configuration, an aliphatic linker was more effective than an aromatic one.

**Figure 43:**
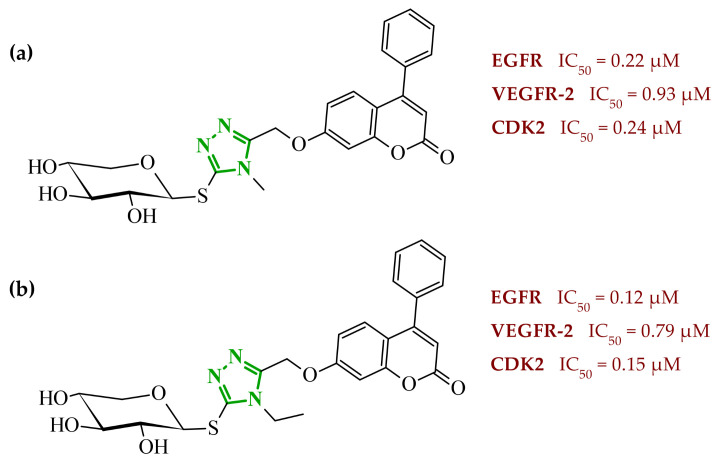
The structure of the most active 1,2,4-triazole-coumarin-thioglycosides: (**a**) **46**; (**b**) **47**–a potential EGFR, VEGFR-2 and CDK2 kinase inhibitors [137].

El-Sayed et al. designed a novel series of anticancer compounds based on 1,2,4-triazole-coumarin-thioglycoside and 1,2,3-triazole-coumarin-glycoside hybrids, as well as their acyclic analogues [137]. The safety profile of the series was evaluated using normal cell lines, revealing that hybrids containing a 1,2,4-triazole moiety and a thioglycoside fragment exhibited low cytotoxicity. Among them, compounds **46** (8) and **47** (10) were the most prominent multi-kinase inhibitors (Figure 43). The selected compounds showed promising inhibitory activity against EGFR, VEGFR-2 and CDK2/cyclin A2 kinases. The standard drugs (erlotinib, sorafenib, and roscovitine) exhibited IC_50_ values of 0.18 μM, 1.58 μM and 0.46 μM. Cytotoxic activity was evaluated against MCF-7 cells. Compounds **46** and **47** showed IC_50_ values of 52.2 μM, and 19.6 μM. Moreover, compound **47** upregulated the pro-apoptotic Bax protein and downregulated the anti-apoptotic Bcl-2 protein. Compound **47** induced cell cycle arrest at the S phase. Molecular docking studies with EGFR revealed that compounds **46** and **47** fit well into the binding site through arene-cation interactions, involving the coumarin moiety, as well as hydrogen bonding interactions, involving the glycoside fragment. Compound **47** additionally exhibited interactions near the triazole scaffold (through the ethyl group and the methoxy linker). For VEGFR-2, arene-cation interactions (the coumarin scaffold and the triazole moiety) and hydrogen bonds (the glycoside moiety) were also important for anchoring the ligand within the binding pocket. Inhibition of CDK2/cyclin A2 by compounds **46** and **47** was associated with hydrogen bonds formed by the carbonyl group from the coumarin part and also the glycoside fragment. Substituents on the triazole scaffold influenced the orientation of the glycoside moiety, enabling the sulfur atom of compound **46** to participate in hydrogen bonding. In contrast, compound **47** showed more interactions within the glycoside region. SAR studies indicated that the presence of a 1,2,3-triazole ring or a glycoside part improved anticancer activity but was also associated with higher toxicity toward normal cells. The optimal hits were compounds containing a 1,2,4-triazole. Among the thioglycosides, the xylopyranosyl moiety was more favorable than the galactopyranosyl one, and thioglycosides with free hydroxyl groups were more potent than acetylated derivatives. The ethyl group was a better substituent to the triazole scaffold than the methyl group, which explains why compound **47** outperformed compound **46**.

**Figure 44:**
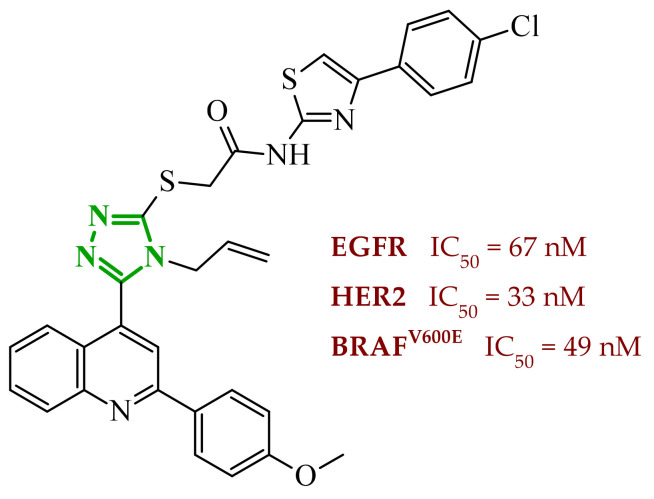
The structure of the most active thiazole-1,2,4-triazole-quinoline hybrid (**48**)-a potential EGFR, HER2 and BRAF inhibitor [140].

Mohassab et al. sought to improve their previous series and synthesized novel thiazole-1,2,4-triazole-quinoline hybrids targeting EGFR, HER2 and BRAF^V600E^ [138,139,140]. The most active compound was **48** (8f), which outperformed erlotinib in inhibiting the EGFR receptor (Figure 44). Although **48** did not achieve the same efficacy as lapatinib for HER2 or vemurafenib for BRAF^V600E^, it still showed promise against these targets. The antiproliferative activity of compound **48** was evaluated against various cancer cell lines—HT-29, Panc-1, A-549 and MCF-7. In all cases, compound **48** (average GI_50_ = 25 nM) surpassed the reference drug erlotinib (average GI_50_ = 33 nM), while remaining safe toward normal cells. Moreover, compound **48** was found to induce apoptosis through activation of caspases-3, -8, -9, p53 and Bax as well as by Bcl-2 downregulation. According to in silico pharmacokinetic analysis, the lead structure was predicted not to be a P-glycoprotein substrate and did not permeate the blood-brain barrier, while also having the potential to inhibit CYP2C9 and CYP3A4. Violations of Lipinski’s rule of five involved molecular size and lipophilicity. In EGFR binding, a crucial hydrogen bond was formed between the amide linker and the residue Met769. Compound **48** was further strongly stabilized within the binding site by the quinoline moiety and its methoxyphenyl substituent, the triazole and the thiazole scaffold and its chlorophenyl group. In case of BRAF^V600E^ binding, strong interactions were formed by the thiazole ring, such as hydrogen bonding, π-π-sulfur and π-π-alkyl interactions. Additional π-π-alkyl interactions involved the triazole ring and were observed near the hinge region. The chlorophenyl group was positioned within a hydrophobic subpocket via a π-π-alkyl interaction. Characteristic interactions were also observed for HER2 inhibition. The thiazole ring anchored the molecule in a polar-hydrophobic interface through hydrogen bonds and hydrophobic contacts. Other interactions were mediated by the triazole ring in hinge-proximal region, the quinoline fragment in the adenine-binding part, the chlorophenyl substituent in a lipophilic subpocket and the methoxyphenyl group, which supported proper solvent-channel orientation. Within the series, various substituents at position 4 of the phenylquinoline group and of the phenylthiazole group were evaluated simultaneously. The most favorable results were obtained for compounds with a combination of the methoxyphenyl group (R) attached to the quinoline moiety and the chlorophenyl group (R^1^) attached to the thiazole ring. Comparatively good results were also observed for the derivatives with chlorophenyl group (R) combined with the nitrophenyl group (R^1^) as well as with the methylsulfonyl substituent (R) paired with the chloro substituent (R^1^). The least favorable results were obtained when methoxy (R) and nitro (R^1^) groups were present.

**Figure 45:**
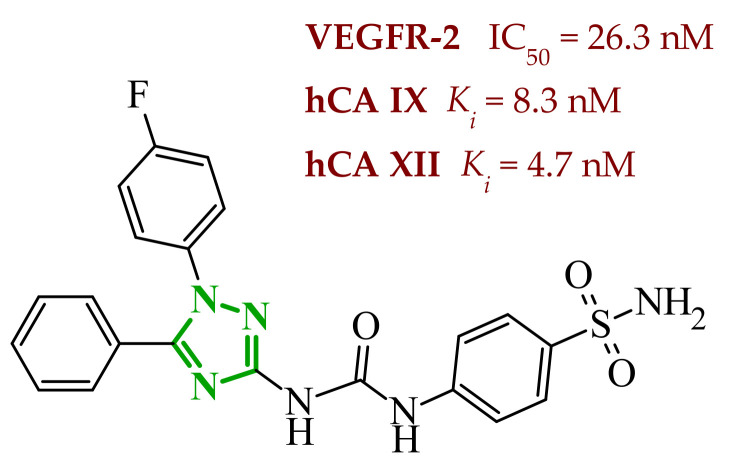
The structure of the most active 1,5-diaryl-1,2,4-triazole-tethered sulfonamide (**49**)-a potential hCA IX, hCA XII and VEGFR-2 inhibitor [141].

Elsawi et al. aimed to design hCA IX, hCA XII and VEGFR-2 inhibitors, as these molecular targets are highly expressed under hypoxic conditions and play a vital role in cancer cells survival [141]. The team focused on 1,5-diaryl-1,2,4-triazole-tethered sulfonamides as SLC-0111 analogues. Specifically, they replaced the 4-fluorophenyl, introducing a 1,5-diaryl-1,2,4-triazole moiety, where one phenyl ring bore various substituents. They also investigated how linker elongation and changes in the position of the sulfamoyl moiety influenced the biological activity. The lead derivative was compound **49** (13a), which differed from SLC-0111 only by the insertion of a phenyl substituted triazole scaffold (Figure 45). The inhibitory potential against hCA isoforms was tested using the stopped-flow CO_2_ hydrase assay. Compound **49** inhibited hCA IX and hCA XII with *K_i_* values lower than acetazolamide. Moreover, compound **49** showed good selectivity toward hCA IX and XII isoforms over the ubiquitous off-target hCA I isoform. Inhibition of VEGFR-2 was also high (IC_50_ for sunitinib = 39.7 nM). Additionally, the inhibitory activity of compound **49** was evaluated against 50 key kinases in the human kinome, and the molecule demonstrated good selectivity toward VEGFR-2. The antiproliferative activity of compound **49** was assessed under hypoxic conditions against MCF-7 and T47D cell lines, yielding IC_50_ values of 0.66 nM and 4.51 nM, which were significantly better than those of staurosporine (3.18 nM and 7.12 nM, respectively). Compound **49** was found to be safe for normal breast cell line MCF-10A. Molecular docking studies with hCA isoforms revealed that, in both cases, the benzenesulfonamide residue of compound **49** occupied the active site of the enzymes and formed a characteristic coordination bond between a zinc atom and the sulfonamide group. Another similarity involved the benzenesulfonamide moiety forming hydrogen bonds via an oxygen atom and π-π interactions through the aromatic ring. Compound **49** additionally created a hydrogen bond via the carbonyl group of the linker within the hCA IX active site and π-cation interactions involving the 4-fluorophenyl moiety within the hCA XII active site. Interactions with VEGFR-2 were mediated by two hydrogen bonds formed by the oxygen atom of the sulfonamide part and the nitrogen atom of the ureido linker. π-π stacking involving the benzenesulfonamide residue was also important. SAR analysis indicated that compounds containing an ethylureido linker did not exhibit sufficient cytotoxic activity. Among the remaining compounds, those bearing the sulfamoyl group at position 4 were the most promising. For these molecules, a 4-fluorophenyl substituent in the triazole ring was preferred over a chlorine atom or a methoxy group at position 3 or/and 4.

### 2.4. Summary and Cross-Scaffold Comparison

Heterocyclic scaffolds used in multi-target anticancer ligand design are not interchangeable but fulfill distinct and complementary functional roles within molecular architectures. Imidazole, pyrazole, and triazole systems differ not only in their preferred biological targets but also in the way they contribute to multitarget activity, including direct target engagement, dual pharmacological modulation, and structural integration of pharmacophoric units.

**Figure 46:**
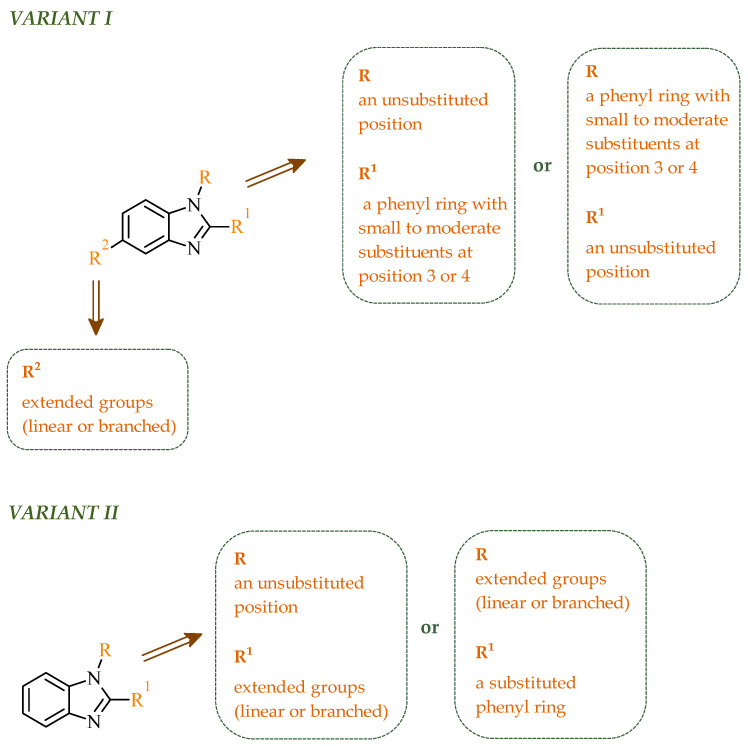
Common features of benzimidazole-based potential multi-target directed ligands.

Imidazole-based systems represent highly versatile scaffolds in multi-target anticancer drug design, with a clear preference toward kinase inhibition, including VEGFR, EGFR, PI3K/mTOR, CDK2, and RAF-related targets. A key feature of imidazole derivatives is their frequent incorporation into hybrid systems, enabling the integration of pharmacophores targeting diverse proteins. This multitarget activity is typically achieved through pharmacophore hybridization or linking strategies in which the imidazole core often acts as a central interaction motif within the molecular architecture. Among the collected structures, imidazole was present either as a single ring or in a fused benzimidazole form, where the bulkier one, benzimidazole, was more prevalent. The imidazole ring, when present alone, was typically placed in the center of molecules, usually with 2–3 bulky aromatic substituents, without a comparable characteristic pattern of substitution among potential multitarget lead structures. Aromatic substituents promote favorable hydrophobic interactions with residues forming the kinase binding cavity, while the imidazole core itself enables key interactions with hinge region residues, stabilizing the ligand-enzyme complex. On the other hand, benzimidazole was found to play two roles as a pharmacophore in the collected examples—it served as a central part of linear molecules with bulky substituents on both sides (variant I) or the unsubstituted benzimidazole (at the phenyl positions) was placed at one end of the molecule (variant II). Analysis of the representative molecules led to the identification of characteristic pharmacophoric features, which may contribute to the design of universal imidazole-based structures acting as multitarget agents. The structural properties that may enhance activity are shown in Figure 46. In variant I, the *C*-6 position of benzimidazole was unsubstituted, while linkers bearing heterocyclic or aromatic systems were attached to the *C*-5 position. On the other side of the molecule, a substituted phenyl moiety was connected to the atom *C*-2 (less frequently to *N*-1). The common substituents on the phenyl ring were a metoxy group or a chlorine atom. Structure-activity relationship analysis indicated that biological activity was strongly governed by substitution patterns within the benzimidazole core. In variant II, benzimidazole played the role of the substituent, mostly without additional groups or atoms attached to the aryl fragment. In this case, benzimidazole was attached to the main core by the atom *C*-2 or *N*-1. The remaining parts of the molecules included linkers and additional aromatic rings. In both cases, linker composition and flexibility significantly influenced multitarget activity, as they determine the spatial arrangement of pharmacophoric fragments and enable simultaneous accommodation within distinct binding sites. In particular, optimized linker length and configurational adaptability are critical for achieving balanced activity across multiple targets. The described imidazole-based molecules are collected in Table 1, together with a comparison of key SAR features within each series.

**Figure 47:**
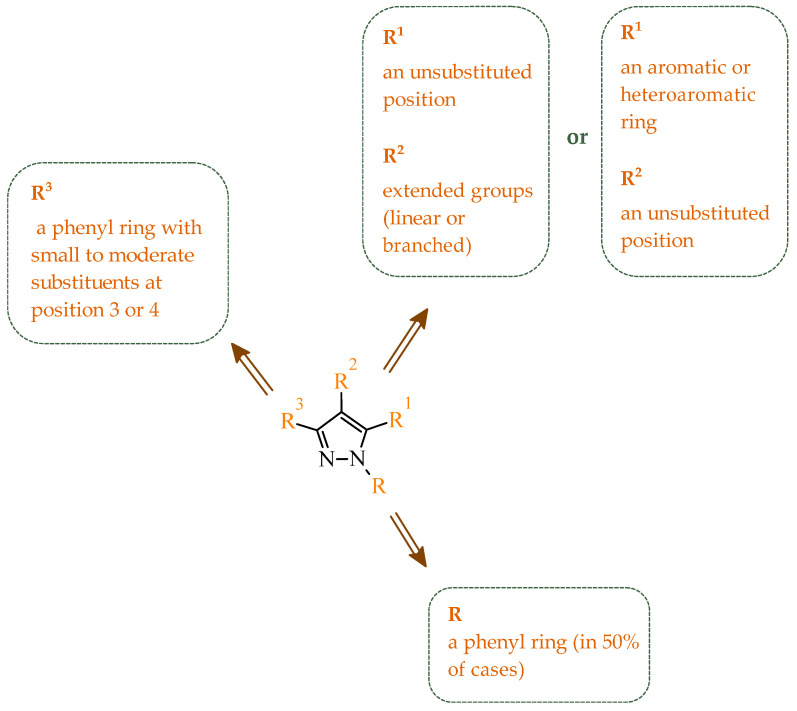
Common features of pyrazole-based potential multi-target directed ligands.

Pyrazole-based derivatives exhibit a distinct dual-activity profile that integrates modulation of proliferative signaling pathways and inflammation-related targets. The appropriate functionalization of the pyrazole core enables efficient targeting a variety of molecular targets. Beyond common EGFR/COX-2 dual targeting, pyrazole-based systems have been successfully extended toward additional kinase targets, including VEGFR, CDK2, ERK, JNK, and BRAF, confirming their adaptivity as a scaffold for multitarget design. This versatility also arises from the ability of pyrazole scaffolds to accommodate diverse substitution patterns, enabling both hydrophobic interactions within the kinase binding pockets and stabilizing hydrogen bonds with hinge region residues. Among the collected molecules, the pyrazole ring was placed in the center, forming branched molecules with a high degree of freedom in spatial arrangement (which is also related to the variety of synthetic approaches used for pyrazole derivatives). Additionally, in few cases, 4,5-dihydro-1*H*-pyrazoles or pyrazolones were present instead of the standard pyrazole ring. Based on selected examples of pyrazole-based compounds, several common features can be identified (Figure 47). The *N*-1 position of the pyrazole ring was in approximately half of the cases attached to a phenyl group (unsubstituted or substituted at the *para* position), although it was not a general rule. Both larger extended fragments and, conversely, short aliphatic substituents were also observed, and it was strictly dependent on the overall molecular architecture. When the *C*-2 position was unsubstituted, the *C*-3 position was typically attached to larger extended fragments. However, the *C*-2 position was also frequently connected to heteroaromatic (e.g., thiophene or imidazole moieties) or aromatic rings, which resulted in the *C*-3 position remaining unsubstituted. The incorporation of heteroatom-containing substituents and aromatic moieties facilitated effective binding across structurally diverse kinase active sites. The *C*-4 position was generally occupied by a phenyl ring with substitution at the *meta* or/and *para* positions. In many cases, the diarylpyrazole framework (connected via *N*-1 and *C*-4) was present and of high importance. For example, it was strongly associated with COX-2 selectivity. Furthermore, structure-activity relationship analysis demonstrated that biological activity was strongly governed by substitution patterns on both the pyrazole ring and its aryl substituents. The variety of pyrazole-based systems are collected in Table 2, together with a comparison of key SAR features within each series.

**Figure 48:**
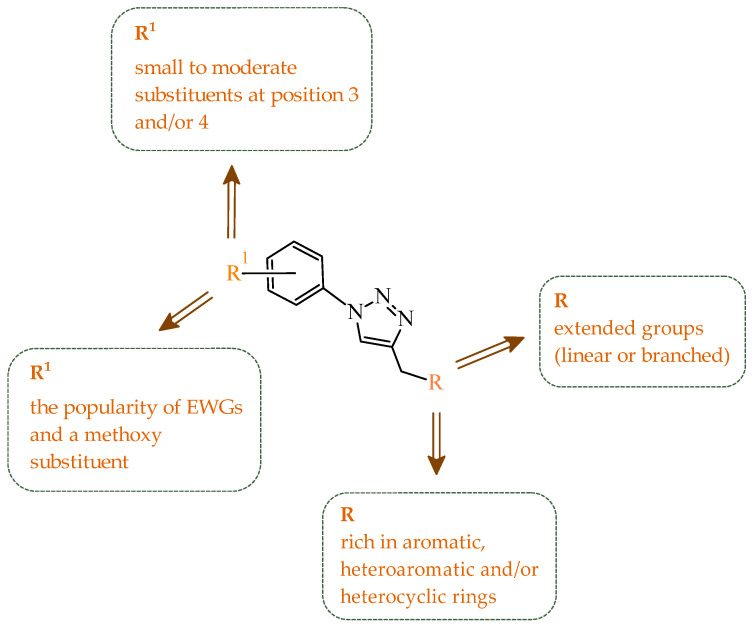
Common features of 1,2,3-triazole-based potential multi-target directed ligands.

1,2,3-Triazole-based systems play a distinct and highly functional role in multi-target anticancer drug design. They are known to act as structural linkers and pharmacophoric connectors rather than dominant binding motifs. In particular, triazole rings are widely utilized due to their synthetic accessibility, metabolic stability, and ability to serve as amide bioisosteres, making them ideal components in hybrid molecular architectures. The dominant isomeric form in drug design is 1*H*-1,2,3-triazole. The incorporation of this motif into multitarget-directed ligands enables the efficient combination of different pharmacophoric units within a single molecule, facilitating simultaneous interaction with multiple biological targets. Furthermore, 1,4-disubstituted 1,2,3-triazole derivatives often exhibit improved biological activity due to their favorable linear geometry, which facilitates optimal spatial arrangement of building blocks. 1,2,3-triazole-based systems showed a preference for kinases such as EGFR, VEGFR-2 and CDK2. Based on some of the most prominent structures, some general SAR conclusions can be drawn (Figure 48). Structure-activity relationship analysis indicates that biological activity is strongly influenced by the nature of substituents attached to the triazole ring, as well as the position of substitution. Compounds with multi-target potential were often characterized by a *N*-1 atom connected to a phenyl ring. In the series selected from the literature, different substitutions on this ring were tested. The most active variants were substituted at positions 3 and/or 4 with small to moderately bulky substituents, such as chlorine atoms, nitro or acetyl groups. Choosing appropriate substituents and their positions was crucial for the multi-target activity of these compounds. In silico studies showed that the phenyl-triazole motif, in some cases, also provided crucial interactions in binding to molecular targets, indicating that the 1,2,3-triazole motif, in addition to acting as a linker, could also serve as a part of the pharmacophore. Active compounds on the opposite side of the triazole motif had a linker connected to extended fragments, often composed of aromatic, heteroaromatic or/and heterocyclic rings. Some of them also contained additional linkers either instead of one of the rings or between them. The length of the linker and the extent of the fragment also influenced the activity of the compounds and the orientation of the molecule in the binding side cavity. Interestingly, among the most active compounds were also molecules containing a sugar moiety, where xylopyranose yielded the best results. All lead compounds based on the 1,2,3-triazole moiety are listed in Table 3, together with a comparison of key SAR features within each series.

**Figure 49:**
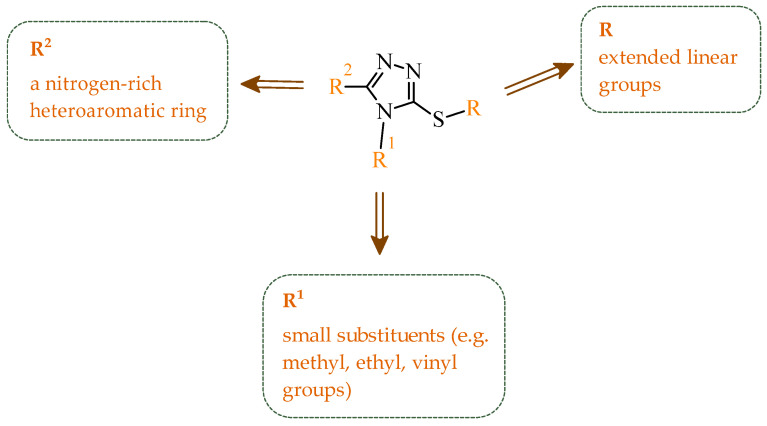
Common features of 4*H*-1,2,4-triazole-based potential multi-target directed ligands.

The 1,2,4-triazole ring serves as a pharmacophore itself and, similarly to 1,2,3-triazole, can also act as a linker between two pharmacophores. However, in contrast to 1,2,3-triazole, due equally common isomeric forms, it enables the design of both linear and branched (with the triazole motif in the center) structures. This allows for the design of molecules with appropriate orientations and shapes for specific binding sites. The most active 1,2,4-triazole derivatives can be divided into two groups depending on the present isomeric forms. In this review, the more common form was 4*H*-1,2,4-triazole, for which the major SAR characteristics are shown in Figure 49. The *N*-1 position of 4*H*-1,2,4-triazole was generally occupied by small substituents such as methyl, ethyl or vinyl groups—bulky substituents were predominantly unfavorable. Enhanced activity was observed for compounds bearing nitrogen-containing aromatic rings at the *C*-5 position, such as pyridine, indole or quinoline. The sulfur atom of the triazole ring was connected to extended fragments, mostly linear, rich in hydrogen bonds donors and acceptors, which increased the activity of the molecules. Due to spatial arrangement, the 3,5-disubstituted 4*H*-1,2,4-triazole motif is often located in the kinase hinge region. There were also potent compounds based on 1*H*-1,2,4-triazoles with irregular activity patterns. A common feature was the presence of one large substituent, composed, among others, of an amide or ureido linker and an aromatic ring, which provided strong interactions within the binding pocket. The key substituents were attached to different atoms of the 1*H*-1,2,4-triazole moiety. For both isomeric forms, the length of the linker was crucial for achieving optimal multi-target activity. The range of molecular targets for the 1,2,4-triazole system was more diverse than for 1,2,3-triazole. However, as in the previous system, the most common kinases were VEGFR-2, EGFR and CDK2. The collected compounds based on the 1,2,4-triazole moiety are summarized in Table 4, together with a comparison of key SAR features within each series.

## 3. Conclusions

Multitargeting activity can result from either pharmacophore hybridization or scaffold functionalization, as observed for the heterocyclic systems discussed here. In this context, imidazole cores often act as central binding motifs, while triazole units often function as structural linkers, enabling the integration of multiple pharmacophore elements. The data collected and discussed highlight the potential of five-membered nitrogen-containing heterocycles (imidazole, pyrazole, and triazole) as multitarget ligands in cancer therapy. As demonstrated in this review, imidazole, pyrazole, and triazole derivatives can act as multi-inhibitors themselves. They can form multimodal compounds when conjugated with another inhibitor. They can also serve as a linker between two pharmacophores in multi-inhibitor compounds. A key factor determining activity is the linker design, including its length, flexibility, and polarity, which determines the spatial arrangement of pharmacophore fragments and their ability to engage different binding sites. Flexible linkers increase adaptability but can result in entropic losses, whereas rigid linkers improve spatial definition at the expense of limited conformational freedom. Some potential anticancer molecules discussed even contained more than one of these heterocyclic scaffolds. Many of the described molecules demonstrated superior or comparable efficacy to reference drugs. Selected derivatives demonstrated inhibitory activity against two or more molecular targets. Importantly, effective MTDLs require balanced activity against different targets, not maximum efficacy against a single protein. The wide range of molecular targets has revealed numerous pathways and approaches to combating cancer. However, certain precautions should be taken, as many of these targets also play important roles in healthy cells. The most popular targets for five-membered nitrogen-based heterocycles were EGFR and VEGFR, key tyrosine kinase receptors that promote cancer cell proliferation and survival, and the formation of new blood vessels. CDK2 and COX-2 were also common targets. SAR analysis of these series and in silico studies of lead compounds help identify patterns that can guide the further design of improved multitarget anticancer compounds. However, these new compounds require further in vivo testing.

## Figures and Tables

**Table 1:** Representative imidazole-based compounds selected as potential multitarget anticancer agents.

Compd.	Structure	Targets	Activity (IC_50_/*K_i_*)	Key SAR Structural Features
**1** (17)	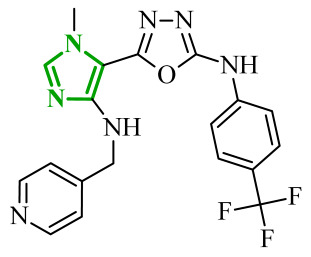	VEGFR-1 VEGFR-2	IC_50_ = 0.12 µM IC_50_ = 0.073 µM	Electron-withdrawing groups at the *para* position (mainly trifluoromethyl) of the phenyl ring
**2** (5a)	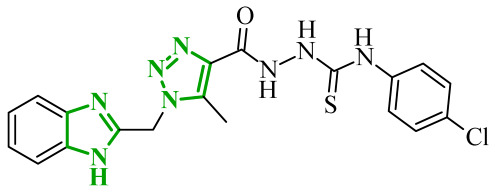	VEGFR-2 EGFR Topo II	IC_50_ = 0.107 µM IC_50_ = 0.086 µM IC_50_ = 2.52 µM	The five-atom linker and halogen substitution at the *para* position of the phenyl ring
**3** (6g)	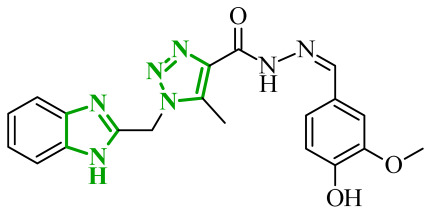	VEGFR-2 EGFR Topo II	IC_50_ = 0.229 µM IC_50_ = 0.131 µM IC_50_ = 8.37 µM	The 4-hydroxy-3-methoxyphenyl ring
**4** (8l)	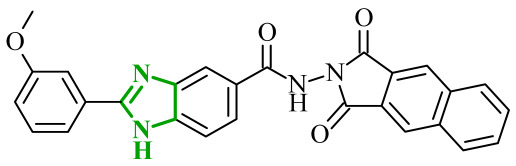	VEGFR-2 FGFR-1	Inhibitory rate 87.61% at 10 µM Inhibitory rate 84.20% at 10 µM	The 1,3-dioxo-1,3-dihydro-2*H*-benzo[*f*]isoindol-2-yl moiety and the metoxy group at the *meta* position of the phenyl ring
**5** (8m)	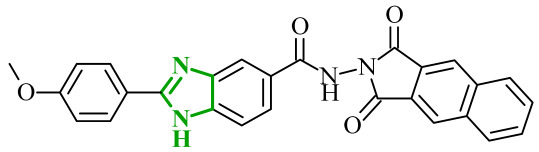	VEGFR-2 FGFR-1	Inhibitory rate 80.69% at 10 µM Inhibitory rate 76.83% at 10 µM	The 1,3-dioxo-1,3-dihydro-2*H*-benzo[*f*]isoindol-2-yl moiety and the metoxy group at the *para* position of the phenyl ring
**6** (8u)	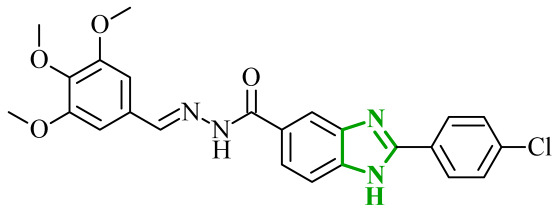	VEGFR-2 FGFR-1 BRAF	IC_50_ = 0.93 µM IC_50_ = 3.74 µM IC_50_ = 0.25 µM	The chlorine atom at the *para* position of the phenyl ring attached to the benzimidazole moiety and the 2,3,4-trimethoxyphenyl ring on the other side of the molecule
**7** (8v)	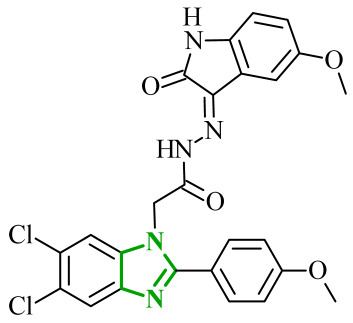	CDK2 GSK-3β	IC_50_ = 0.04 µM IC_50_ = 0.021 µM	The methoxy group at the *para* position of the phenyl ring and the methoxy substituent on the oxindole fragment
**8** (14k)	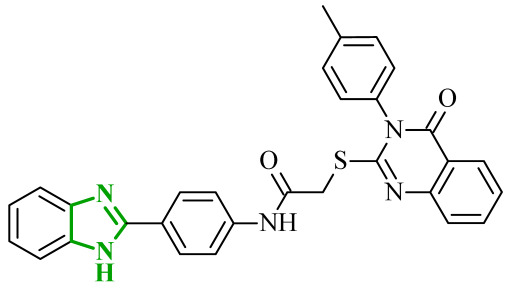	VEGFR-2 CRAF BRAF^wt^ BRAF^V600E^	IC_50_ = 6.14 µM IC_50_ = 10.83 µM IC_50_ = 6.74 µM IC_50_ = 2.47 µM	The quinazolinone fragment with the *p*-methylphenyl substituent
**9** (6c)	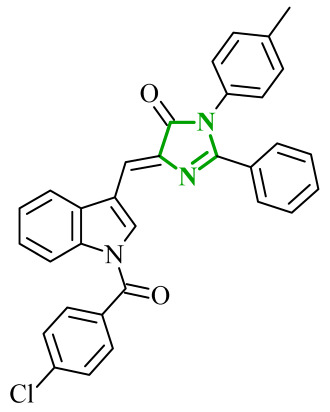	Mcl-1 COX-2	*K_i_* = 0.03 µM IC_50_ = 0.94 µM	The *p*-chlorobenzoyl substituent on the indole ring
**10** (42)	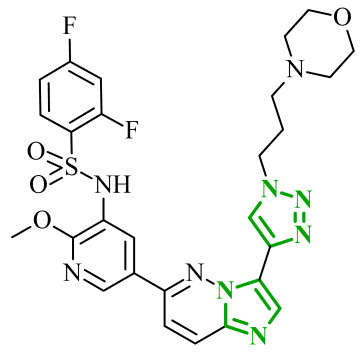	PI3Kα mTOR	IC_50_ = 0.06 nM IC_50_ = 3.12 nM	The imidazo[1,2-*b*]pyridazine system with the triazole linker and the difluorophenyl group in the benzenesulfonamide part
**11** (B11)	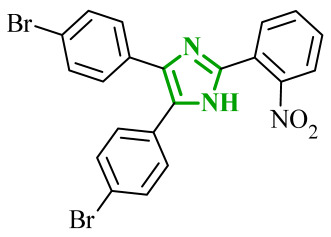	EGFR HER2	-	The *o*-nitrophenyl substitution and two p-bromophenyl groups
**12** (B12)	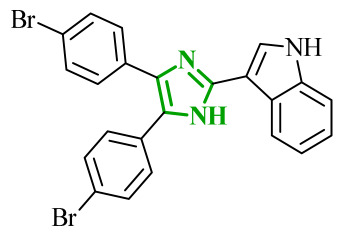	EGFR HER2	-	The indole substitution and two p-bromophenyl groups
**13** (4ACP)	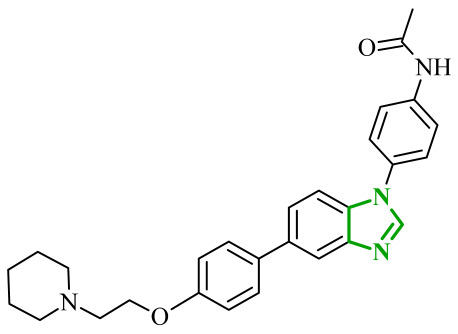	FLT3^wt^ FLT3-ITD FLT3-D835Y TRKA	IC_50_ = 43.8 nM IC_50_ = 97.2 nM IC_50_ = 92.5 nM IC_50_ = 23.6 nM	The benzimidazole core and the piperidino-ethoxy fragment

**Table 2:** Representative pyrazole-based compounds selected as potential multitarget anticancer agents.

Compd.	Structure	Targets	Activity (IC_50_/*K_i_*)	Key SAR Structural Features
**14** (14g)	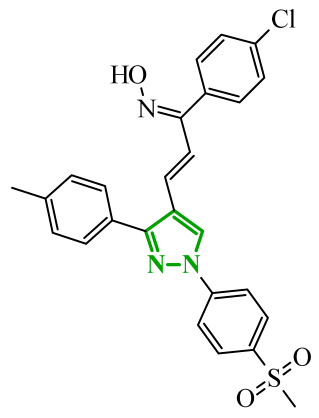	EGFR^wt^ EGFR ^L858R/T790M^ COX-2	IC_50_ = 0.487 µM IC_50_ = 0.041 µM IC_50_ = 0.250 µM	The imino-chalcone fragment and the bromine atom at the *para* position of the phenyl ring
**15** (17c)	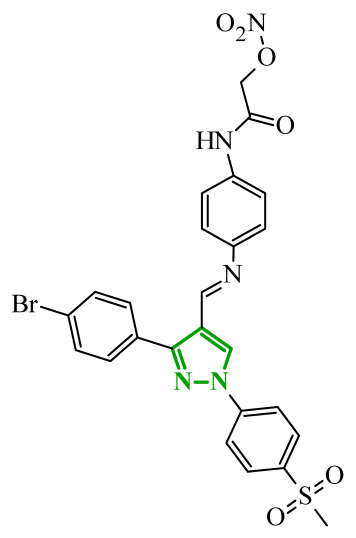	EGFR^wt^ EGFR ^L858R/T790M^ COX-2	IC_50_ = 0.452 µM IC_50_ = 0.031 µM IC_50_ = 0.437 µM	The nitrate with the imino-amido system and the bromine atom at the *para* position of the phenyl ring
**16** (11a)	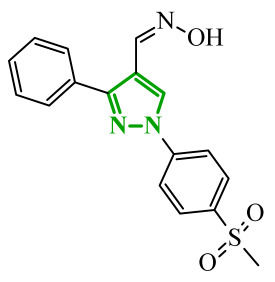	aromatase COX-2	IC_50_ = 16.50 µM IC_50_ = 0.23 µM	The diaryl pyrazole core and the oxime fragment
**17** (10a)	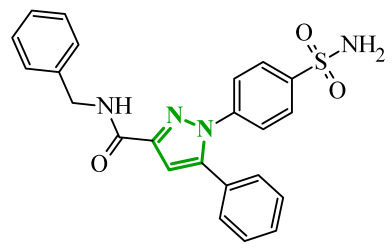	EGFR COX-2	IC_50_ = 6.0 µM IC_50_ = 50 µM	The sulfonamide group at the *para* position of the phenyl ring and the benzylamine part
**18** (6)	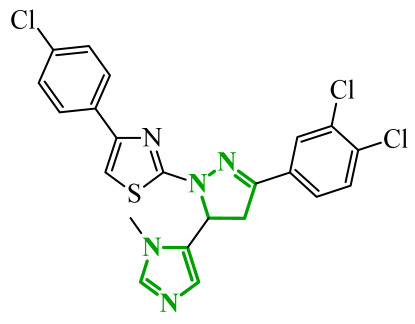	ERK RIPK3	-	The thiazole ring attached to the main core and the phenyl group with the *p*-chloro substituent
**19** (10c)	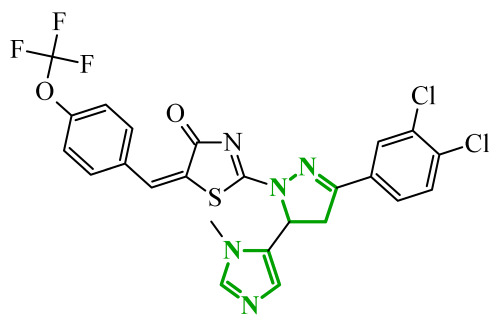	ERK RIPK3	-	The thiazole ring attached to the main core and the benzylidene fragment with the *p*-trifluoro substituent
**20** (10d)	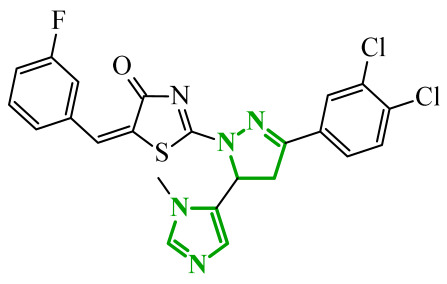	ERK RIPK3	-	The thiazole ring attached to the main core and the benzylidene fragment with the *m*-fluoro substituent
**21** (14c)	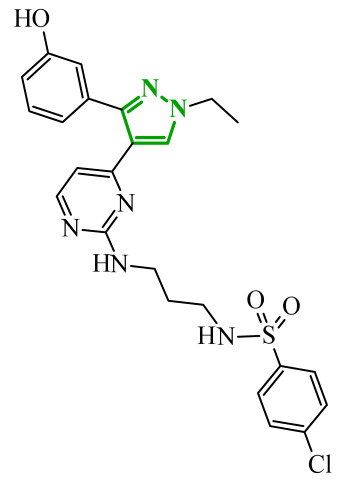	JNK1 JNK2 JNK3 BRAF^V600E^	IC_50_ = 0.51 µM IC_50_ = 0.53 µM IC_50_ = 1.02 µM IC_50_ = 0.009 µM	The hydroxy group at the *meta* position of the phenyl ring (the pyrazole fragment) and the phenyl ring with small to moderate halogen substitution
**22** (14d)	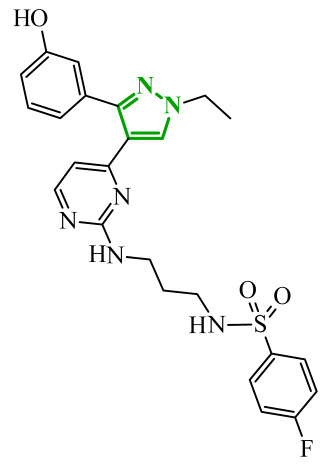	JNK1 JNK2 JNK3 BRAF^V600E^	IC_50_ = 0.55 µM IC_50_ = 0.58 µM IC_50_ = 1.31 µM IC_50_ = 0.011 µM	The hydroxy group at the *meta* position of the phenyl ring (the pyrazole fragment) and the phenyl ring with small to moderate halogen substitution
**23** (6b)	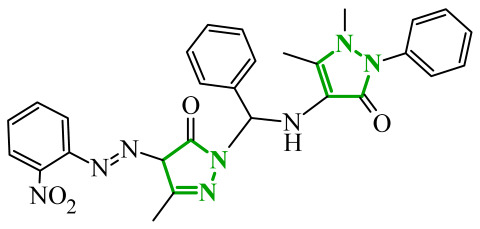	VEGFR-2 CDK2	IC_50_ = 0.2 µM IC_50_ = 0.458 µM	The arylazo substituent with the *o*-nitro group and the antipyrine fragment
**24** (2)	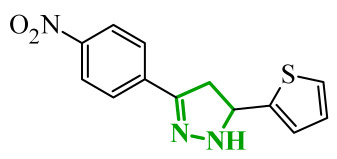	VEGFR-2 EGFR^wt^ EGFR^T790M^	IC_50_ = 242.94 µg/mL IC_50_ = 16.25 µg/mL IC_50_ = 17.8 µg/mL	The unsubstituted pyrazole fragment at the *C*-4 position
**25** (14)	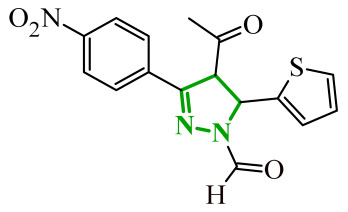	VEGFR-2 EGFR^wt^ EGFR^T790M^	IC_50_ = 112.36 µg/mL IC_50_ = 16.33 µg/mL IC_50_ = 16.6 µg/mL	The small electron-withdrawing substituent (the acetyl group) at the *C*-4 position of the pyrazole fragment
**26** (3f)	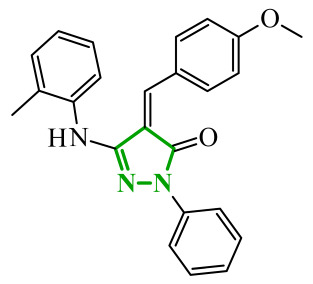	EGFR VEGFR-2	IC_50_ = 0.073 µM IC_50_ = 0.102 µM	The amino group at the *C*-5 position of the pyrazole ring and the unsubstituted phenyl ring
**27** (15g)	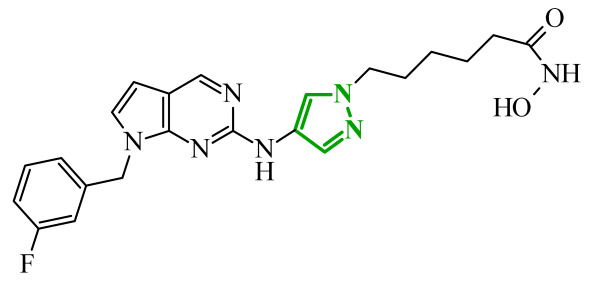	JAK2 HDAC6	IC_50_ = 4.1 nM IC_50_ = 13.7 nM	The five-methylene linker and the fluorine atom at the *meta* position of the benzyl ring
**28** (23k)	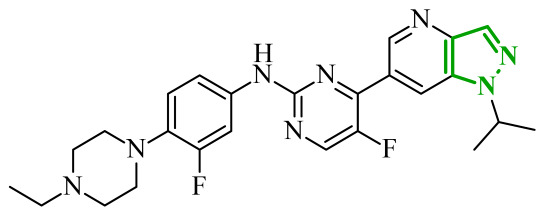	FLT3 CDK4	IC_50_ = 11 nM IC_50_ = 7 nM	The bicyclic system and the piperazine fragment

**Table 3:** Representative 1,2,3-triazole-based compounds selected as potential multitarget anticancer agents.

Compd.	Structure	Targets	Activity (IC_50_/*K_i_*)	Key SAR Structural Features
**29** (13a)	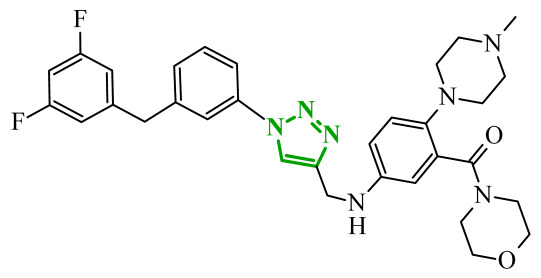	TRKA ALK ALK^L1196M^	IC_50_ = 1.9 nM IC_50_ = 7.2 nM IC_50_ = 65.2 nM	The *N*-methylpiperazine and the morpholine moiety
**30** (9j)	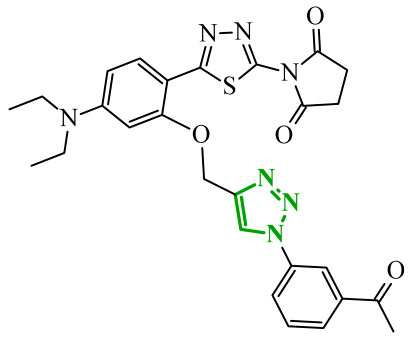	ARK-A ERK2	IC_50_ = 0.018 µM IC_50_ = 0.017 µM	The acetyl group at the *meta* position of the phenyl ring
**31** (5e)	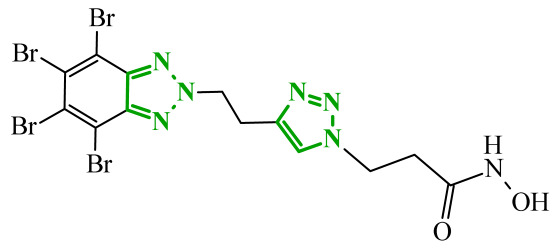	HDAC1 CK2α	Inhibitory rate 97.0% at 50 µM Inhibitory rate 61.6% at 50 µM	Two linkers with two methylene groups on both sides of the triazole ring
**32** (9f)	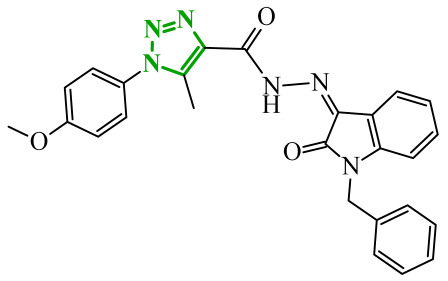	VEGFR-2 STAT-3	IC_50_ = 26.3 nM IC_50_ = 5.63 nM	Benzyl substitution on the isatin fragment and the methoxy group at the *para* position of the phenyl group
**33** (17)	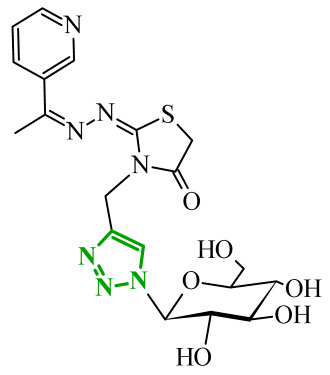	EGFR CDK2	IC_50_ = 0.19 µM IC_50_ = 0.22 µM	The β-d-glucopyranose moiety
**34** (18)	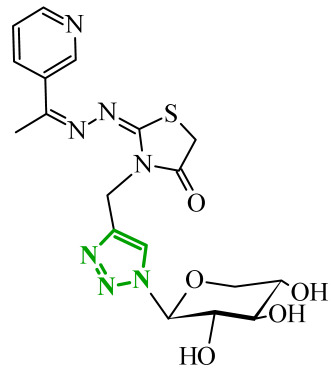	EGFR CDK2	IC_50_ = 0.12 µM IC_50_ = 0.18 µM	The β-d-xylopyranose moiety
**35** (5a)	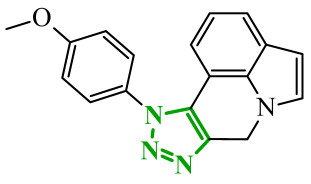	EGFR HER2	IC_50_ = 0.12 µM IC_50_ = 0.34 µM	The methoxy group at the *para* position of the phenyl ring
**36** (5j)	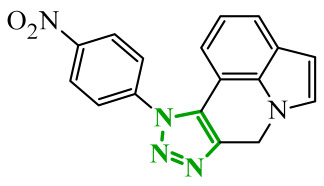	EGFR HER2	IC_50_ = 0.29 µM IC_50_ = 0.72 µM	The nitro group at the *para* position of the phenyl ring
**37** (5g)	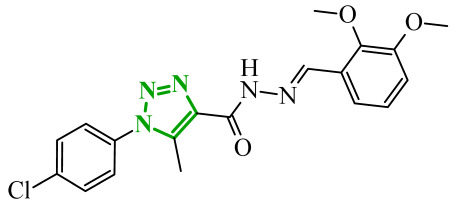	EGFR telomerase	IC_50_ = 3.45 ng/mL IC_50_ = 1.31 ng/mL	The chlorine atom at the *para* position of the phenyl ring and 3,4-dimethoxy substitution on the benzelidene part
**38** (4a)	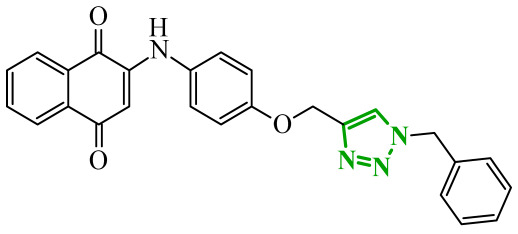	CDK2 VEGFR-3 PDGFRA	IC_50_ = 1.672 µM IC_50_ = 0.554 µM IC_50_ = 0.629 µM	The unsubstituted benzyl fragment
**39** (7)	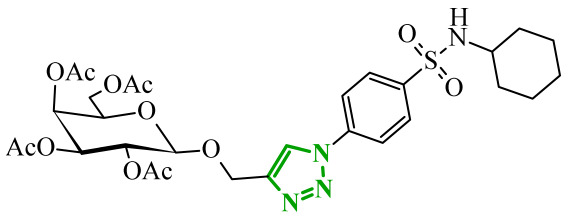	VEGFR-2 hCA IX hCA XII	IC_50_ = 1.33 µM IC_50_ = 66 nM IC_50_ = 7.6 nM	The peracetylated β-d-galactopyranose moiety
**40** (9)	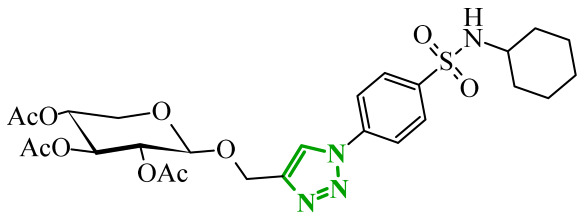	VEGFR-2 hCA IX hCA XII	IC_50_ = 0.38 µM IC_50_ = 40 nM IC_50_ = 3.2 nM	The peracetylated β-d-xylopyranose moiety
**41** (TI12)	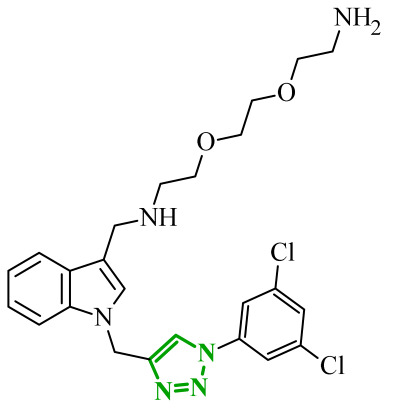	*c-KIT* *KRAS* genes	-	The 2-(2-(2-aminoethoxy)ethoxy fragment and the 3,5-dichlorophenyl ring

**Table 4:** Representative 1,2,4-triazole-based compounds selected as potential multitarget anticancer agents.

Compd.	Structure	Targets	Activity (IC_50_/*K_i_*)	Key SAR Structural Features
**42** (6a)	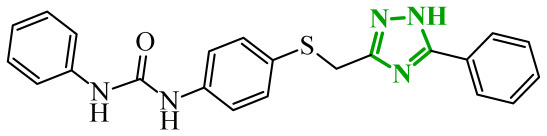	c-KIT AurB	Inhibitory rate 78% at 10 µM Inhibitory rate 92% at 10 µM	Two unsubstituted phenyl rings
**43** (13b)	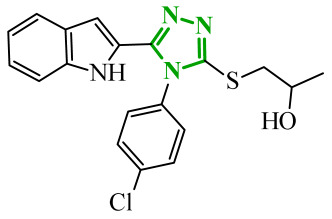	EGFR PARP-1	IC_50_ = 62.4 nM IC_50_ = 1.24 nM	The 2-hydroxypropyl group and the chlorine atom at the *para* position the of phenyl ring
**44** (10a)	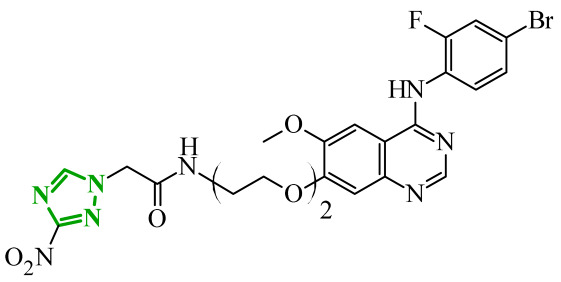	EGFR VEGFR-2	IC_50_ = 5.90 nM IC_50_ = 36.78 nM	The 4-bromo-2-fluoroaniline fragment and the optimal linker length (two ethoxy groups)
**45** (6a)	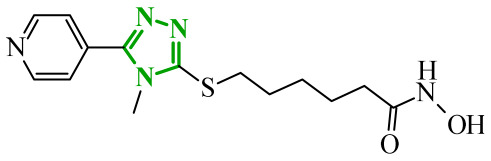	HDAC2 FAK	IC_50_ = 0.09 µM IC_50_ = 12.59 nM	The *N*-methylated triazole ring and the hydroxamic acid attached via an aliphatic linker
**46** (8)	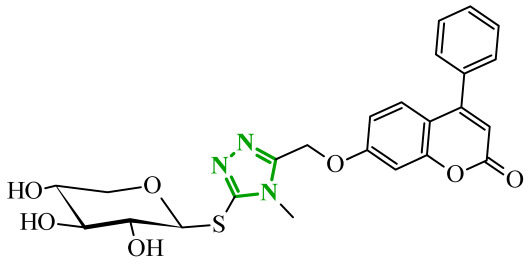	EGFR VEGFR-2 CDK2	IC_50_ = 0.22 µM IC_50_ = 0.93 µM IC_50_ = 0.24 µM	The *N*-methylated triazole ring and the β-d-xylopyranose moiety
**47** (10)	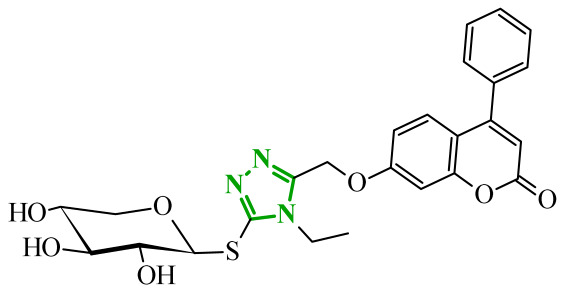	EGFR VEGFR-2 CDK2	IC_50_ = 0.12 µM IC_50_ = 0.79 µM IC_50_ = 0.15 µM	The *N*-ethylated triazole ring and the β-d-xylopyranose moiety
**48** (8f)	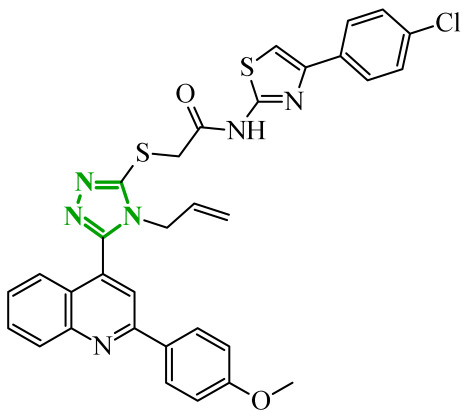	EGFR HER2 BRAF^V600E^	IC_50_ = 67 nM IC_50_ = 33 nM IC_50_ = 49 nM	The chlorine atom at the *para* position of the phenyl ring (the thiazole ring) and the methoxy group at the *para* position of the phenyl ring (the quinoline fragment)
**49** (13a)	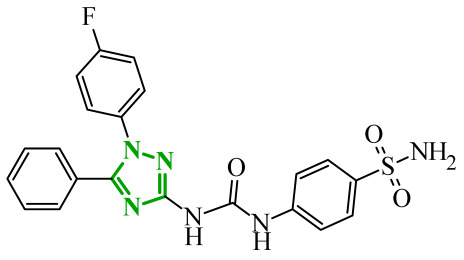	VEGFR-2 hCA IX hCA XII	IC_50_ = 26.3 nM *K_i_* = 8.3 nM *K_i_* = 4.7 nM	The sulfamoyl group at the *para* position of the phenyl ring (the ureido fragment) and the fluorine atom at the *para* position of the phenyl ring (the triazole moiety)

## Data Availability

No new data were created or analyzed in this study. Data sharing is not applicable to this article.
